# Quantum-secure covert communication on bosonic channels

**DOI:** 10.1038/ncomms9626

**Published:** 2015-10-19

**Authors:** Boulat A. Bash, Andrei H. Gheorghe, Monika Patel, Jonathan L. Habif, Dennis Goeckel, Don Towsley, Saikat Guha

**Affiliations:** 1 Quantum Information Processing Group, Raytheon BBN Technologies, 10 Moulton Street, Cambridge, Massachusetts 02138, USA; 2 College of Information and Computer Sciences, University of Massachusetts, Amherst, Massachusetts 01003, USA; 3 Amherst College, Amherst, Massachusetts 01002, USA; 4 Electrical and Computer Engineering Department, University of Massachusetts, Amherst, Massachusetts 01003, USA

## Abstract

Computational encryption, information-theoretic secrecy and quantum cryptography offer progressively stronger security against unauthorized decoding of messages contained in communication transmissions. However, these approaches do not ensure stealth—that the mere presence of message-bearing transmissions be undetectable. We characterize the ultimate limit of how much data can be reliably and covertly communicated over the lossy thermal-noise bosonic channel (which models various practical communication channels). We show that whenever there is some channel noise that cannot in principle be controlled by an otherwise arbitrarily powerful adversary—for example, thermal noise from blackbody radiation—the number of reliably transmissible covert bits is at most proportional to the square root of the number of orthogonal modes (the time-bandwidth product) available in the transmission interval. We demonstrate this in a proof-of-principle experiment. Our result paves the way to realizing communications that are kept covert from an all-powerful quantum adversary.

## Abstract (web summary)

Communications encryption does prevent unauthorized decoding, but does not ensure stealth of the transmission. Here, the authors characterize the quantum limits of covert communication on lossy thermal-noise bosonic channels.

## Graphical abstract

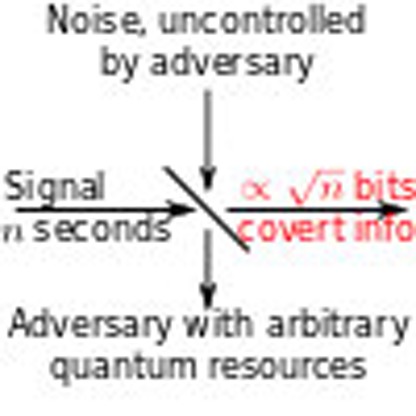

Encryption prevents unauthorized access to transmitted information—a security need critical to modern-day electronic communication. Conventional computationally secure encryption^1^,^2^, information-theoretic secrecy^3^,^4^,^5^ and quantum cryptography^6^ offer progressively higher levels of security. Quantum key distribution (QKD) allows two distant parties to generate shared secret keys over a lossy–noisy channel that are secure from the most powerful adversary allowed by physics. This shared secret, when subsequently used to encrypt data using the one-time-pad cipher^7^, yields the most powerful form of encryption. However, encryption does not mitigate the threat to the users' privacy from the discovery of the very existence of the message itself, nor does it provide the means to communicate when the adversary forbids it. Thus, low probability of detection/intercept, or covert communication systems are desirable, which not only protect the message content but also prevent the detection of the transmission attempt.

Covert communication is an ancient discipline revived by the communication revolution of the last century. Modern developments include spread-spectrum radiofrequency communication^8^, where the signal power is suppressed below the noise floor by bandwidth expansion, and steganography^9^, where messages are hidden in fixed-size, finite-alphabet cover text objects such as digital images. Performance of classical communication is typically quantified using Shannon capacity^10^: the maximum rate (in bits/channel use) at which classical data can be reliably transmitted over a noisy channel in the limit of infinite channel uses. However, our recent work on classical covert communication over the additive white Gaussian noise (AWGN) channels (the standard model for radiofrequency channels) shows that the sender Alice can reliably transmit 
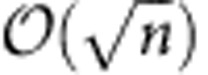
 bits to the intended receiver Bob in *n* AWGN channel uses with arbitrarily low probability of detection by the adversary Willie, who receives Alice's transmissions over a separate AWGN channel^11^,^12^. Therefore, the communication rate approaches zero as *n*→∞. Shannon capacity is thus inadequate for characterizing the limits of covert communication over the AWGN channel, and we developed new techniques to prove this square-root law (SRL). Since then, alternative techniques and insights, as well as further refinements to the SRL, have been found^13^,^14^. Even though the asymptotic rate of covert communication is zero (as 
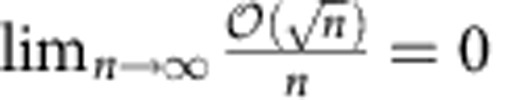
), a non-trivial burst of covert bits can be transmitted when *n* is large. Our work was generalized to other classical channel settings^13^,^14^,^15^,^16^,^17^,^18^. Similar SRLs were also found in steganography, where it was shown that Alice can modify 
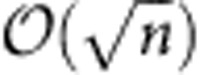
 symbols in a cover text of size *n*, embedding 
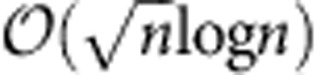
 hidden bits^9^,^19^,^20^,^21^,^22^,^23^. The log *n* improvement in the steganographic application versus covert communication over a noisy channel is attributable to the noiseless Alice-to-Bob channel. Recent work shows that an empirical model of cover text suffices to break the steganographic SRL^24^; however, these results do not apply to the covert communication channels discussed here.

The lossy thermal-noise bosonic channel is the quantum mechanical description of the transmission of a single (spatio-temporal polarization) mode of the electromagnetic field at a given transmission wavelength (such as optical or microwave) over linear loss and additive Gaussian noise (such as noise stemming from blackbody radiation). Modern high-sensitivity communication components—such as sources and detectors—are primarily limited by noise of quantum-mechanical origin, in particular at optical frequencies. Thus, recent studies quantified the ultimate rate of reliable and secure communication using tools from quantum information theory^25^,^26^,^27^,^28^. Our analysis of covert communication over AWGN channels already applies to communication over lossy thermal-noise bosonic channels when Alice uses laser-light modulation and both Bob and Willie use coherent-detection (homodyne or heterodyne) receivers, as these choices of transmitter and receiver structures induce AWGN channels.

However, quantum mechanics permits a much wider class of transmitter and receiver measurements. Therefore, delineating the ultimate limits of covert communications on the lossy thermal-noise bosonic channel that is secure against the most powerful adversary physically permissible requires quantum information-theoretic analysis. Here we establish these limits. We demonstrate that covert communication is impossible when the adversary has full control over the noise in the channel. However, any excess noise that is not controlled by the adversary (for example, the unavoidable thermal noise from the blackbody radiation at the operating temperature) allows Alice to reliably transmit 
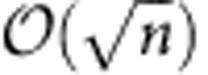
 covert bits to Bob using *n* bosonic modes, even if Willie intercepts all the photons not reaching Bob and employs arbitrary quantum memory and measurements. Furthermore, this is achievable using standard laser-light modulation and homodyne detection (that is, the Alice–Bob channel is still an AWGN channel). Thus, noise enables stealth. Indeed, if Willie's detector contributes excess noise (for example, dark counts in photon-counting detectors), Alice can covertly communicate to Bob, even when the channel itself is pure loss. We also show that the SRL cannot be circumvented. Thus, the covert communication rate is zero and the Holevo capacity^29^—the generalization of the Shannon capacity for the classical capacity of quantum channels—is inadequate for our analysis. We corroborate our theoretical results with a proof-of-concept experiment done at 1.55 μm, where the adversary-uncontrolled excess noise is emulated by dark current of Willie's detector. We thus demonstrate a truly information-theoretically secure covert communication system that allows communication when all transmissions are prohibited. Finally, we employ a realistic model of loss and noise in atmospheric propagation to calculate the achievable volume of data transmissible covertly over a 1-km-range line-of-sight channel as a function of the transmission wavelength and the transceiver contact duration, where the covertness solely relies on the minimum amount of thermal noise that must arise from blackbody and solar radiation at the operating temperature and wavelength. Our analysis provides evidence for optimality of long-wave infrared (LWIR) wavelengths for quantum-secure covert communication.

## Results

### Information-theoretically covert communication

Quantum and classical information-theoretic analyses of covert communication consider the reliability and detectability of a transmission. We introduce these concepts next.

We consider a scenario where Alice attempts to transmit *M* bits to Bob using *n* bosonic modes, whereas Willie attempts to detect her transmission attempt. We treat a single spatio-temporal polarization mode of the electromagnetic field as the fundamental transmission unit over the channel (a more formal description of modes is provided in the Supplementary Note 1). Each of the 2^*M*^ possible *M*-bit messages maps to an *n*-mode codeword and their collection forms a codebook. Desirable codebooks ensure that the codewords, when corrupted by the channel, are distinguishable from one another. This provides reliability: a guarantee that the probability of Bob's error in decoding Alice's message 
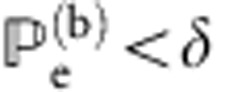
 with arbitrarily small *δ*>0 for large *n*. In practice, error-correction codes are used to enable reliability.

Willie's detector reduces to a binary hypothesis test of Alice's transmission state given his observations of the channel. Denote by 
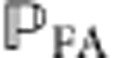
 the probability that Willie raises a false alarm when Alice does not transmit and by 
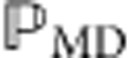
 the probability that Willie misses the detection of Alice's transmission. Under the assumption of equal prior probabilities on Alice's transmission state, Willie's detection error probability is 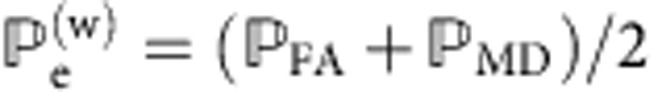. Alice desires a reliable signalling scheme that is covert; in other words, ensures 
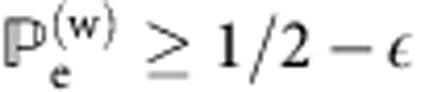
 for an arbitrarily small *ε*>0 regardless of Willie's quantum measurement choice (as 
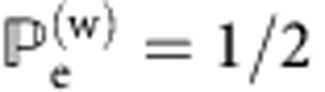
 for a random guess). We show in the Supplementary Note 2 that unequal prior probabilities do not affect our asymptotic results. By decreasing her transmission power, Alice can decrease the effectiveness of Willie's hypothesis test at the expense of reducing the reliability of Bob's decoding. Information-theoretically secure covert communication is provably both reliable and covert. To achieve it, before transmission Alice and Bob share a secret, the cost of which we assume to be substantially less than that of being detected by Willie. Secret sharing is consistent with other information-hiding systems^8^,^9^,^11^,^12^,^19^,^20^,^21^,^22^,^23^ and can be done when Alice and Bob meet in a location that is physically secure from Willie (just as soldiers synchronize the spreading codes and frequency-hopping patterns on their spread-spectrum radios^8^ before leaving the base). The secret can also be generated using standard classical^5^ or quantum^6^ methods if there are periods of time when Willie allows Alice and Bob to communicate. However, as evidenced by recent results for a restricted class of channels^13^,^16^,^17^, we believe that certain scenarios (for example, Willie's channel from Alice being worse than Bob's) will allow secret-less covert communication on bosonic channels.

### Analysis of covert communication on bosonic channels

Here we outline the theoretical development of quantum information-theoretically secure covert communication on the lossy–noisy bosonic channel. Formal statements are deferred to the Methods, with detailed proofs in the Supplementary Note 3.

**Figure 1:**
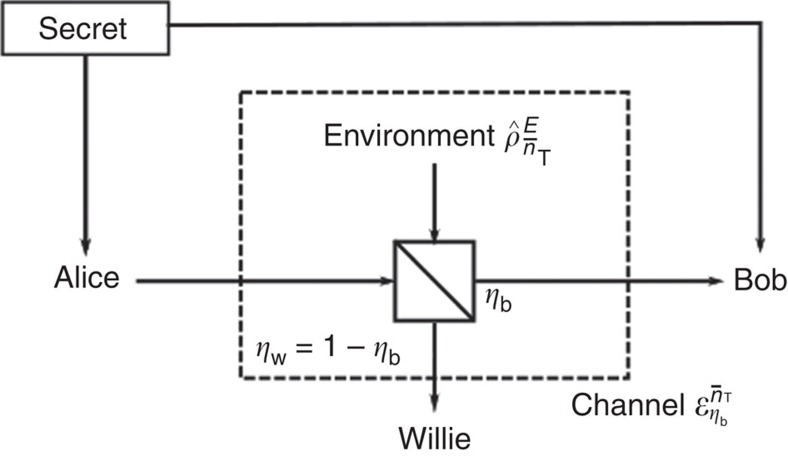
Channel model. The input–output relationship is captured by a beamsplitter of transmissivity *η*_b_, with the transmitter Alice at one of the input ports and the intended receiver Bob at one of the output ports, and *η*_b_ being the fraction of Alice's signalling photons that reach Bob. The other input and output ports of the beamsplitter correspond to the environment and the adversary Willie. Willie collects fraction *η*_w_=1-*η*_b_ of Alice's photons that do not reach Bob. This lossy–noisy bosonic channel accurately models single-spatial-mode free space and single-mode fibre optical channels. Alice and Bob share a secret before the transmission.

Consider a single-mode lossy bosonic channel 
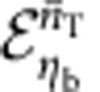
 of transmissivity *η*_b_∈(0, 1] and thermal noise mean photon number per mode 
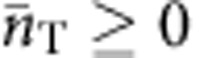
, as depicted in Fig. 1. Willie collects fraction *η*_w_=1−*η*_b_ of Alice's photons that do not reach Bob. Willie is otherwise passive, as we later argue that actively injecting noise into the channel does not help him detect Alice's transmissions. For a pure loss channel 
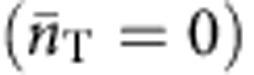
, the environment input is in the vacuum state 
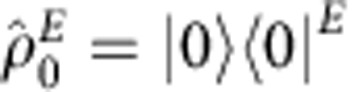
, corresponding to the minimum noise the channel must inject to preserve the Heisenberg inequality of quantum mechanics.

Regardless of Alice's strategy, reliable and covert communication over a pure-loss channel to Bob 
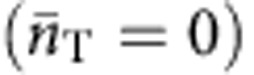
 is impossible. Theorem 1 in the Methods demonstrates that Willie can effectively use an ideal single photon detector (SPD) on each mode to discriminate between an *n*-mode vacuum state and any non-vacuum state in Alice's codebook. Willie avoids false alarms, as no photons impinge on his SPD when Alice is silent. However, a single click—detection of one or more photons—gives away Alice's transmission attempt regardless of the actual quantum state of Alice's signalling photons. Alice is thus constrained to codewords that are nearly indistinguishable from vacuum, rendering unreliable any communication attempt that is designed to be covert. Furthermore, any communication attempt that is designed to be reliable cannot remain covert, as Willie detects it with high probability for large *n*. This is true even when Alice and Bob have access to an infinitely large pre-shared secret. Thus, if Willie controls the environment (as assumed in QKD security proofs), by setting it to vacuum, he can deny covert communication between Alice and Bob. However, some amount of non-adversarial excess noise—whether from the thermal background or the detector itself—is unavoidable, which enables covert communication.

Consider the lossy bosonic channel 
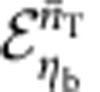
, where the environment mode is in a thermal state with mean photon number 
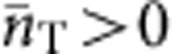
. A thermal state is represented by a mixture of coherent states |*α*〉—quantum descriptors of ideal laser light—weighted by a Gaussian distribution over the field amplitude 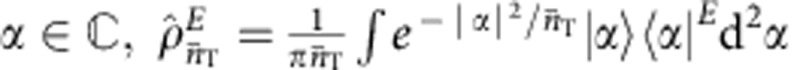. This thermal noise masks Alice's transmission attempt, enabling covert communication even when Willie has arbitrary resources, such as access to all signalling photons not received by Bob and any quantum-limited measurement. Theorem 2 in the Methods demonstrates that in this scenario Alice can reliably transmit 
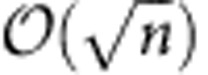
 covert bits using *n* modes to Bob, who needs only a conventional homodyne-detection receiver. Alice achieves this using mean photon number per mode 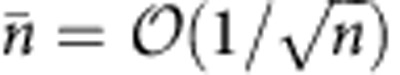. Conversely, Theorem 5 states that if Alice exceeds the limit of 
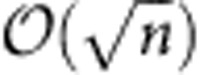
 covert bits in *n* modes, transmission is either detected or unreliable.

Three comments are in order here (with details deferred to the remarks following the proof of Theorem 2 in the Supplementary Note 3). First, we note that only a lower bound on 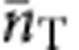 is needed for reliable transmission of 
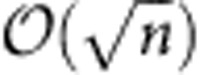
 covert bits using *n* bosonic modes. One can use Planck's law to obtain such a bound given the transmitter's centre frequency and an estimate of Willie's receiver temperature. However, if such a lower bound is unavailable, then 
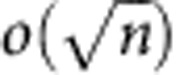
 covert bits can be transmitted. Second, actively injecting noise into the channel does not help Willie reduce the scaling in the SRL, even though he can reduce the number of covert bits that Alice can reliably transmit to Bob by reducing Bob's received signal-to-noise ratio. Finally, thermal noise power plays a critical role in determining how many covert bits can be transmitted. Specifically, we show that, when 
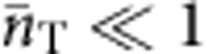
, approximately at least 
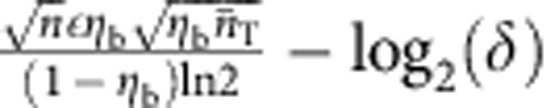
 quantum-secure covert bits can be transmitted, where *δ* and *ε* are the parameters governing the reliability and detectability of the transmission described in the previous section. Thus, low thermal noise power at Willie implies low covert communication volume even when the channel loss is low. On the other hand, high loss on the channel from Alice to Bob implies low covert communication volume, as we assume that all the transmitted photons that do not reach Bob are captured by Willie. The relationships between loss, noise and covert communication volume are analysed over a wide range of centre frequencies in the next section.

Although the lossy thermal-noise bosonic channel is arguably the most important quantum communication channel, to meet the benchmark set by QKD for cryptography, it is necessary to find the conditions for reliable covert communication over a general quantum channel. Even though we defer this problem to future work, here we state it formally, linking it to the theorems proved in this paper.

A trace-preserving completely positive input–output map 
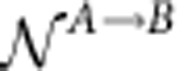
 models a general quantum channel between Alice and Bob. We believe that one can show that, if the ‘environment' of this quantum channel is assumed to be controlled by an adversary in its entirety as is done in the theory of QKD, then covert and reliable communication is impossible. This would generalize our Theorem 1. We also believe that quantum-secure covert communication is possible only if there is a subsystem of the channel's isometric extension that is inaccessible to Willie (this would be the part of the channel noise that no physical adversary can control). This would generalize our SRL. To further illustrate this point, let us consider an Alice-to-Bob bosonic channel with additive thermal noise, but also assume that Willie possesses a ‘purification' of that noise. The joint state held by Willie is an entangled two-mode squeezed vacuum state, each mode of which is locally in a zero-mean thermal state. Then, even though the half of the two-mode squeezed vacuum that gets injected into the channel looks like thermal noise to Alice and Bob, we believe that one can show that covert communication in this case is impossible. This scenario is similar to the one in Theorem 1. It is only when there is some amount of that noise that is not controlled by Willie—which, for instance, is the case for thermal noise from blackbody at the given transmission wavelength and operating temperature—that the SRL for covert communication applies.

### Experimental demonstration of covert communication

Any form of excess noise that is uncontrollable by Willie can be used to hide transmissions for covert communication. In addition to the thermal environment at the operating temperature, this includes, for example, noise from the detector dark current and Johnson noise. We chose to use a laser transmitter at 1.55 μm and SPDs for a proof-of-concept demonstration of the square-root scaling for covert communication over a bosonic channel. As 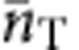 is very small at 1.55 μm, to obtain a statistically significant data set we emulate the adversary uncontrollable excess noise using the dark counts—erroneous detection events stemming from an internal spontaneous emission process and thermal blackbody. First, we extend our theoretical framework for covert communication to when the excess noise stems from dark counts in Willie's detector. The formal theorem statements are in the Methods and their mathematical proofs are in the Supplementary Note 3. We consider the (hypothetical) pure-loss channel, for which Willie's optimal receiver is an ideal SPD on each mode (as discussed in the remark following the proof of Theorem 1 in the Supplementary Note 3). If Willie's detector has a non-zero dark count probability, we show (Theorem 3 in Methods) that using an on–off keying (OOK) coherent state modulation where Alice transmits the ‘on' symbol 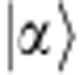 with probability 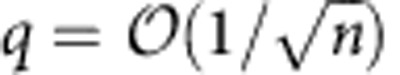 and the ‘off' symbol |0〉 with probability 1−*q* allows her to reliably transmit 
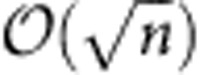
 covert bits using *n* OOK symbols. We also note that our techniques can be used to derive the SRL for other sources/statistics of noise as well.

The skewed on–off duty cycle of OOK modulation makes construction of efficient error correction codes (ECCs) challenging. Constraining OOK signalling to *Q*-ary pulse position modulation (PPM) addresses this issue by sacrificing a small constant fraction of throughput. Each PPM symbol uses a PPM frame to transmit a sequence of *Q* coherent state pulses, 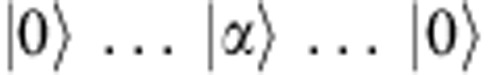, encoding message *i*∈{1, 2,…, *Q*} by transmitting 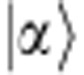 in the *i*th mode of the PPM frame. Thus, instead of 
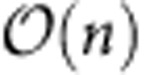
 bits allowed by OOK, PPM lets 
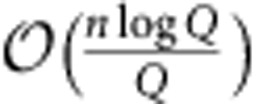
 bits be transmitted in *n* optical modes. However, PPM performs well in the low photon number regime^30^ and the symmetry of its symbols enables the use of many efficient ECCs.

To communicate covertly, Alice and Bob use a fraction 
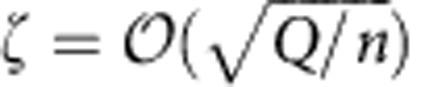
 of *n*/*Q* available PPM frames on average, effectively using 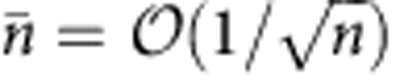 photons per mode. By keeping secret which frames they use, Alice and Bob force Willie to examine them all, increasing the likelihood of dark counts. An ECC, even one that is known to Willie, ensures reliability. However, the transmitted pulse positions are scrambled within the corresponding PPM frames via an operation resembling one-time pad encryption^7^, preventing Willie's exploitation of the ECC's structure for detection (rather than protecting the message content). Theorem 4 demonstrates that, using this protocol, Alice reliably transmits 
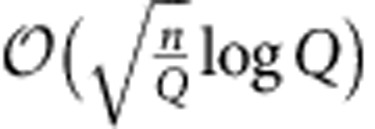
 covert bits at the cost of pre-sharing 
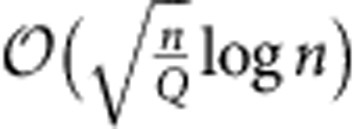
 secret bits.

To demonstrate the square-root scaling of covert communications we employed the protocol from the proof of Theorem 4 in a proof-of-concept test-bed implementation. Alice and Bob engage in an *n*-mode communication session consisting of *n*/*Q Q*-ary PPM frames, *Q*=32. Alice transmits 
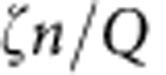
 PPM symbols on average, using a first-order Reed–Solomon (RS) code for error correction. RS codes perform well on channels dominated by erasures, which occur in low receive-power scenarios, for example, covert and deep space communication^31^. Alice and Bob use a (31, 15) RS code. Other modulation/coding schemes may deliver a constant factor improvement in the volume of reliably transmissible covert bits; however, in our experiments RS-over-PPM performs well enough. Optimizing the constant factor in the big-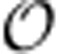 notation is outside the scope of this work. The specifics of the generation of the transmitted signal are in the Methods. We varied *n* from 3.2 × 10^6^ to 3.2 × 10^7^ in several communication regimes: ‘careful Alice' 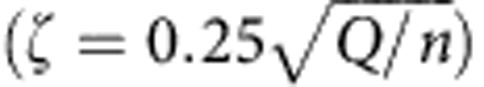, ‘careless Alice' 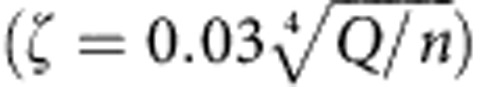 and ‘dangerously careless Alice' (
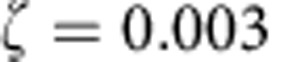
 and 
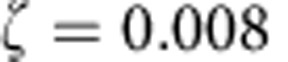
). For each 
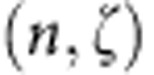
 pair we conducted 100 experiments and 10^5^ Monte-Carlo simulations, measuring Bob's total number of bits received and Willie's detection error probability.

**Figure 2:**
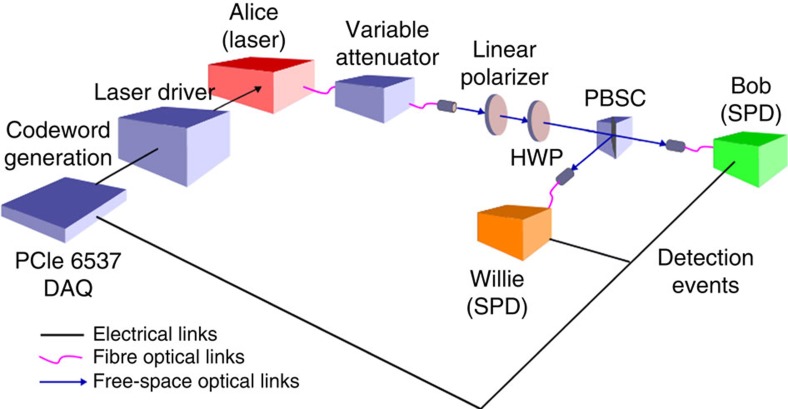
Experimental setup. A National Instruments PCIe-6537 data acquisition card (DAQ), driven by a 1-MHz clock, controlled the experiment, generating transmissions and reading detection events. Alice generated 1 ns optical pulses using a temperature-stabilized laser diode with centre wavelength 1550.2 nm. The pulses were sent into a free-space optical channel, where a half-wave plate (HWP) and polarizing beamsplitter cube (PBSC) sent a fraction *η*_b_ of light to Bob and the remaining light to Willie. Bob and Willie's receivers operated InGaAs Geiger-mode avalanche photodiode SPDs that were gated with 1 ns reverse bias triggered to match the arrival of Alice's pulses.

The experiment was conducted using a mixture of fibre-based and free-space optical elements implementing channels from Alice to both Bob and Willie (see Fig. 2 for a schematic). Although we recognize that this wavelength does not generally enable covert practical communications (owing to the lack of natural noise sources such as solar or thermal background radiation), this was a convenient wavelength at which to conduct proof-of-concept laboratory experiments. We provided noise only during the gating period of the detectors, as continuous wave light irradiating Geiger-mode avalanche photodiodes suppresses detection efficiency^32^. As a substitute to providing external optical noise we emulated optical noise at the detectors by increasing the detector gate voltage, thus increasing the detector's dark click probability. Although the avalanche photodiode dark counts are Poisson distributed with mean rate 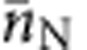 photons per mode, when 
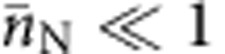
 the dark click probability 
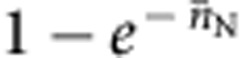
 is close to 
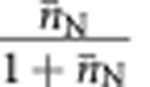
, the probability that an incoherent thermal background with mean photon number per mode 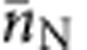 produces a click. Thus, the statistical characteristics of our emulated noise match those of the noise produced by a thermal environment. Table 1 reports the experimentally observed estimates and targeted values of dark click probabilities 
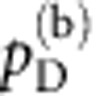
 and 
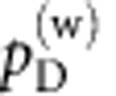
 of Bob's and Willie's detectors, as well as the mean number of photons detected by Bob 
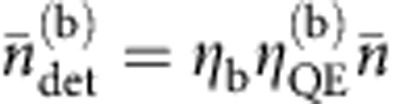
 and Willie 
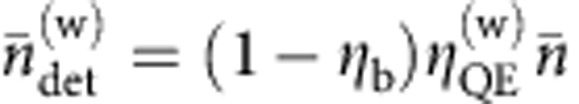
, where 
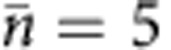
 is the mean photon number of Alice's pulses, *η*_b_=0.97 is the fraction of light sent to Bob and 
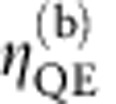
 and 
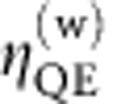
 are the quantum efficiencies of Bob's and Willie's detectors, which we do not explicitly calculate. We provide additional implementation details in the Methods.

The amount of transmitted information, with other parameters fixed, is proportional to 
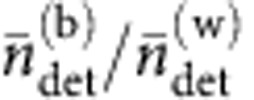
. Our use of emulated noise in Willie's detector as well as a choice of 
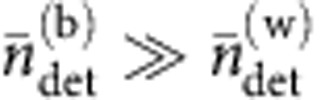
 resulted in Bob's signal-to-noise ratio being substantially higher than Willie's. This allowed the experiment to gather a statistically meaningful data sample to validate the SRL in an experimentally tractable time duration. In an operational free-space laser communication system, a directional transmitter will probably yield such an asymmetry in coupling between Bob and Willie; however, we note that the only fundamental requirement for implementing information-theoretically secure covert communication is 
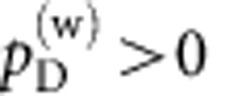
, or 
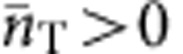
.

**Figure 3:**
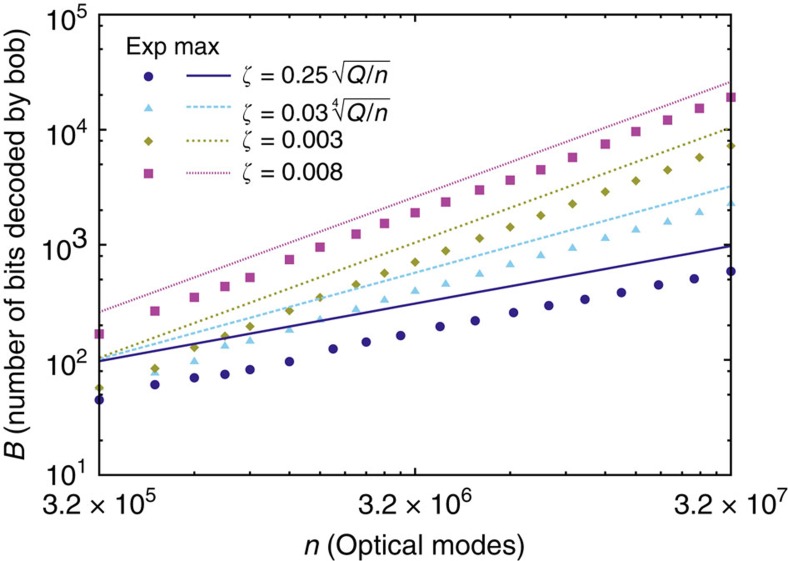
Number of bits decoded by Bob. Each data point is an average from 100 experiments, with negligibly small 95% confidence intervals. The symbol error rates are: 1.1 × 10^−4^ for 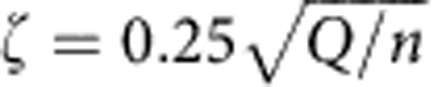, 8.3 × 10^−3^ for 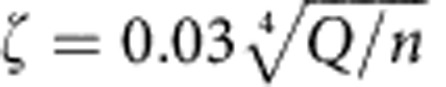, 4.5 × 10^−3^ for 
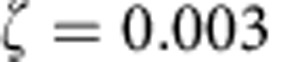
 and 1.8 × 10^−1^ for 
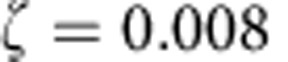
. We also report the maximum number of covert bits that can be decoded by Bob 
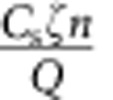
, which is computed in the Methods using the experimentally observed values from Table 1, where *C*_s_ is the per-symbol Shannon capacity^10^. Given the low observed symbol error rate for 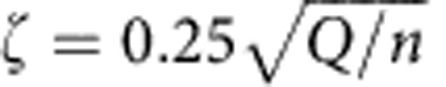, we note that square-root scaling is achievable even using a relatively short RS code; Fig. 4 demonstrates that this is achieved covertly.

Figure 3 reports the number of bits received by Bob from Alice (with the caption reporting the corresponding symbol error rate) in our experiments and the theoretical maximum he could receive (calculated for each regime using the experimentally observed values from Table 1). Details of our analysis are in the Methods. Our relatively short RS code achieves between 45% and 60% of the maximum in the ‘careful Alice' regime and between 55% and 75% of the maximum in other regimes at reasonable error rates, showing that even a basic code demonstrates our theoretical scaling.

**Figure 4:**
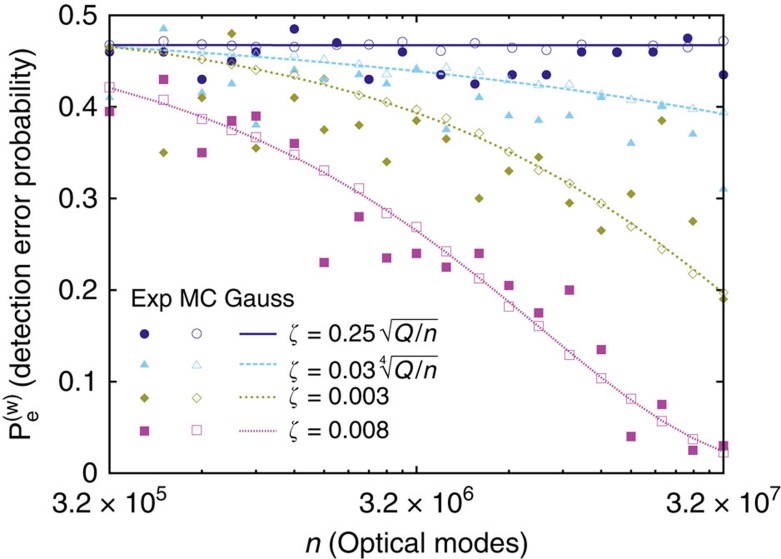
Willie's error probability. Estimates from 100 experiments have solid fill, estimates from 10^5^ Monte-Carlo simulations have clear fill and Gaussian approximations are lines. The 95% confidence intervals (computed in the Methods) for the experimental estimates are ±0.136; for the Monte-Carlo simulations, they are ±0.014. Alice transmits 
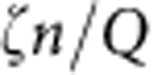
 PPM symbols on average and Willie's error probability remains constant when Alice obeys SRL and uses 
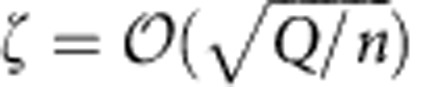
; it drops as *n* increases if Alice breaks SRL by using an asymptotically larger 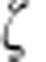.

Willie's detection problem can be reduced to a test between two simple hypotheses where the log-likelihood ratio test minimizes 
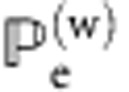
 (ref. 33). Figure 4 illustrates Willie's probability of error estimates from the experiments and the Monte-Carlo study, as well as its analytical Gaussian approximation; implementation details are found in the Methods. Monte-Carlo simulations show that the Gaussian approximation is accurate. More importantly, Fig. 4 highlights Alice's safety when she obeys SRL and her peril when she does not. When 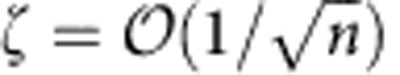, 
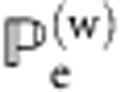
 remains constant as *n* increases. However, for asymptotically larger 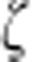, 
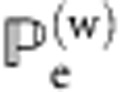
 drops at a rate that depends on Alice's carelessness. The drop at 
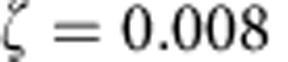
 vividly demonstrates our converse.

**Figure 5:**
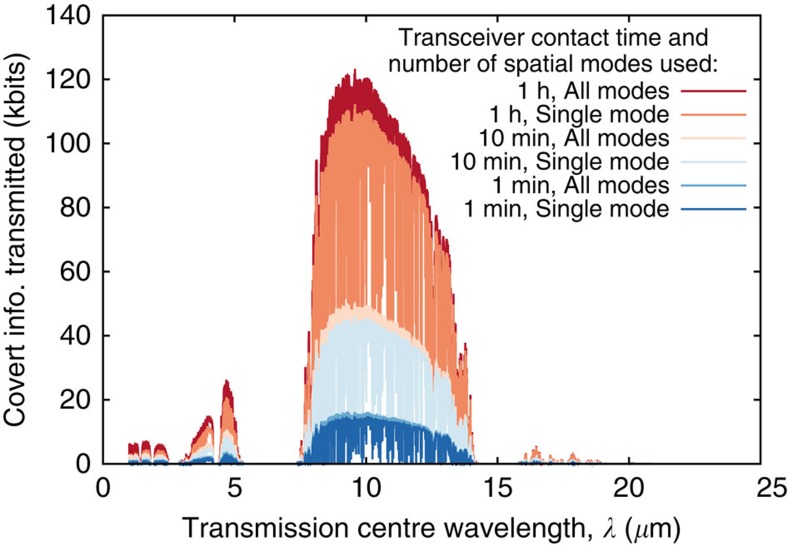
Quantum-secure covert communication performance. Alice and Bob are separated by an *L*=1 km line-of-sight channel and use equal-sized *r*=10 cm aperture radii. We assume a *W*=10 GHz source bandwidth. Although we calculate the number of covertly transmitted bits for centre wavelength 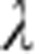 ranging from 1 μm to 20 cm, it is 0 for 
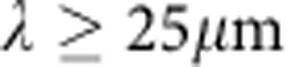
 and thus not plotted. The details of the calculation are in the Methods. We use the MODTRAN ‘Mid-Latitude Summer (MLS)' atmospheric model^34^ at a 10-m elevation from the ground level with 23 km visibility in clear weather propagation for estimating the atmospheric extinction due to scattering and absorption. For thermal background estimation, we use the total radiance at a 60° solar elevation from the same MODTRAN model. The optimal centre wavelength is 9.58 μm, where the use of all available spatial modes results in only 10% increase in covert information transmitted.

Our choice of 1.55 μm for centre wavelength was driven by the availability of a laser source and SPDs at that wavelength in our laboratory. However, the thermal noise power at 1.55 μm is very low. If Alice desires to hide her transmissions against the most powerful adversary permissible by quantum mechanics, she must rely solely on the background radiation at the operating wavelength and not on the non-zero dark click probability of Willie's detector. Thus, longer wavelengths may be more attractive for such quantum-secure covert communication. Consider both Alice and Bob using identical apertures with 10 cm radii and communicating over a 1-km line-of-sight free-space optical channel. Let us make an extremely conservative assumption that all the transmitted photons that do not reach Bob—either because of diffraction-limited loss or the loss from scattering and absorption by atmospheric aerosols—are available to Willie. In Fig. 5 we present the performance of quantum-secure covert communication under these assumptions as a function of the transmitter's centre wavelength and transceiver contact duration. Our calculation spanned the range of wavelengths from 1 μm to 20 cm. We employ a detailed model of the atmosphere^34^ that includes wavelength-dependent loss from absorption and scattering, as well as wavelength-dependent noise from solar and blackbody radiation. We calculate the number of quantum-secure covert bits transmissible using an infinite set of orthogonal spatial modes, as well as under a practical restriction to a single focused Gaussian beam. The details of the calculation are in the Methods.

The results of our analysis illustrated in Fig. 5 support the optimality of LWIR wavelengths 
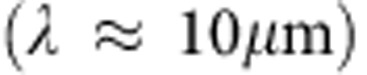
 for quantum-secure covert communication. We note that at those wavelengths, the performance of a single focused Gaussian beam almost matches that of the full orthogonal spatial mode set, thereby showing that employing multiple spatial beams yields only a modest benefit. This arises because quantum-secure covert communication is highly sensitive to the losses of transmitted photons, as those photons are assumed to be captured by Willie. As the higher-order spatial modes have progressively higher diffraction-limited loss (see the Supplementary Note 1), their benefit to covert communication is not significant. Diffraction-limited loss also precludes covert communication at longer wavelengths, even though the amount of background noise is substantially greater. On the other hand, although shorter wavelengths support smaller diffraction-limited loss and a greater number of orthogonal spatial modes with suitable input–output power transfer, the power of background noise is insufficient to hide a significant amount of covert data. That being said, relaxing the assumptions on Willie's capabilities (for example, allowing him to capture only a fraction of transmitted photons that are not received by Bob) will certainly benefit covert communication and may change the optimal centre wavelength. However, we defer this to future work.

## Discussion

We determined that quantum-secure covert communication is achievable over a lossy–noisy bosonic channel, provided that the adversary's measurement is subject to non-adversarial excess noise. Excess noise that is not controlled by the adversary is crucial, as we show that an adversary with full control over all noise sources can prevent covert communication, in contrast to the QKD scenario. Excess noise affecting practical detectors (for example, blackbody radiation and dark counts) allows covert communication, as demonstrated for the first time in our proof-of-concept optical covert communication experiment.

Our contributions motivate exploration of many open questions in quantum-secure covert communication. We stated the problem of generalizing the quantum-secure covert communication results of this paper to arbitrary quantum channels. Furthermore, even for the bosonic channel, exact characterization of the covert communication volume is a significant open problem. The analysis in the remark following the proof of Theorem 2 in the Supplementary Note 3 provides insight into the relationship between the maximum quantum-secure covert communication throughput, the system parameters (reliability and detectability) and channel parameters (noise power level and transmissivity). However, establishing the exact relationship, as was done for classical covert communication^13^,^14^, would yield the equivalent of the channel capacity in standard reliable communication and enable characterization of the structured receivers that attain this maximum.

The success of our proof-of-concept demonstration naturally calls for a full experimental validation of quantum-secure covert communication that only relies on naturally occurring background noise from the blackbody and solar radiance. Optical signalling at shorter wavelengths is usually deemed particularly attractive for covert communication in free space because of its narrow diffraction-limited beam spread in free space^35^,^36^ (which leads to smaller loss compared with longer wavelengths for a given channel geometry). However, we show that the amount of transmissible covert data critically depends on the channel's thermal noise, which is much stronger at longer wavelengths. Therefore, wavelength-dependent atmospheric loss and scattering, as well as wavelength-dependent total thermal radiation afflicting all receivers must be taken into account when analysing covert communication. Our calculations of quantum-secure covert communication throughput indicate that the LWIR regime provides the optimal balance of noise from blackbody radiation and diffraction-limited losses. We also show the feasibility of implementing quantum-secure covert communication using standard laser equipment, and that a single Gaussian focused beam achieves performance close to that of a system employing an infinite set of orthogonal spatial modes. That said, our analysis assumes the most powerful adversary imaginable and the impact of relaxing the assumptions on adversary's capabilities should be investigated.

Finally, our recent results on jammer-assisted covert communication^37^ also motivate the extension of this work to networked settings, where friendly jammers aid stealthy communication by generating uncoordinated random chatter.

## Methods

### Covert communication theorems

Here we state our theorems, with proofs in the Supplementary Note 3. Each theorem can be classified as either an ‘achievability' or a ‘converse'. Achievability theorems (2, 3 and 4) establish the lower limit on the amount of information that can be covertly transmitted from Alice to Bob, whereas the converse theorems (1 and 5) demonstrate the upper limit. In essence, the achievability results are obtained by the following steps: fixing Alice's and Bob's communication system and revealing its construction in entirety (except the shared secret) to Willie; showing that, even with such information, any detector Willie can choose within some natural constraints is ineffective at discriminating Alice's transmission state; and demonstrating that the transmission can be reliably decoded by Bob using the shared secret. On the other hand, converses are established by: fixing Willie's detection scheme (and revealing it to Alice and Bob) and demonstrating that no amount of resources allows Alice to both remain undetected by Willie and exceed the upper limit on the amount of information that is reliably transmitted to Bob. We start by claiming the inability to instantiate covert communication in the absence of excess noise.

*Theorem 1*. Suppose Willie has a pure-loss channel from Alice and is limited only by the laws of physics in his receiver measurement choice. Then Alice cannot communicate to Bob reliably and covertly even if Alice and Bob have access to a pre-shared secret of unbounded size, an unattenuated observation of the transmission and a quantum-optimal receiver.

Next, we claim the achievability of the SRL when Willie's channel is subject to excess noise. We first consider a lossy bosonic channel with additive thermal noise and claim achievability even when Willie has arbitrary resources such as any quantum-limited measurement on the isometric extension of the Alice-to-Bob quantum channel (that is, access to all signalling photons not captured by Bob).

*Theorem 2*. Suppose Willie has access to an arbitrarily complex receiver measurement as permitted by the laws of quantum physics and can capture all the photons transmitted by Alice that do not reach Bob. Let Willie's channel from Alice be subject to noise from a thermal environment that injects 
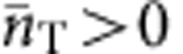
 photons per mode on average and let Alice and Bob share a secret of sufficient length before communicating. Then Alice can lower bound Willie's detection error probability 
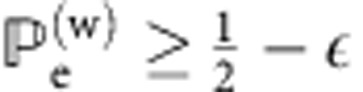
 for any ε>0, while reliably transmitting 
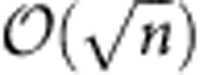
 bits to Bob in *n* modes even if Bob only has access to a (sub-optimal) coherent detection receiver, such as an optical homodyne detector.

In the remaining theorems, Willie's detector is a noisy photon number resolving (PNR) receiver. An ideal PNR receiver is an asymptotically optimal detector for Willie in the pure-loss regime (as discussed in the remark following the proof of Theorem 1 in the Supplementary Note 3). However, any practical implementation of a PNR receiver has a non-zero dark current. It is also worth noting that a PNR receiver can be used to mimic an SPD (but not vice versa). Theorems 3 and 4 show that noise from the resulting dark counts enables covert communication even over a pure-loss channel. We model the dark counts per mode in Willie's PNR detector as a Poisson process with average number of dark counts per mode 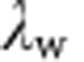.

*Theorem 3*. Suppose that Willie has a pure-loss channel from Alice, captures all photons transmitted by Alice that do not reach Bob, but is limited to a receiver with a non-zero dark current. Let Alice and Bob share a secret of sufficient length before communicating. Then Alice can lower bound Willie's detection error probability 
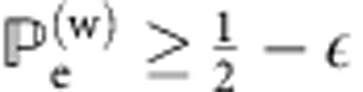
 for any *ε>0*, while reliably transmitting 
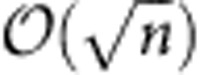
 bits to Bob in *n* modes.

The proof of Theorem 3 demonstrates that 
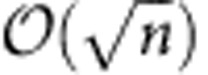
 covert bits can be reliably transmitted using OOK coherent state modulation where Alice transmits the ‘on' symbol 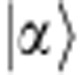 with probability 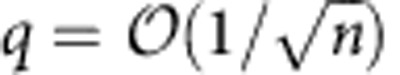 and the ‘off' symbol |0〉 with probability 1−q. However, the skewed on–off duty cycle of OOK modulation makes construction of efficient ECCs challenging. We thus consider PPM, which constrains the OOK signalling scheme, enabling the use of many efficient ECCs by sacrificing a constant fraction of throughput. Each PPM symbol uses a PPM frame to transmit a sequence of Q coherent state pulses, 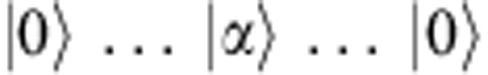, encoding message i∈{1, 2,…, Q} by transmitting 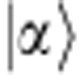 in the ith mode of the PPM frame. Next, we claim that the square-root scaling is achievable under this structural constraint.

*Theorem 4*. Suppose that Willie has a pure-loss channel from Alice, can capture all photons transmitted by Alice that do not reach Bob, but is limited to a PNR receiver with a non-zero dark current. Let Alice and Bob share a secret of sufficient length before communicating. Then Alice can lower bound Willie's detection error probability 
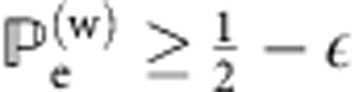
 for any *ε>0*, while reliably transmitting 
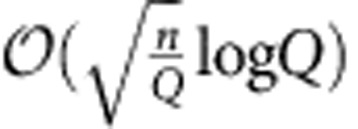
 bits to Bob using *n* modes and a *Q*-ary *PPM* constellation.

Finally, we claim the unsurmountability of the SRL. We assume non-zero thermal noise 
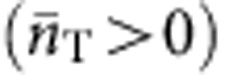
 in the channel and non-zero dark count rate 
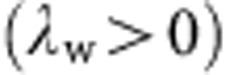
 in Willie's detector. Setting 
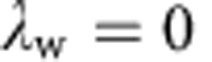
 yields the converse for Theorem 2 and setting 
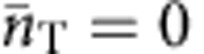
 yields the converse for Theorems 3 and 4. Setting 
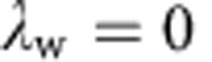
 and 
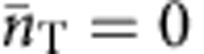
 yields the conditions for Theorem 1. To state the theorem, we use the following asymptotic notation^38^: we say 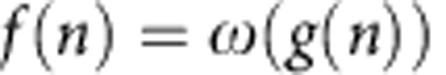 when g(n) is a lower bound that is not asymptotically tight.

*Theorem 5*. Suppose Alice only uses *n*-mode codewords with total photon number variance 
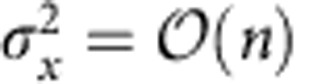
. Then, if she attempts to transmit 
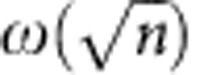
 bits in *n* modes, as *n*→∞, she is either detected by Willie with arbitrarily low detection error probability or Bob cannot decode with arbitrarily low decoding error probability.

The restriction on the photon number variance of Alice's input states is not onerous, as it subsumes all well-known quantum states of a bosonic mode. However, proving this theorem for input states with unbounded photon number variance per mode remains an open problem.

Next, we provide details of the experimental methodology.

### Alice's encoder

Before communication, Alice and Bob secretly and jointly select a random subset 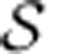 of PPM frames to use for transmission: each of the *n*/*Q* available PPM frames is selected independently with probability 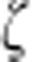. Alice and Bob then secretly generate a vector **k** containing 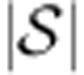 integers selected independently and uniformly at random from {0, 1,…, *Q*-1}, where 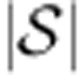 denotes the cardinality of 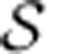. Alice encodes a message into a codeword of size 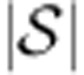 using an RS code. She adds **k** modulo-*Q* to this message and transmits it on the PPM frames in 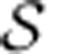. We note that this is almost identical to the construction of the coding scheme in the proof of Theorem 4 (see the Supplementary Note 3), with the exception of the use of an RS code for error correction.

### Generation of transmitted symbols

Alice generates the length-*n* binary sequence describing the transmitted signal, with a ‘1' at a given location indicating a pulse in that mode and a ‘0' indicating the absence of a pulse. First, Alice encodes random data, organized into *Q*-ary symbols, with an RS code and modulo-*Q* addition of **k** to produce a coded sequence of *Q*-ary symbols. The value of the *i*th symbol in this sequence indicates which mode in the *i*th PPM symbol in the set 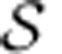 contains a pulse, whereas all modes of the PPM frames not in 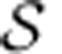 remain empty. Mapping occupied modes to ‘1' and unoccupied modes to ‘0' results in the desired length-*n* binary sequence.

To accurately estimate Willie's detection error probability in the face of optical power fluctuations, the above binary sequence is alternated with a sequence of *n* ‘0's, to produce a final length-2*n* sequence that is passed to the experimental setup. Willie gets a ‘clean' look at the channel when Alice is silent using these interleaved ‘0's, thus allowing the estimation of both the false alarm and the missed detection probabilities under the same conditions. Bob simply discards the interleaved ‘0's.

### Additional implementation details

Geiger-mode photodiodes have to reset after each detection event, resulting in a deterministic number of no-clicks always following a click^39^. This is known as the dead time *t*_d_ of a detector, and, in our experiment, *t*_d_=16 observation periods.

We used the following maximum likelihood estimator of the dark click probability to calculate the estimates of *p*_D_ in Table 1:


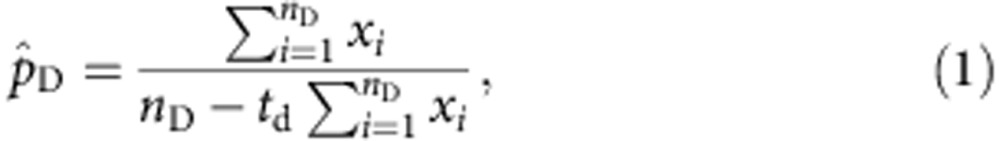


where 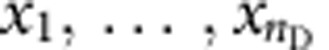 is the sequence of *n*_D_ observations where only the dark clicks can be observed, that is, it is the experimental click record that excludes the observations of Alice's transmissions as well as the dead time following the detected transmissions. We provide extensive analysis of detector dark clicks in the Supplementary Note 7. Although Supplementary Figs 1 and 2 show that, during our experiments, the dark click probability varied with time, Supplementary Fig. 3 demonstrates that the effect of this variation on our analysis is minimal.

### Bob's decoder

Bob examines only the PPM frames in 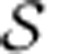. If two or more pulses are detected in a PPM frame, one of them is selected uniformly at random. If no pulses are detected, it is labelled as an erasure. After subtracting **k** modulo-*Q* from this vector of PPM symbols (subtraction is not performed on erasures), the resultant vector is passed to the RS decoder.

For each experiment we record the total number of bits in the successfully decoded codewords; the undecoded codewords are discarded. For each pair of parameters 
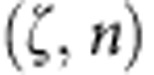
 we report the mean of the total number of decoded bits over 100 experiments. The reported symbol error rate is the total number of lost data symbols during all the experiments at the specified communication regime divided by the total number of data symbols transmitted during these experiments. The calculation of the maximum number of covert bits that can be received by Bob is presented in the Supplementary Note 4.

### Willie's detector

Willie's detection problem can be reduced to a test between two simple hypotheses where the log-likelihood ratio test minimizes 
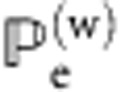
 (ref. 33). The test statistic for the log-likelihood ratio test is derived in the Supplementary Note 5 and is simply the total number of clicks *Y* observed by Willie. Willie compares *Y* with a threshold *S*, accusing Alice if *Y*≥*S*. Willie chooses the value of *S* that minimizes Willie's detection error probability 
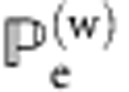
.

For each pair of parameters 
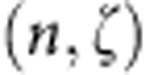
 as well as Alice's transmission state, we perform *m* experiments, recording the observed number of clicks *Y*. We denote by 
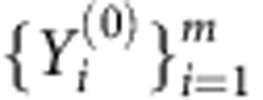
 and 
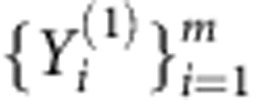
 the sequences of experimentally observed click counts when Alice does not transmit and transmits, respectively. To estimate Willie's detection error probability 
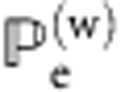
, we construct empirical distribution functions 
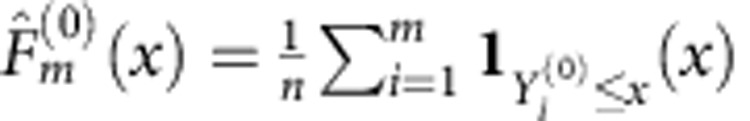
 and 
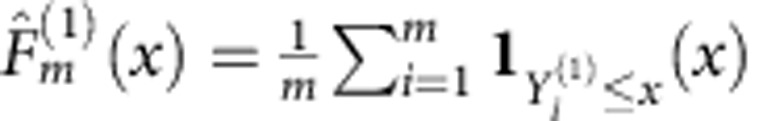
, where 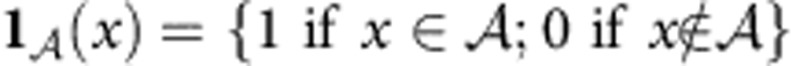 denotes the indicator function. The estimated probability of error is then


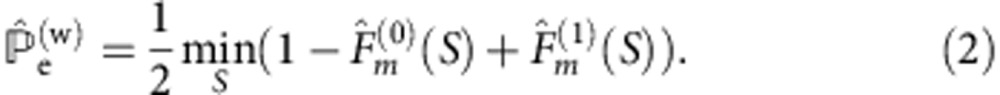


We perform a Monte-Carlo study using 10^5^ simulations per 
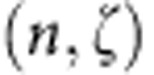
 pair. We generate, encode and detect the messages as in the physical experiment and use equation (2) to estimate Willie's probability of error, but simulate the optical channel induced by our choice of a laser-light transmitter and an SPD using its estimated characteristics reported in Table 1. Similarly, we use the values in Table 1 for our analytical Gaussian approximation of 
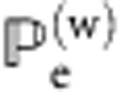
 described in the Supplementary Note 6.

We compute the confidence intervals for the estimate in equation (2) using Dvoretzky–Keifer–Wolfowitz inequality^40^,^41^, which relates the distribution function *F*_*X*_(*x*) of random variable *X* to the empirical distribution function 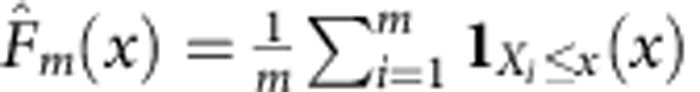 associated with a sequence 
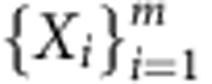
 of *m* i.i.d. draws of the random variable *X* as follows:


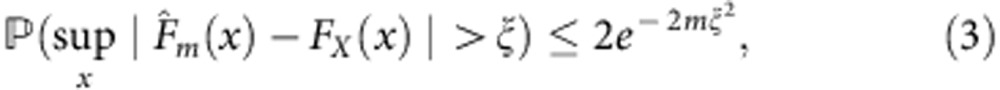


where 
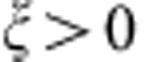
. For *x*_0_, the (1−*α*) confidence interval for the empirical estimate of *F*(*x*_0_) is given by 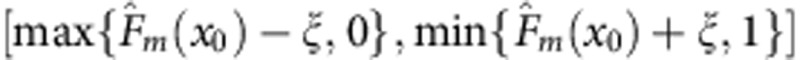 where 
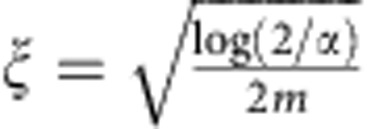
. Thus, 
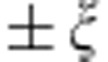
 is used for reporting the confidence intervals in Fig. 4.

### Calculation of the number of quantum-secure covert bits

We employ all the degrees of freedom of the photon, as described in the Supplementary Note 1. We could calculate covert communication volume for any aperture shape. However, we assume that Alice and Bob use soft Gaussian-attenuated apertures in the transmitter and receiver pupil, respectively. This makes the input–output eigenmodes either Laguerre–Gaussian or Hermite–Gaussian spatial modes, whose input–output power transmissivities have simple analytically tractable expressions. Thus, the set of parallel channels available to Alice and Bob is countably infinite and contains *q* independent channels, *q*=1, 2,…, with diffraction-limited transmissivity *η*_*q*_, whose expression is given by equation (4) in the Supplementary Note 1. Transmissivity decays exponentially with increasing *q* and, as we assume that Willie captures all the photons not received by Bob, the potential benefit from using modes with higher indices (and low transmissivity) may be outweighed by the increased risk of detection.

In the proof of Theorem 2 in the Supplementary Note 3, Alice ensures ineffectiveness of Willie's detector by upper bounding the quantum relative entropy (QRE) between states 
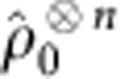
 and 
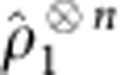
 corresponding to Willie's observations when Alice is quiet and transmitting, respectively. Effectively, equations (42), (43) and (45) in the Supplementary Note 3 establish a quadratic constraint on Alice's mean photon number per mode 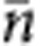. The proof of Theorem 2 assumes *n* orthogonal modes corresponding to *n* identical parallel channels each with transmissivity *η*. The additivity of QRE allows a trivial extension to orthogonal modes corresponding to parallel channels with different transmissivities. However, finding the maximum number of reliably transmissible covert bits requires solving the following constrained optimization problem:


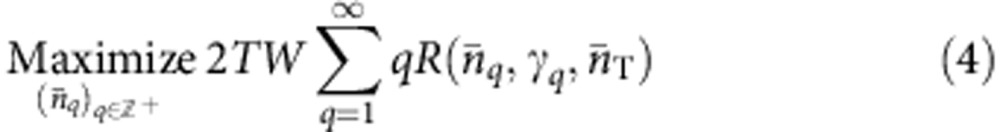



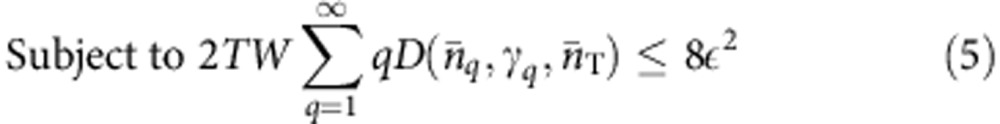



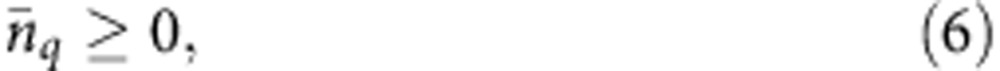


where 
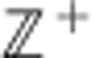
 denotes the set of positive integers, 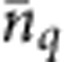 is the mean photon number per mode transmitted on all *q* channels with transmissivity 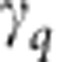, 
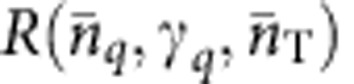
 denotes transmitted bits per mode and 
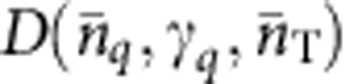
 is the per-mode contribution to the total QRE by the transmission. Transmissivity is the product 
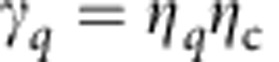
, where *η*_*q*_ is the diffraction-limited transmissivity given by equation (4) in the Supplementary Note 1 and *η*_c_ accounts for the loss from scattering and absorption by the environment provided by the MODTRAN model. The thermal noise mean photon number per mode 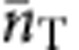 is calculated using equation (40) in ref. 42, from the total solar and blackbody radiance provided by the MODTRAN model. Thus, *η*_*q*_, *η*_c_ and 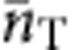 depend on the transmission centre wavelength 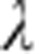. It is worth noting that solving the optimization problem is not necessary to calculate the covert volume using a single Gaussian beam, as this only employs a single channel (*q*=1). The remaining details, including the solution to the optimization problem in equations (4) to (6), are in the Supplementary Note 8.

## Additional information

**How to cite this article:** Bash, B. A. *et al.* Quantum-secure covert communication on bosonic channels. *Nat. Commun.* 6:8626 doi: 10.1038/ncomms9626 (2015).

## Supplementary Material

Supplementary Information Supplementary Figures 1-3, Supplementary Notes 1-8 and Supplementary References

## Figures and Tables

**Table 1:** Optical channel characteristics.

**Experimental estimates**	**Willie**		**Bob**	
	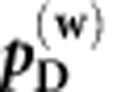	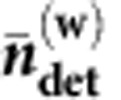	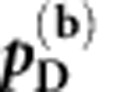	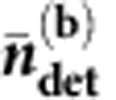
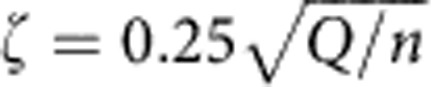	9.15 × 10^−5^	0.036	2.99 × 10^−6^	1.52
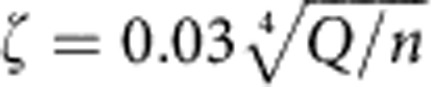	9.11 × 10^−5^	0.032	2.55 × 10^−6^	1.14
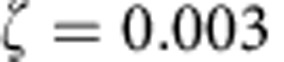	9.29 × 10^−5^	0.032	2.65 × 10^−6^	1.07
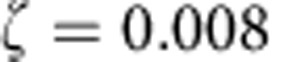	9.27 × 10^−5^	0.028	2.68 × 10^−6^	1.05
Target:	9 × 10^−5^	0.03	3 × 10^−6^	1.4

## References

[b1] Menezes A. J., Vanstone S. A. & Oorschot P. C. V. Handbook of Applied Cryptography 1st edn CRC Press, Inc. (1996).

[b2] Talbot J. & Welsh D. Complexity and Cryptography: An Introduction Cambridge University Press (2006).

[b3] Wyner A. D. The wiretap channel. Bell Syst. Tech. J. 54, 1355 (1975).

[b4] Csiszár I. & Körner J. Broadcast channels with confidential messages. IEEE T Inform Theory 24, 339–348 (1978).

[b5] Bloch M. & Barros J. Physical-Layer Security Cambridge Univ. Press (2011).

[b6] Bennett C. H. & Brassard G. in Proceedings of IEEE International Conference On Computers, Systems, and Signal Processing 175–179Bangalore, India (1984).

[b7] Shannon C. E. Communication theory of security. Bell Syst. Tech. J. 28, 656–715 (1949).

[b8] Simon M. K., Omura J. K., Scholtz R. A. & Levitt B. K. Spread Spectrum Communications Handbook revised edn McGraw-Hill (1994).

[b9] Fridrich J. Steganography in Digital Media: Principles, Algorithms, and Applications 1st edn Cambridge Univ. Press (2009).

[b10] Shannon C. E. A mathematical theory of communication. Bell Syst. Tech. J. 27, 379–423, 623–656 (1948).

[b11] Bash B. A., Goeckel D. & Towsley D. Limits of reliable communication with low probability of detection in AWGN channels. IEEE J. Sel. Areas Commun. 31, 1921–1930 (2013).

[b12] Bash B. A., Goeckel D. & Towsley D. in Proceedings of IEEE International Symposium on Information Theory (ISIT) 448–452Cambridge, MA, USA (2012).

[b13] Bloch M. in Proceedings of IEEE International Symposium on Information Theory (ISIT) Hong Kong, China (2015).

[b14] Wang L., Wornell G. W. & Zhang L. in Proceedings of IEEE International Symposium on Information Theory (ISIT) Hong Kong, China (2015).

[b15] Bash B. A., Goeckel D. & Towsley D. in Proceedings of IEEE International Symposium on Information Theory (ISIT) 606–610Honolulu, HI, USA (2014).

[b16] Che P. H., Bakshi M. & Jaggi S. in Proceedings of IEEE International Symposium on Information Theory (ISIT) 2945–2949Istanbul, Turkey (2013).

[b17] Kadhe S., Jaggi S., Bakshi M. & Sprintson A. in Proceedings of IEEE International Symposium on Information Theory (ISIT) 611–615Honolulu, HI, USA (2014).

[b18] Hou J. & Kramer G. in Proceedings of IEEE International Symposium on Information Theory (ISIT) 601–605Honolulu, HI, USA (2014).

[b19] Ker A. D. Lecture Notes in Computer Science vol. 4437, 265–281Springer (2007).

[b20] Filler T., Ker A. D. & Fridrich J. in Proceedings of SPIE 7254, Media Forensics and Security (eds Delp E. J., Dittmann J., Memon N. D., Wong P. W. 725408 (2009).

[b21] Ker A. D. in Proceedings of the 11th ACM Workshop on Multimedia and Security 85–92Princeton, NJ, USA (2009).

[b22] Ker A. D. in Proceedings of the 12th ACM Workshop on Multimedia and Security 213–224Rome, Italy (2010).

[b23] Shaw B. A. & Brun T. A. Quantum steganography with noisy quantum channels. Phys. Rev. A 83, 022310 (2011).

[b24] Craver S. & Yu J. in Proceedings of SPIE 7541, Media Forensics and Security II (eds Memon M. D., Dittmann J., Alattar A. M., Delp III E. J. 754103 (2010).

[b25] Giovannetti V. *et al.* Classical capacity of the lossy bosonic channel: the exact solution. Phys. Rev. Lett. 92, 027902 (2004). 14753969 10.1103/PhysRevLett.92.027902

[b26] Wolf M. M., Pérez-García D. & Giedke G. Quantum capacities of bosonic channels. Phys. Rev. Lett. 98, 130501 (2007). 17501173 10.1103/PhysRevLett.98.130501

[b27] Wilde M. M., Hayden P. & Guha S. Information trade-offs for optical quantum communication. Phys. Rev. Lett. 108, 140501 (2012). 22540777 10.1103/PhysRevLett.108.140501

[b28] Abdo B. *et al.* Josephson directional amplifier for quantum measurement of superconducting circuits. Phys. Rev. Lett. 112, 167701 (2014). 24815669 10.1103/PhysRevLett.112.167701

[b29] Holevo A. S. The capacity of the quantum channel with general signal states. IEEE Trans. Inf. Theory 44, 269–273 (1998).

[b30] Wang L. & Wornell G. W. in Proceedings in IEEE Information Theory Workshop (ITW) 582–586Lausanne, Switzerland (2012).

[b31] Moision B., Hamkins J. & Cheng M. in Information Theory and its Applications (ITA) Workshop University of California–San Diego (2006).

[b32] Makarov V. Controlling passively quenched single photon detectors by bright light. N. J. Phys. 11, 065003 (2009).

[b33] Lehmann E. & Romano J. Testing Statistical Hypotheses 3rd edn Springer (2005).

[b34] Berk A. *et al.* in Proceedings of SPIE 6233, Algorithms and Technologies for Multispectral, Hyperspectral, and Ultraspectral Imagery XII (eds Shen S. S., Lews P. E 62331f (2006).

[b35] Gagliardi R. & Karp S. Optical Communications 2nd edn Wiley (1995).

[b36] Goodman J. Introduction to Fourier Optics 3rd edn Roberts & Company (2005).

[b37] Soltani R., Bash B. A., Goeckel D., Guha S. & Towsley D. in Conference on Communication, Control, Computing (Allerton) 1078–1085Monticello, IL, USA (2014).

[b38] Cormen T. H., Leiserson C. E., Rivest R. L. & Stein C. Introduction to Algorithms 2nd edn MIT Press (2001).

[b39] Itzler M., Entwistle M. & Jiang X. in Proceedings of IEEE Photonics Conference (PHO) 348–349Arlington, VA, USA (2011).

[b40] Dvoretzky A., Kiefer J. & Wolfowitz J. Asymptotic minimax character of the sample distribution function and of the classical multinomial estimator. Ann. Math. Stat. 27, 642–669 (1956).

[b41] Massart P. The tight constant in the Dvoretzky-Kiefer-Wolfowitz inequality. Ann. Probab. 18, 1269–1283 (1990).

[b42] Shapiro J. H., Guha S. & Erkmen B. I. Ultimate channel capacity of free-space optical communications. J. Opt. Network. 4, 501–516 (2005).

