# Supplementary material for: Quantum-secure covert communication on bosonic channels
Source: Nat Commun. 2015 Oct 19;6:8626. doi: 10.1038/ncomms9626 (PMC4667704; doi:10.1038/ncomms9626)
Supplement: Supplementary Information — Supplementary Figures 1-3, Supplementary Notes 1-8 and Supplementary References [file ncomms9626-s1.pdf]

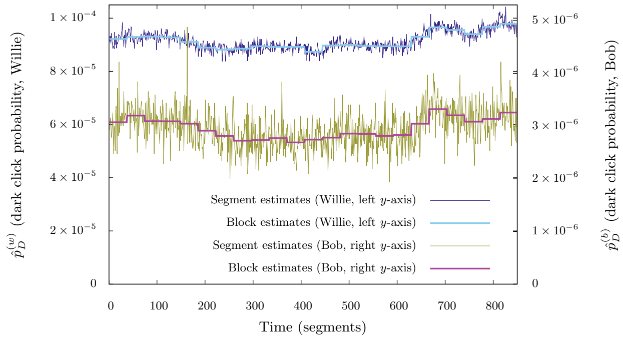

(a) $\zeta = 0.25/\sqrt{n}$

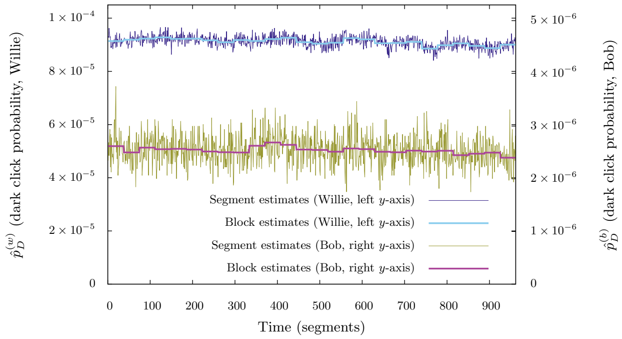

(b) $\zeta = 0.03/\sqrt[4]{n}$

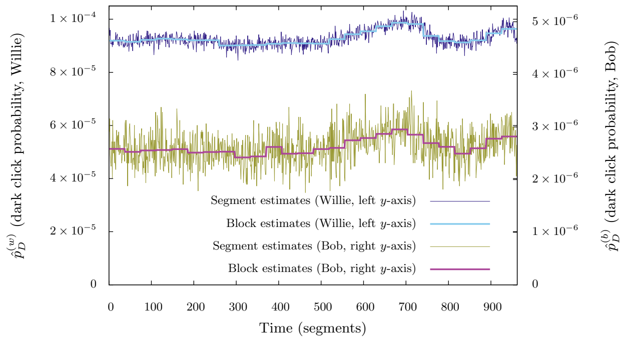

(c) $\zeta = 0.003$

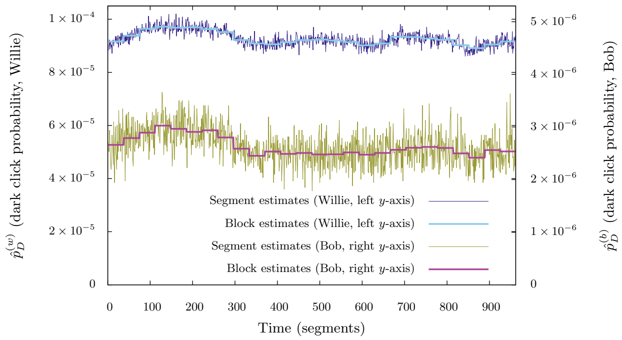

(d) $\zeta = 0.008$

Supplementary Figure 1. Temporal variation in dark click probability. The estimates of Willie's dark click probability are plotted using the left y-axis; the estimates of Bob's dark click probability are plotted using the right y-axis. Dark click probability is estimated using equation (81) for consecutive segments, each containing $n_{\mathrm{D,s}} = 3.2\times10^7$ consecutive observations, as well as for consecutive blocks, each containing $n_{\mathrm{D,b}} = 1.184 \times 10^9$ consecutive observations (with the exception of the last block for $\zeta = 0.25/\sqrt{n}$ in panel ((a)) where $n_{\mathrm{D,b}} = 1.152 \times 10^9$ observations).

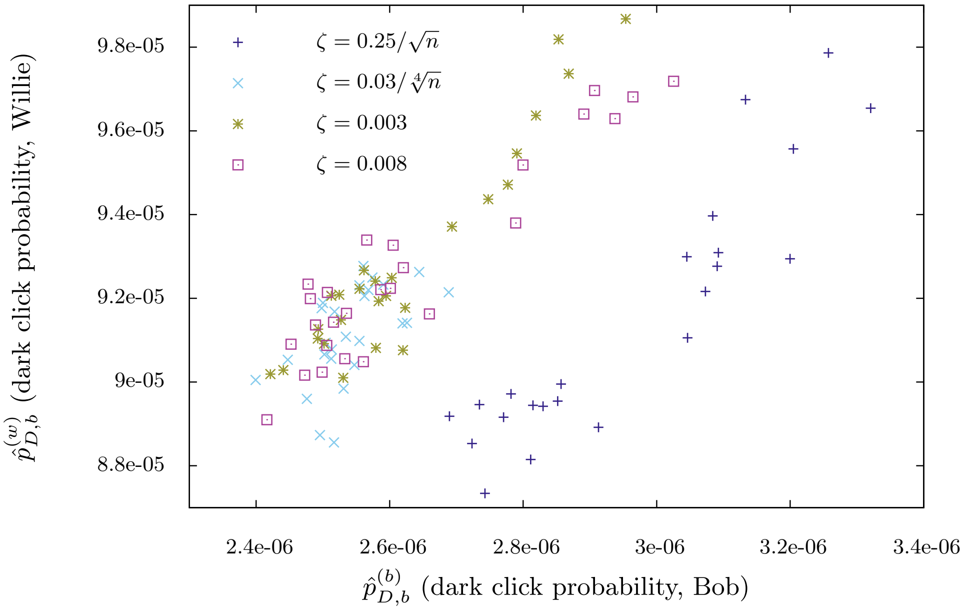

Supplementary Figure 2. Correlation in Willie's and Bob's dark click probabilities. A glitch in the PCIe-6537 data acquisition card resulted in a slight change in setup between the experiments corresponding to $\zeta = 0.25/\sqrt{n}$ and $\zeta \in \{0.03/\sqrt[4]{n}, 0.003, 0.008\}$.

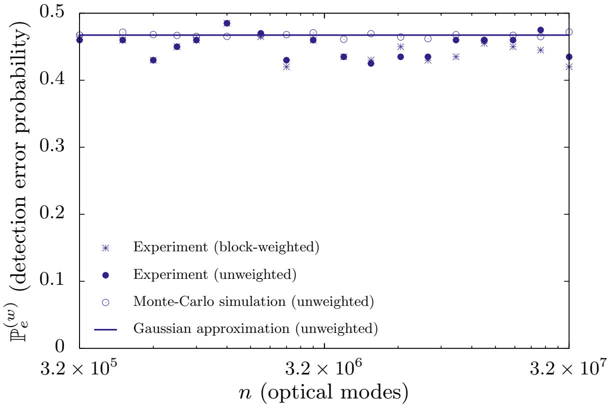

(a) $\zeta = 0.25/\sqrt{n}$

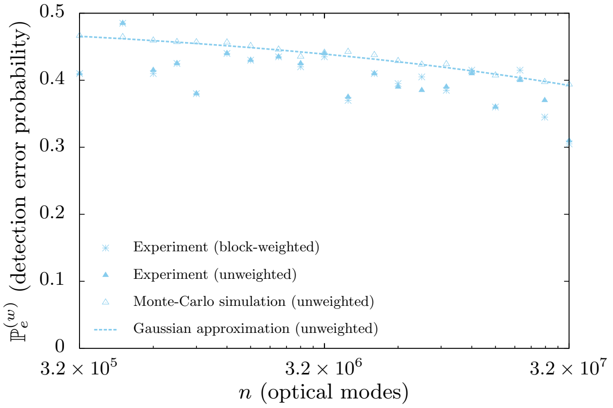

(b) $\zeta = 0.03/\sqrt[4]{n}$

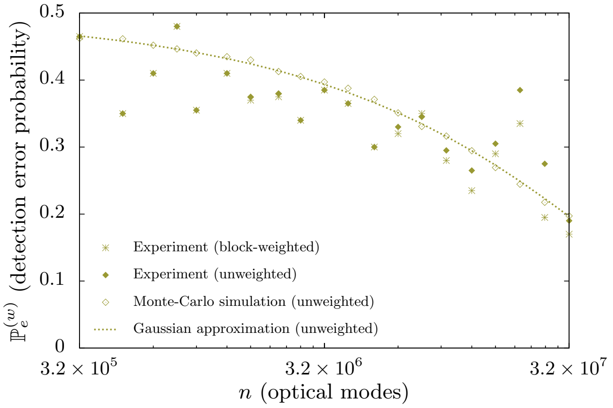

(c) $\zeta = 0.003$

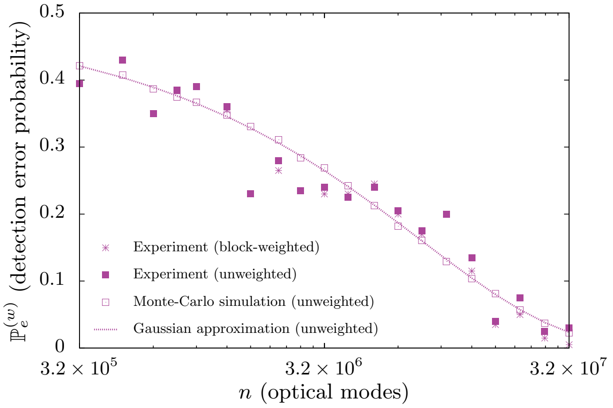

(d) $\zeta = 0.003$

Supplementary Figure 3. Impact of variations in dark click probability on the estimates of Willie's detection error. The probability of detection error estimated from the block-weighted test statistic given in equation (82) is plotted using the asterisks alongside the plots from Figure 4 of the estimates using the (unweighted) total click count. Weighting does not substantially change the detection error probability estimates.

Supplementary Note 1. Bosonic Modes and the Degrees of Freedom of the Photon

Let us consider propagation of linearly-polarized, quasi-monochromatic light with center wavelength λ (that is, a narrow transmission band $\Delta\lambda \ll \lambda$ around the center wavelength) from Alice's transmitter pupil in the $z = 0$ transverse plane with aperture function $A_{\rm T}(\mathbf{r})$, $\mathbf{r} \equiv (x, y)$, through a L-meter line-of-sight free-space channel, and received by Bob's receiver pupil in the $z = L$ plane with aperture function $A_{\rm R}(\mathbf{r}')$, $\mathbf{r}' \equiv (x', y')$. Consider Alice's transmitted field's complex envelope $E_0(\mathbf{r}, t)$. It is multiplied (truncated) by the complex-valued transmit-aperture function $A_{\rm T}(\mathbf{r})$, undergoes free-space diffraction over the L-meter path, and is truncated by Bob's receiver-aperture function $A_{\rm R}(\mathbf{r}')$, to yield the received field $E_L(\mathbf{r}', t)$. The overall input-output relationship is described by the following linear-system equation:

$$E_L(\mathbf{r}', t) = \int E_0(\mathbf{r}, t - L/c)\, h(\mathbf{r}', \mathbf{r}, t)\, \mathrm{d}^2\mathbf{r}, \tag{1}$$

where the channel's Green's function $h(\mathbf{r}', \mathbf{r}, t)$ is a spatial impulse response. We assume that the typical time scales of multipath spread (caused by atmospheric turbulence, for instance) is much smaller compared to the communication bandwidth. Therefore, the temporal behavior in the above input-output relationship is limited to a simple L/c delay (c being the speed of light), and hence we drop the time argument from the Green's function. For vacuum propagation (that is, no atmospheric turbulence),

$$h(\mathbf{r}', \mathbf{r}) \equiv h_0(\mathbf{r}', \mathbf{r}) = A_{\rm R}(\mathbf{r}')\, \frac{\exp\left[ik\left(L + |\mathbf{r}' - \mathbf{r}|^2/2L\right)\right]}{i\lambda L}\, A_{\rm T}(\mathbf{r}), \tag{2}$$

where $k = 2\pi/\lambda$. Normal-mode decomposition of the vacuum-propagation Green's function yields an infinite set of orthogonal input-output spatial-mode pairs (a mode being a normalized spatio-temporal field function of a given polarization), that is, an infinite set of non-interfering parallel spatial channels. In other words,

$$\int h_0(\mathbf{r}', \mathbf{r})\Phi_m(\mathbf{r})\mathrm{d}^2\mathbf{r} = \sqrt{\eta_m}\, \phi_m(\mathbf{r}'),\ \text{for}\, m = 1, 2, \ldots, \tag{3}$$

where $\{\Phi_m(\mathbf{r})\}$ forms a complete orthonormal (CON) spatial basis in the transmit-aperture plane before the aperture mask $A_{\rm T}(\mathbf{r})$, and $\{\phi_m(\mathbf{r}')\}$ forms a CON spatial basis in the receiver-aperture plane after the aperture mask $A_{\rm R}(\mathbf{r}')$. The physical meaning is that if Alice excites the spatial mode $\Phi_m(\mathbf{r})$, it in turn excites the corresponding spatial mode $\phi_m(\mathbf{r}')$ (and no other) within Bob's receiver. This specific set of transmitter-plane receiver-plane spatial-mode pairs that form a set of non-interfering parallel channels are the eigenmodes for the channel geometry. The fraction of power Alice puts in the mode $\Phi_m(\mathbf{r})$ that appears in Bob's spatial mode $\phi_m(\mathbf{r}')$ is the modal transmissivity, η_m. We assume that the modes are ordered such that $1 \geq \eta_1 \geq \eta_2 \geq \ldots \eta_m \geq \ldots \geq 0$. If Alice excites the mode $\Phi_m(\mathbf{r})$ in a coherent-state $|\beta\rangle$—the quantum description of an ideal laser-light pulse of intensity $|\beta|^2$ (photons) and phase $\mathrm{Arg}(\beta)$, then the resulting state of Bob's mode $\phi_m(\mathbf{r}')$ is an attenuated coherent state $|\sqrt{\eta_m}e^{i\theta_m}\beta\rangle$, where θ_m is a phase. The power transmissivities $\eta_m(\omega)$ are strictly increasing functions of the transmission frequency $\omega = 2\pi c/\lambda$, each increasing from $\eta_m = 0$ at $\omega = 0$, to $\eta_m = 1$ at $\omega = \infty$. For hard circular pupils of areas $A_{\rm t}$ and $A_{\rm r}$, that is, $A_{\rm T}(\mathbf{r}) = 1, |\mathbf{r}| \leq r_{\rm t}$, $A_{\rm t} \equiv \int |A_{\rm T}(\mathbf{r})|^2\mathrm{d}^2\mathbf{r} = \pi r_{\rm t}^2$, and $A_{\rm R}(\mathbf{r}') = 1, |\mathbf{r}'| \leq r_{\rm r}$, $A_{\rm r} \equiv \int |A_{\rm R}(\mathbf{r}')|^2\mathrm{d}^2\mathbf{r}' = \pi r_{\rm r}^2$, the eigenmodes are prolate spheroidal functions, and the power-transfer eigenvalues $\eta_m(\omega)$, indexed by two integers $m \equiv (m_1, m_2)$, have known, yet quite complicated expressions [1, 2]. For Gaussian-attenuation (soft) aperture pupils, that is, $A_{\rm T}(\mathbf{r}) = \exp\left[-|\mathbf{r}|^2/r_{\rm t}^2\right]$ and $A_{\rm R}(\mathbf{r}') = \exp\left[-|\mathbf{r}'|^2/r_{\rm r}^2\right]$, there are two unitarily-equivalent set of eigenmodes—the Laguerre-Gauss (LG) modes, which have circular symmetry in the transverse plane and are known to carry orbital angular momentum (OAM), and the Hermite-Gauss (HG) modes, which have rectangular symmetry in the transverse plane and do not carry OAM. The power-transfer eigenvalues η_m for either mode set admits the following simple form:

$$\eta_q = \left(\frac{1 + 2D_{\rm f} - \sqrt{1 + 4D_{\rm f}}}{2D_{\rm f}}\right)^q,\ \text{for}\, q = 1, 2, \ldots, \tag{4}$$

where there are q spatial modes of transmissivity η_q. $D_{\rm f} = A_{\rm t}A_{\rm r}/(\lambda L)^2$ is the free-space Fresnel number product for the soft-aperture vacuum-propagation channel, where $A_{\rm t} \equiv \int |A_{\rm T}(\mathbf{r})|^2\mathrm{d}^2\mathbf{r} = \frac{\pi r_{\rm t}^2}{2}$ and $A_{\rm r} \equiv$

$\int |A_{\mathrm{R}}(\mathbf{r}')|^2 \mathrm{d}^2\mathbf{r}' = \frac{\pi r_{\mathrm{r}}^2}{2}$ are the transmitter and receiver aperture's 'areas'. The doubly-indexed HG modes $\Phi_{m,n}(x,y)$ with $n+m+1=q$, $n=0,1,\ldots$, $m=0,1,\ldots$ span the same eigenspace as the doubly-indexed LG modes $\Phi_{p,l}(r,\theta)$ with $2p+|l|+1=q$, $p=0,1,2,\ldots,\ l=0,\pm1,\pm2,\ldots$, and hence are related by a unitary transformation (a linear mode transformation).

Even though these spatial modes form an infinite set of parallel channels, not all of them can be gainfully employed for communication since the power transfer eigenvalues η_m of the higher-order modes diminish rapidly with increasing m. A rough characterization of the number of useable spatial modes is given in terms of the Fresnel number product D_{f}, which is defined as $A_{\mathrm{t}}A_{\mathrm{r}}/(\lambda L)^2$ for a channel with hard-circular pupils. When $D_{\mathrm{f}} \ll 1$, the channel is in the far-field regime, and only one spatial mode pair has an appreciable transmissivity (that is, $n_{\mathrm{S}} = 1$), whose transmissivity $\eta_1 \approx D_{\mathrm{f}}$. On the other hand, when $D_{\mathrm{f}} \gg 1$, the channel is in the near-field regime, and there are $n_{\mathrm{S}} \approx D_{\mathrm{f}}$ mutually-orthogonal spatial-mode pairs, each with $\eta_m \approx 1$. In the far-field propagation regime, using multiple orthogonal spatial modes does not benefit communications. When in the near-field propagation regime however, multiple orthogonal spatial modes can be employed to obtain the ultimate channel capacity associated with a given channel geometry, which exceeds the capacity attained by employing just one spatial mode [3]. Thus generating and separating orthogonal modes have attracted considerable recent attention. Although there has been promising recent work on separating (sorting) mutually-orthogonal LG modes [4], realizing structured physical hardware to sort all LG (or HG) modes efficiently remains a challenging goal. On a positive note, it has been established recently that almost all the aforesaid benefit of using multiple orthogonal spatial modes in the near-field regime can be obtained by simply using multiple focused beams that are spatially separated in the receiver aperture's plane, but not truly mutually orthogonal [5, 6].

In this paper, we evaluate the maximum quantum-secure covert volume attainable by using multiple spatial modes over a 1-km-range free-space channel, and conclude that it peaks in the long-wave infrared regime ($\lambda \approx 10$ μm). Despite the fact that the channel is in the near-field regime at that frequency, multiple orthogonal modes gives only a small advantage over using a single focused Gaussian beam (the $q = 1$ special case of the LG or HG mode sets). This is because covert communication is highly sensitive to lower transmissivities, since we assume that all the uncollected transmitted photons (that is, $1 - \eta_m$ fraction of the transmitted power) is given to the adversary Willie. In our calculations, we do not consider atmospheric turbulence, but do take into account photon loss from atmospheric extinction (in addition to the pure diffraction-limited loss). Atmospheric extinction includes scattering and absorption of photons via atmospheric aerosols, which turns the channel's impulse response to $h(\mathbf{r}',\mathbf{r}) = h_0(\mathbf{r}',\mathbf{r})e^{-\alpha(\lambda)L}$, where $\alpha(\lambda)$ is a wavelength-dependent extinction coefficient. The resulting modal transmissivities are given by $\eta_m e^{-\alpha(\lambda)L}$. See Figure 5 for the plot of the free-space quantum-secure covert volume as a function of the transmission wavelength.

Suppose the transmitter employs n_{S} orthogonal spatial modes with transmissivities $\eta_1, \eta_2, \ldots, \eta_{n_{\mathrm{S}}}$. If W (in Hz) is the total spectral bandwidth that can be supported by the transmitter and the receiver over a transmission window of T seconds, $n_{\mathrm{T}} \approx WT$ mutually-orthogonal temporal modes that can be transmitted [1, 2]. This T-second burst of communication can thus transmit $n = n_{\mathrm{S}} \times n_{\mathrm{T}}$ mutually-orthogonal spatio-temporal modes. Furthermore, if both orthogonal polarizations are employed, the total number of modes becomes $n = 2 \times n_{\mathrm{S}} \times n_{\mathrm{T}}$.

A single-mode fiber of L meter length can support a single spatial mode ($n_{\mathrm{S}} = 1$) of power transmissivity $\eta = e^{-\alpha L}$, where α denotes the fiber's loss coefficient. In this case, $n = 2n_{\mathrm{T}}$. However, the loss coefficient corresponding to the two orthogonal polarizations can be different.

For a detailed development of the quantum theory of free-space diffraction and mode decompositions, the reader is encouraged to refer to Refs. [7–9].

Supplementary Note 2. Discussion of Willie's Detection Error Probability $\mathbb{P}_{\mathrm{e}}^{(\mathrm{w})}$

Willie's detector performs a binary hypothesis test of Alice's transmission state given his observations of the channel. Let's denote by $\mathbb{P}_{\mathrm{FA}}$ the probability that Willie raises a false alarm when Alice does not transmit, and by $\mathbb{P}_{\mathrm{MD}}$ the probability that Willie misses the detection of Alice's transmission. If we assume equal prior probabilities on Alice's transmission state (we relax this assumption later), Willie's detection

error probability, $\mathbb{P}_{\rm e}^{(\rm w)} = (\mathbb{P}_{\rm FA} + \mathbb{P}_{\rm MD})/2$ Since $\mathbb{P}_{\rm e}^{(\rm w)} = 1/2$ for a detector that randomly guesses Alice's transmission state, $\mathbb{P}_{\rm e}^{(\rm w)} \leq 1/2$. Alice's signaling scheme is covert if $\mathbb{P}_{\rm e}^{(\rm w)} \geq 1/2 - \epsilon$ for an arbitrarily small $\epsilon > 0$ regardless of Willie's quantum measurement choice. This has a natural signal processing interpretation via the receiver operating characteristic (ROC) curve [10, Ch. 2.2.2], which plots the probability of true detection $1 - \mathbb{P}_{\rm MD}$ versus the probability of false detection $\mathbb{P}_{\rm FA}$. Since $\mathbb{P}_{\rm FA} \leq 1 - \mathbb{P}_{\rm MD}$ and $\mathbb{P}_{\rm e}^{(\rm w)} \geq \frac{1}{2} - \epsilon$ imply that $\mathbb{P}_{\rm FA} \leq 1 - \mathbb{P}_{\rm MD} \leq \mathbb{P}_{\rm FA} + 2\epsilon$, when ϵ is small, the ROC curve lies very close to the line of no discrimination (the diagonal line where $1 - \mathbb{P}_{\rm MD} = \mathbb{P}_{\rm FA}$). Since the line of no discrimination corresponds to a detector that guesses Alice's transmission state randomly, a small ϵ implies that any detector available to Willie at best does slightly better than a random guess.

Furthermore, we argue that the assumption of a non-trivial prior distribution on Alice's transmission state does not impact our asymptotic results. First, the trace distance between two quantum states is defined as follows:

Definition 1 (Trace distance [11])**.** *The trace distance between two density operators $\hat{\sigma}$ and $\hat{\rho}$ is*

$$\|\hat{\sigma} - \hat{\rho}\|_1 = \mathrm{Tr}\{\sqrt{(\hat{\sigma} - \hat{\rho})^\dagger (\hat{\sigma} - \hat{\rho})}\}. \tag{5}$$

Trace distance relates to the probability of successful discrimination between two quantum states via the following lemma:

Lemma 1. *One half of the trace distance $\frac{1}{2}\left\|\hat{\rho} - \hat{\sigma}\right\|_1$ between quantum states $\hat{\rho}$ and $\hat{\sigma}$ is equal to the largest probability difference that two states $\hat{\rho}$ and $\hat{\sigma}$ could give to the outcome of the same measurement given by the positive semi-definite operator $\hat{\Lambda}$ with eigenvalues upper-bounded by one:*

$$\frac{1}{2}\left\|\hat{\rho} - \hat{\sigma}\right\|_1 = \max_{0 \leq \hat{\Lambda} \leq \hat{I}} \mathrm{Tr}\left\{\Lambda\left(\hat{\rho} - \hat{\sigma}\right)\right\}, \tag{6}$$

where $\hat{I}$ denotes the identity operator.

Proof. See [11, Lemma 9.1.7]. □

Denote by $\hat{\rho}_0$ and $\hat{\rho}_1$ the respective quantum states that Willie observes on his channel from Alice when she does not transmit and transmits. Willie constructs a binary POVM $\{\hat{\Lambda}_0, \hat{\Lambda}_1\}$ to discriminate between these states. Suppose that Willie knows that Alice will not transmit (that is, H_0 is true) with probability π_0 and that she will transmit (that is, H_1 is true) with probability π_1. Thus, π_0 and π_1 denote the prior probabilities of states $\hat{\rho}_0$ and $\hat{\rho}_1$, respectively, where $\pi_0 + \pi_1 = 1$ and the probability of error in discriminating between $\hat{\rho}_0$ and $\hat{\rho}_1$ is

$$\mathbb{P}_{\rm e}^{(\rm w)} = \pi_0 \,\mathrm{Tr}\{\hat{\Lambda}_1 \hat{\rho}_0\} + \pi_1 \,\mathrm{Tr}\{\hat{\Lambda}_0 \hat{\rho}_1\}. \tag{7}$$

The following lemma generalizes the result for $\pi_0 = \pi_1 = \frac{1}{2}$ given in [11, Section 9.1.4] to $\pi_0 \neq \pi_1$:

Lemma 2.

$$\mathbb{P}_{\rm e}^{(\rm w)} \geq \min(\pi_0, \pi_1) - \frac{\max(\pi_0, \pi_1)}{2}\|\hat{\rho}_0 - \hat{\rho}_1\|_1. \tag{8}$$

Proof. First, suppose that $\pi_0 \leq \pi_1$. Since $\{\hat{\Lambda}_0, \hat{\Lambda}_1\}$ is a POVM, $\hat{\Lambda}_0 + \hat{\Lambda}_1 = \hat{I}$. Substituting $\hat{\Lambda}_1 = \hat{I} - \hat{\Lambda}_0$ in (7) and re-arranging the terms, we obtain

$$\begin{aligned} \mathbb{P}_{\rm e}^{(\rm w)} &= \pi_0 \,\mathrm{Tr}\{\hat{\rho}_0\} - \pi_0 \,\mathrm{Tr}\{\hat{\Lambda}_0 \hat{\rho}_0\} + \pi_1 \,\mathrm{Tr}\{\hat{\Lambda}_0 \hat{\rho}_1\} & (9) \\ &= \pi_0 - \mathrm{Tr}\{\hat{\Lambda}_0(\pi_0 \hat{\rho}_0 - \pi_1 \hat{\rho}_1)\}, & (10) \end{aligned}$$

where (10) is because the eigenvalues of a density operator sum to one. When the prior probabilities of the hypotheses are equal: $\pi_0 = \pi_1 = \frac{1}{2}$, an application of Lemma 1 yields the lower bound $\mathbb{P}_{\mathrm{e}}^{(\mathrm{w})} \geq \frac{1}{2} - \frac{1}{4}\|\hat{\rho}_0 - \hat{\rho}_1\|_1$. Now,

$$\mathbb{P}_{\mathrm{e}}^{(\mathrm{w})} = \pi_0 - \mathrm{Tr}\{\hat{\Lambda}_0(\pi_0\hat{\rho}_0 - \pi_1\hat{\rho}_1 + \pi_1\hat{\rho}_0 - \pi_1\hat{\rho}_0)\} \tag{11}$$

$$= \pi_0 - \pi_1 \mathrm{Tr}\{\hat{\Lambda}_0(\hat{\rho}_0 - \hat{\rho}_1)\} + (\pi_1 - \pi_0)\mathrm{Tr}\{\hat{\Lambda}_0\hat{\rho}_0\} \tag{12}$$

$$\geq \pi_0 - \pi_1 \mathrm{Tr}\{\hat{\Lambda}_0(\hat{\rho}_0 - \hat{\rho}_1)\} \tag{13}$$

$$\geq \pi_0 - \frac{\pi_1}{2}\|\hat{\rho}_0 - \hat{\rho}_1\|_1, \tag{14}$$

where (13) follows since $\pi_0 \leq \pi_1$ and $\mathrm{Tr}\{\hat{\Lambda}_0\hat{\rho}_0\} \geq 0$, and (14) follows by Lemma 1.

When $\pi_1 \leq \pi_0$, the same steps are used with substitution of $\hat{\Lambda}_0 = \hat{I} - \hat{\Lambda}_1$ in (7) instead of $\hat{\Lambda}_1 = \hat{I} - \hat{\Lambda}_0$, and replacement of $\pi_1\hat{\rho}_0 - \pi_1\hat{\rho}_0$ with $\pi_0\hat{\rho}_1 - \pi_0\hat{\rho}_1$ inside the trace in (11). This yields

$$\mathbb{P}_{\mathrm{e}}^{(\mathrm{w})} \geq \pi_1 - \frac{\pi_0}{2}\|\hat{\rho}_0 - \hat{\rho}_1\|_1, \tag{15}$$

and the lemma. □

Thus, while Lemma 2 demonstrates that additional information about the likelihood of Alice transmitting (in the form of unequal prior probabilities $\pi_0 \neq \pi_1$) helps Willie, the square-root law still holds via the bounds on the trace distance $\|\hat{\rho}_0 - \hat{\rho}_1\|_1$.

Supplementary Note 3. Covert Communication Theorems

Here we re-state the theorems from the main paper and provide their proofs.

Theorem 1 (Insufficiency of pure-loss for covert communication)**.** *Suppose Willie has a pure-loss channel from Alice and is limited only by the laws of physics in his receiver measurement choice. Then Alice cannot communicate to Bob reliably and covertly even if Alice and Bob have access to a pre-shared secret of unbounded size, an unattenuated observation of the transmission, and a quantum-optimal receiver.*

In the proof of this theorem we denote a tensor product of n Fock (or photon number) states by $|\mathbf{u}\rangle \equiv |u_1\rangle \otimes |u_2\rangle \otimes \cdots \otimes |u_n\rangle$, where vector $\mathbf{u} \in \mathbb{N}_0^n$ and $\mathbb{N}_0$ is the set of non-negative integers. Specifically, $|\mathbf{0}\rangle \equiv |0\rangle^{\otimes n}$. Before proceeding with the proof, we prove the following lemma:

Lemma 3. *Given the input of n-mode vacuum state $|\mathbf{0}\rangle^{E^n}$ on the "environment" port and an n-mode entangled state $|\psi\rangle^{A^n} = \sum_{\mathbf{k}} a_{\mathbf{k}} |\mathbf{k}\rangle^{A^n}$ on the "Alice" port of a beamsplitter with transmissivity $\eta_{\mathrm{b}} = 1 - \eta_{\mathrm{w}}$, the diagonal elements of the output state ρ^{W^n} on the "Willie" port can be expressed in the n-fold Fock state basis as follows:*

$${}^{W^n}\langle\mathbf{s}|\,\hat{\rho}^{W^n}\,|\mathbf{s}\rangle^{W^n} = \sum_{\mathbf{k}\in\mathbb{N}_0^n} |a_{\mathbf{k}}|^2 \prod_{i=1}^{n} \binom{k_i}{s_i} (1-\eta_{\mathrm{w}})^{k_i - s_i}\eta_{\mathrm{w}}^{s_i}. \tag{16}$$

Proof. A beamsplitter can be described as a unitary transformation U_{BS} from the two input modes (Alice's and the environment's ports) to the two output modes (Bob's and Willie's ports). Given a Fock state input $|t\rangle^A$ on Alice's port and vacuum input $|0\rangle^E$ on the environment's port, the output at Bob's and Willie's ports is described as follows [12, Section IV.D]:

$$U_{\mathrm{BS}}\,|t\rangle^A |0\rangle^E = \sum_{m=0}^{t} \sqrt{\binom{t}{m}\eta_{\mathrm{w}}^m (1-\eta_{\mathrm{w}})^{t-m}}\,|m\rangle^W |t-m\rangle^B .$$

Thus,

$$U_{\mathrm{BS}}^{\otimes n}\left|\mathbf{t}\right\rangle^{A^n}\left|\mathbf{0}\right\rangle^{E^n}=\bigotimes_{i=1}^{n}\sum_{m_i=0}^{t_i}\sqrt{\binom{t_i}{m_i}\eta_{\mathrm{w}}^{m_i}(1-\eta_{\mathrm{w}})^{t_i-m_i}}\left|m_i\right\rangle^{W_i}\left|t_i-m_i\right\rangle^{B_i},$$

which implies

$$U_{\mathrm{BS}}^{\otimes n}\left|\psi\right\rangle^{A^n}\left|\mathbf{0}\right\rangle^{E^n}=\sum_{\mathbf{t}\in\mathbb{N}_0^n}a_{\mathbf{t}}\bigotimes_{i=1}^{n}\sum_{m_i=0}^{t_i}\sqrt{\binom{t_i}{m_i}\eta_{\mathrm{w}}^{m_i}(1-\eta_{\mathrm{w}})^{t_i-m_i}}\left|m_i\right\rangle^{W_i}\left|t_i-m_i\right\rangle^{B_i}\equiv\left|\phi\right\rangle^{W^nB^n}.$$

Now, the partial trace of the output state $\rho^{BW}=\left|\phi\right\rangle^{W^nB^n}$ over Bob's system reveals Willie's output state:

$$\begin{aligned}\rho^{W^n}&=\mathrm{Tr}_{B^n}\left[\left|\phi\right\rangle^{W^nB^n}\,{}^{W^nB^n}\!\left\langle\phi\right|\right]\\&=\sum_{\mathbf{x}\in\mathbb{N}_0^n}\left|{}^{B^n}\!\left\langle\mathbf{x}|\phi\right\rangle^{W^nB^n}\right|^2,\end{aligned}$$

with

$$\begin{aligned}{}^{B^n}\!\left\langle\mathbf{x}|\phi\right\rangle^{W^nB^n}&=\sum_{\mathbf{t}\in\mathbb{N}_0^n}a_{\mathbf{t}}\bigotimes_{i=1}^{n}\sum_{m_i=0}^{t_i}\sqrt{\binom{t_i}{m_i}\eta_{\mathrm{w}}^{m_i}(1-\eta_{\mathrm{w}})^{t_i-m_i}}\left|m_i\right\rangle^{W_i}\,{}^{B_i}\!\left\langle x_i|t_i-m_i\right\rangle^{B_i}\\&=\sum_{\mathbf{t}\in\mathbb{N}_0^n}a_{\mathbf{t}}\bigotimes_{i=1}^{n}\sqrt{\binom{t_i}{x_i}\eta_{\mathrm{w}}^{t_i-x_i}(1-\eta_{\mathrm{w}})^{x_i}}\left|t_i-x_i\right\rangle^{W_i},\end{aligned}\tag{17}$$

where equation (17) is because the Fock states are orthogonal. Thus,

$${}^{W^n}\!\left\langle\mathbf{s}\right|\hat{\rho}^{W^n}\left|\mathbf{s}\right\rangle^{W^n}=\sum_{\mathbf{x}\in\mathbb{N}_0^n}\left|{}^{W^n}\!\left\langle\mathbf{s}\right|\,{}^{B^n}\!\left\langle\mathbf{x}|\phi\right\rangle^{W^nB^n}\right|^2,\tag{18}$$

where

$$\begin{aligned}{}^{W^n}\!\left\langle\mathbf{s}\right|\,{}^{B^n}\!\left\langle\mathbf{x}|\phi\right\rangle^{W^nB^n}&=\sum_{\mathbf{t}\in\mathbb{N}_0^n}a_{\mathbf{t}}\prod_{i=1}^{n}\sqrt{\binom{t_i}{x_i}\eta_{\mathrm{w}}^{t_i-x_i}(1-\eta_{\mathrm{w}})^{x_i}}\delta_{s_i,t_i-x_i}\\&=a_{\mathbf{x}+\mathbf{s}}\prod_{i=1}^{n}\sqrt{\binom{x_i+s_i}{x_i}\eta_{\mathrm{w}}^{s_i}(1-\eta_{\mathrm{w}})^{x_i}},\end{aligned}\tag{19}$$

with $\delta_{a,b}=\begin{cases}1 & \text{if } a=b\\ 0 & \text{otherwise}\end{cases}$. Substituting $\mathbf{k}=\mathbf{x}+\mathbf{s}$ into equation (19) and substituting the right-hand side (RHS) of (19) into equation (18) yields

$$\begin{aligned}{}^{W^n}\!\left\langle\mathbf{s}\right|\hat{\rho}^{W^n}\left|\mathbf{s}\right\rangle^{W^n}&=\sum_{\mathbf{k}\in\mathbb{N}_0^n}\left|a_{\mathbf{k}}\prod_{i=1}^{n}\sqrt{\binom{k_i}{s_i}\eta_{\mathrm{w}}^{s_i}(1-\eta_{\mathrm{w}})^{k_i-s_i}}\right|^2\\&=\sum_{\mathbf{k}\in\mathbb{N}_0^n}\left|a_{\mathbf{k}}\right|^2\prod_{i=1}^{n}\binom{k_i}{s_i}\eta_{\mathrm{w}}^{s_i}(1-\eta_{\mathrm{w}})^{k_i-s_i}\end{aligned}\tag{20}$$

where equation (20) is because $\eta_{\mathrm{w}}\in[0,1)$. □

Proof. (Theorem 1) Alice sends one of 2^M (equally likely) M-bit messages by choosing an element from an arbitrary codebook $\{\hat{\rho}_x^{A^n}, x=1,\ldots,2^M\}$, where a state $\hat{\rho}_x^{A^n} = |\psi_x\rangle^{A^n A^n}\langle\psi_x|$ encodes an M-bit message W_x. $|\psi_x\rangle^{A^n} = \sum_{\mathbf{k}\in\mathbb{N}_0^n} a_{\mathbf{k}}(x)\,|\mathbf{k}\rangle^{A^n}$ is a general n-mode pure state, where $|\mathbf{k}\rangle \equiv |k_1\rangle \otimes |k_2\rangle \otimes \cdots \otimes |k_n\rangle$ is a tensor product of n Fock states. We limit our analysis to pure input states since, by convexity, using mixed states as inputs can only degrade the performance (since that is equivalent to transmitting a randomly chosen pure state from an ensemble and discarding the knowledge of that choice).

Let Willie use an ideal SPD on all n modes, given by positive operator-valued measure (POVM) $\left\{|0\rangle\langle 0|, \sum_{j=1}^{\infty}|j\rangle\langle j|\right\}^{\otimes n}$. When W_u is transmitted, Willie's hypothesis test reduces to discriminating between the states

$$\hat{\rho}_0^{W^n} = |\mathbf{0}\rangle^{W^n W^n}\langle\mathbf{0}| \text{ and} \tag{21}$$

$$\hat{\rho}_1^{W^n} = \hat{\rho}_u^{W^n}, \tag{22}$$

where $\hat{\rho}_u^{W^n}$ is the output state of a pure-loss channel with transmissivity η_{w} corresponding to an input state $\hat{\rho}_u^{A^n}$. Thus, Willie's average error probability is:

$$\mathbb{P}_{\mathrm{e}}^{(\mathrm{w})} = \frac{1}{2^{M+1}}\sum_{u=1}^{2^M} {}^{W^n}\langle\mathbf{0}|\,\hat{\rho}_u^{W^n}\,|\mathbf{0}\rangle^{W^n}, \tag{23}$$

since messages are sent equiprobably. Note that the error is entirely because of missed codeword detections, as Willie's receiver never raises a false alarm. By Lemma 3,

$$\begin{aligned} {}^{W^n}\langle\mathbf{0}|\,\hat{\rho}_u^{W}\,|\mathbf{0}\rangle^{W^n} &= \sum_{\mathbf{k}\in\mathbb{N}_0^n} |a_{\mathbf{k}}(u)|^2 (1-\eta_{\mathrm{w}})^{\sum_{i=1}^n k_i} \\ &\le |a_{\mathbf{0}}(u)|^2 + (1-|a_{\mathbf{0}}(u)|^2)(1-\eta_{\mathrm{w}}) \\ &= 1-\eta_{\mathrm{w}}\left(1-|a_{\mathbf{0}}(u)|^2\right). \end{aligned} \tag{24}$$

Substituting equation (24) into equation (23) yields:

$$\mathbb{P}_{\mathrm{e}}^{(\mathrm{w})} \le \frac{1}{2} - \frac{\eta_{\mathrm{w}}}{2}\left(1-\frac{1}{2^M}\sum_{u=1}^{2^M}|a_{\mathbf{0}}(u)|^2\right).$$

Thus, to ensure $\mathbb{P}_{\mathrm{e}}^{(\mathrm{w})} \ge \frac{1}{2}-\epsilon$, Alice must use a codebook with the probability of transmitting zero photons:

$$\frac{1}{2^M}\sum_{u=1}^{2^M}|a_{\mathbf{0}}(u)|^2 \ge 1-\frac{2\epsilon}{\eta_{\mathrm{w}}}. \tag{25}$$

Equation (25) can be restated as an upper bound on the probability of transmitting one or more photons:

$$\frac{1}{2^M}\sum_{u=1}^{2^M}\left(1-|a_{\mathbf{0}}(u)|^2\right) \le \frac{2\epsilon}{\eta_{\mathrm{w}}}. \tag{26}$$

Now we show that there exists an interval $(0,\epsilon_0]$, $\epsilon_0>0$ such that if $\epsilon\in(0,\epsilon_0]$, Bob's average decoding error probability $\mathbb{P}_{\mathrm{e}}^{(\mathrm{b})} \ge \delta_0$ where $\delta_0>0$, thus making covert communication over a pure-loss channel unreliable.

Denote by $E_{u\to v}$ the event that the transmitted message W_u is decoded by Bob as $W_v \ne W_u$. Given that W_u is transmitted, the decoding error probability is the probability of the union of events $\cup_{v=0,v\ne u}^{2^M} E_{u\to v}$. Let Bob choose a POVM $\{\Lambda_j^*\}$ that minimizes the average probability of error over n modes:

$$\mathbb{P}_{\mathrm{e}}^{(\mathrm{b})} = \inf_{\{\Lambda_j\}}\frac{1}{2^M}\sum_{u=1}^{2^M}\mathbb{P}\left(\cup_{v=0,v\ne u}^{2^M}E_{u\to v}\right). \tag{27}$$

Now consider a codebook that meets the necessary condition for covert communication given in equation (26). Define the subset of this codebook $\left\{\hat{\rho}_u^{A^n}, u \in \mathcal{A}\right\}$ where $\mathcal{A} = \left\{u : 1 - |a_{\mathbf{0}}(u)|^2 \leq \frac{4\epsilon}{\eta_{\mathrm{w}}}\right\}$. We lower-bound (27) as follows:

$$\mathbb{P}_{\mathrm{e}}^{(\mathrm{b})} = \frac{1}{2^M} \sum_{u \in \bar{\mathcal{A}}} \mathbb{P}\left(\cup_{v=0, v\neq u}^{2^M} E_{u\to v}\right) + \frac{1}{2^M} \sum_{u \in \mathcal{A}} \mathbb{P}\left(\cup_{v=0, v\neq u}^{2^M} E_{u\to v}\right) \tag{28}$$

$$\geq \frac{1}{2^M} \sum_{u \in \mathcal{A}} \mathbb{P}\left(\cup_{v=0, v\neq u}^{2^M} E_{u\to v}\right), \tag{29}$$

where the probabilities in equation (28) are with respect to the POVM $\{\Lambda_j^*\}$ that minimizes equation (27) over the entire codebook. Without loss of generality, let's assume that $|\mathcal{A}|$ is even, and split $\mathcal{A}$ into two equal-sized non-overlapping subsets $\mathcal{A}^{(\mathrm{left})}$ and $\mathcal{A}^{(\mathrm{right})}$ (formally, $\mathcal{A}^{(\mathrm{left})} \cup \mathcal{A}^{(\mathrm{right})} = \mathcal{A}$, $\mathcal{A}^{(\mathrm{left})} \cap \mathcal{A}^{(\mathrm{right})} = \emptyset$, and $|\mathcal{A}^{(\mathrm{left})}| = |\mathcal{A}^{(\mathrm{right})}|$). Let $g : \mathcal{A}^{(\mathrm{left})} \to \mathcal{A}^{(\mathrm{right})}$ be a bijection. We can thus re-write (29):

$$\mathbb{P}_{\mathrm{e}}^{(\mathrm{b})} \geq \frac{1}{2^M} \sum_{u \in \mathcal{A}^{(\mathrm{left})}} 2\left(\frac{\mathbb{P}\left(\cup_{v=0, v\neq u}^{2^M} E_{u\to v}\right)}{2} + \frac{\mathbb{P}\left(\cup_{v=0, v\neq g(u)}^{2^M} E_{g(u)\to v}\right)}{2}\right)$$

$$\geq \frac{1}{2^M} \sum_{u \in \mathcal{A}^{(\mathrm{left})}} 2\left(\frac{\mathbb{P}\left(E_{u\to g(u)}\right)}{2} + \frac{\mathbb{P}\left(E_{g(u)\to u}\right)}{2}\right), \tag{30}$$

where the second lower bound is because the events $E_{u\to g(u)}$ and $E_{g(u)\to u}$ are contained in the unions $\cup_{v=0, v\neq u}^{2^M} E_{u\to v}$ and $\cup_{v=0, v\neq g(u)}^{2^M} E_{g(u)\to v}$, respectively. The summation term in equation (30),

$$\mathbb{P}_{\mathrm{e}}(u) \equiv \frac{\mathbb{P}\left(E_{u\to g(u)}\right)}{2} + \frac{\mathbb{P}\left(E_{g(u)\to u}\right)}{2}, \tag{31}$$

is Bob's average probability of error when Alice only sends messages W_u and $W_{g(u)}$ equiprobably. We thus reduce the analytically intractable problem of discriminating between many states in equation (27) to a quantum binary hypothesis test.

The lower bound on the probability of error in discriminating two received codewords is obtained by lower-bounding the probability of error in discriminating two codewords before they are sent (this is equivalent to Bob having an unattenuated unity-transmissivity channel from Alice). Recalling that $\hat{\rho}_u^{A^n} = |\psi_u\rangle^{A^n A^n}\langle\psi_u|$ and $\hat{\rho}_{g(u)}^{A^n} = \left|\psi_{g(u)}\right\rangle^{A^n A^n}\left\langle\psi_{g(u)}\right|$ are pure states, the lower bound on the probability of error in discriminating between $\left|\psi_u^{A^n}\right\rangle$ and $\left|\psi_{g(u)}^{A^n}\right\rangle$ is [13, Chapter IV.2 (c), Equation (2.34)]:

$$\mathbb{P}_{\mathrm{e}}(u) \geq \left[1 - \sqrt{1 - F\left(|\psi_u\rangle^{A^n}, \left|\psi_{g(u)}\right\rangle^{A^n}\right)}\right] \Big/ 2\,, \tag{32}$$

where $F(|\psi\rangle, |\phi\rangle) = |\langle\psi|\phi\rangle|^2$ is the fidelity between the pure states $|\psi\rangle$ and $|\phi\rangle$. Lower-bounding $F\left(|\psi_u\rangle^{A^n}, \left|\psi_{g(u)}\right\rangle^{A^n}\right)$ lower-bounds the RHS of equation (32). For pure states $|\psi\rangle$ and $|\phi\rangle$, $F(|\psi\rangle, |\phi\rangle) = 1 - \left(\frac{1}{2}\| \,|\psi\rangle\langle\psi| - |\phi\rangle\langle\phi|\, \|_1\right)^2$, where $\|\rho - \sigma\|_1$ is the trace distance [11, Equation (9.134)]. Thus,

$$F\left(|\psi_u\rangle^{A^n}, \left|\psi_{g(u)}\right\rangle^{A^n}\right) = 1 - \left(\frac{1}{2}\|\hat{\rho}_u^{A^n} - \hat{\rho}_{g(u)}^{A^n}\|_1\right)^2$$

$$\geq 1 - \left(\frac{\|\hat{\rho}_u^{A^n} - |\mathbf{0}\rangle^{A^n A^n}\langle\mathbf{0}|\,\|_1}{2} + \frac{\|\hat{\rho}_{g(u)}^{A^n} - |\mathbf{0}\rangle^{A^n A^n}\langle\mathbf{0}|\,\|_1}{2}\right)^2$$

$$= 1 - \left(\sqrt{1 - \left|{}^{A^n}\langle\mathbf{0}|\psi_u\rangle^{A^n}\right|^2} + \sqrt{1 - \left|{}^{A^n}\langle\mathbf{0}|\psi_{g(u)}\rangle^{A^n}\right|^2}\right)^2, \tag{33}$$

where the inequality is from the triangle inequality for trace distance. Substituting (33) into (32) yields:

$$\mathbb{P}_{\mathrm{e}}(u) \geq \left[1-\sqrt{1-\left|{}^{A^n}\langle\mathbf{0}|\psi_u\rangle^{A^n}\right|^2}-\sqrt{1-\left|{}^{A^n}\langle\mathbf{0}|\psi_{g(u)}\rangle^{A^n}\right|^2}\right]\Big/2\,. \tag{34}$$

Since $\left|{}^{A^n}\langle\mathbf{0}|\psi_u\rangle^{A^n}\right|^2=|a_{\mathbf{0}}(u)|^2$ and, by the construction of $\mathcal{A}$, $1-|a_{\mathbf{0}}(u)|^2\leq\frac{4\epsilon}{\eta_{\mathrm{w}}}$ and $1-|a_{\mathbf{0}}(g(u))|^2\leq\frac{4\epsilon}{\eta_{\mathrm{w}}}$, we have:

$$\mathbb{P}_{\mathrm{e}}(u)\geq\frac{1}{2}-2\sqrt{\frac{\epsilon}{\eta_{\mathrm{w}}}}. \tag{35}$$

Recalling the definition of $\mathbb{P}_{\mathrm{e}}(u)$ in equation (31), we substitute (35) into (30) to obtain:

$$\mathbb{P}_{\mathrm{e}}^{(\mathrm{b})}\geq\frac{|\mathcal{A}|}{2^M}\left(\frac{1}{2}-2\sqrt{\frac{\epsilon}{\eta_{\mathrm{w}}}}\right), \tag{36}$$

Now, re-stating the condition for covert communication (26) yields:

$$\begin{aligned}\frac{2\epsilon}{\eta_{\mathrm{w}}}&\geq\frac{1}{2^M}\sum_{u\in\overline{\mathcal{A}}}\left(1-|a_{\mathbf{0}}(u)|^2\right)\\&\geq\frac{\left(2^M-|\mathcal{A}|\right)}{2^M}\frac{4\epsilon}{\eta_{\mathrm{w}}}\end{aligned} \tag{37}$$

with equality (37) because $1-|a_{\mathbf{0}}(u)|^2>\frac{4\epsilon}{\eta_{\mathrm{w}}}$ for all codewords in $\overline{\mathcal{A}}$ by the construction of $\mathcal{A}$. Solving inequality in (37) for $\frac{|\mathcal{A}|}{2^M}$ yields the lower bound on the fraction of the codewords in $\mathcal{A}$,

$$\frac{|\mathcal{A}|}{2^M}\geq\frac{1}{2}. \tag{38}$$

Combining equations (36) and (38) results in a positive lower bound on Bob's probability of decoding error $\mathbb{P}_{\mathrm{e}}^{(\mathrm{b})}\geq\frac{1}{4}-\sqrt{\frac{\epsilon}{\eta_{\mathrm{w}}}}$ for $\epsilon\in\left(0,\frac{\eta_{\mathrm{w}}}{16}\right]$ and any n, and demonstrates that reliable covert communication over a pure-loss channel is impossible. □

Remark—The minimum probability of discrimination error between the states given by equations (21) and (22) satisfies [14, Section III]:

$$\frac{1-\sqrt{1-{}^{W^n}\langle\mathbf{0}|\,\hat{\rho}_u^{W^n}\,|\mathbf{0}\rangle^{W^n}}}{2}\leq\min\mathbb{P}_{\mathrm{e}}^{(\mathrm{w})}\leq\frac{1}{2}{}^{W^n}\langle\mathbf{0}|\,\hat{\rho}_u^{W^n}\,|\mathbf{0}\rangle^{W^n}\,.$$

Since $\frac{{}^{W^n}\langle\mathbf{0}|\hat{\rho}_u^{W^n}|\mathbf{0}\rangle^{W^n}}{4}\leq\frac{1-\sqrt{1-{}^{W^n}\langle\mathbf{0}|\hat{\rho}_u^{W^n}|\mathbf{0}\rangle^{W^n}}}{2}$, the error probability for the SPD is at most twice that of an optimal discriminator. Thus, the SPD is an asymptotically optimal detector when the channel from Alice is pure-loss. Since the photon number resolving (PNR) receiver, given by the POVM elements $\{|0\rangle\langle 0|,|1\rangle\langle 1|,|2\rangle\langle 2|,\ldots\}^{\otimes n}$, could be used to mimic the SPD with the detection event threshold set at one photon, the PNR receiver is also asymptotically optimal in this scenario.

Theorem 2 (Square-root law for the thermal noise channel)**.** *Suppose Willie has access to an arbitrarily complex receiver measurement as permitted by the laws of quantum physics and can capture all the photons transmitted by Alice that do not reach Bob. Let Willie's channel from Alice be subject to noise from a thermal environment that injects $\bar{n}_{\mathrm{T}}>0$ photons per mode on average, and let Alice and Bob share a secret of sufficient length before communicating. Then Alice can lower-bound Willie's detection error probability $\mathbb{P}_{\mathrm{e}}^{(\mathrm{w})}\geq\frac{1}{2}-\epsilon$ for any $\epsilon>0$ while reliably transmitting $\mathcal{O}(\sqrt{n})$ bits to Bob in n modes even if Bob only has access to a (sub-optimal) coherent detection receiver, such as an optical homodyne detector.*

First, we define quantum relative entropy and prove a lemma:

Definition 2. *Quantum relative entropy between states* $\hat{\rho}_0$ *and* $\hat{\rho}_1$ *is* $D(\hat{\rho}_0\|\hat{\rho}_1) \equiv \operatorname{Tr}\{\hat{\rho}_0(\ln\hat{\rho}_0 - \ln\hat{\rho}_1)\}$.

Lemma 4 (Quantum relative entropy between two thermal states)**.** *If* $\hat{\rho}_0 = \sum_{n=0}^{\infty}\frac{\bar{n}_0^n}{(1+\bar{n}_0)^{1+n}}|n\rangle\langle n|$ *and* $\hat{\rho}_1 = \sum_{n=0}^{\infty}\frac{\bar{n}_1^n}{(1+\bar{n}_1)^{1+n}}|n\rangle\langle n|$, *then* $D(\hat{\rho}_0\|\hat{\rho}_1) = \bar{n}_0\ln\frac{\bar{n}_0(1+\bar{n}_1)}{\bar{n}_1(1+\bar{n}_0)} + \ln\frac{1+\bar{n}_1}{1+\bar{n}_0}$

Proof. Express $D(\hat{\rho}_0\|\hat{\rho}_1) = -\operatorname{Tr}\{\hat{\rho}_0(\ln\hat{\rho}_1)\} - S(\hat{\rho}_0)$, where $S(\hat{\rho}_0) \equiv -\operatorname{Tr}[\hat{\rho}_0\ln\hat{\rho}_0]$ is the von Neumann entropy of the state $\hat{\rho}_0$:

$$S(\hat{\rho}_0) = \ln(1+\bar{n}_0) + \bar{n}_0\ln\left(1+\frac{1}{\bar{n}_0}\right). \tag{39}$$

Now,

$$\begin{aligned}
\operatorname{Tr}[\hat{\rho}_0\ln\hat{\rho}_1] &= \operatorname{Tr}\left[\left(\sum_{n=0}^{\infty}\frac{\bar{n}_0^n}{(1+\bar{n}_0)^{1+n}}|n\rangle\langle n|\right)\left(\sum_{n=0}^{\infty}\ln\frac{\bar{n}_1^n}{(1+\bar{n}_1)^{1+n}}|n\rangle\langle n|\right)\right]\\
&= \sum_{n=0}^{\infty}\frac{\bar{n}_0^n}{(1+\bar{n}_0)^{1+n}}\ln\frac{\bar{n}_1^n}{(1+\bar{n}_1)^{1+n}}\\
&= \frac{1}{1+\bar{n}_0}\ln\frac{1}{1+\bar{n}_1}\sum_{n=0}^{\infty}\left(\frac{\bar{n}_0}{1+\bar{n}_0}\right)^n + \ln\frac{\bar{n}_1}{1+\bar{n}_1}\sum_{n=0}^{\infty}\frac{n}{1+\bar{n}_0}\left(\frac{\bar{n}_0}{1+\bar{n}_0}\right)^n\\
&= \ln\frac{1}{1+\bar{n}_1} + \bar{n}_0\ln\frac{\bar{n}_1}{1+\bar{n}_1}
\end{aligned} \tag{40}$$

where (40) is because the geometric series $\sum_{n=0}^{\infty}\left(\frac{\bar{n}_0}{1+\bar{n}_0}\right)^n = \left(1-\frac{\bar{n}_0}{1+\bar{n}_0}\right)^{-1}$ and $\sum_{n=0}^{\infty}\frac{n}{1+\bar{n}_0}\left(\frac{\bar{n}_0}{1+\bar{n}_0}\right)^n = \bar{n}_0$ is the expression for the mean of geometrically-distributed random variable $X \sim \operatorname{Geom}\left(\frac{1}{1+\bar{n}_0}\right)$. Combining (39) and (40) yields the lemma. □

Proof. (Theorem 2) Construction: Let Alice use a zero-mean isotropic Gaussian-distributed coherent state input $\{p(\alpha), |\alpha\rangle\}$, where $\alpha \in \mathbb{C}$, $p(\alpha) = e^{-|\alpha|^2/\bar{n}}/\pi\bar{n}$ with mean photon number per symbol $\bar{n} = \int_{\mathbb{C}}|\alpha|^2 p(\alpha)\mathrm{d}^2\alpha$. Alice encodes M-bit blocks of input into codewords of length n symbols by generating 2^M codewords $\{\bigotimes_{i=1}^n|\alpha_i\rangle_k\}_{k=1}^{2^M}$, each according to $p(\bigotimes_{i=1}^n|\alpha_i\rangle) = \prod_{i=1}^n p(\alpha_i)$, where $\bigotimes_{i=1}^n|\alpha_i\rangle = |\alpha_1\ldots\alpha_n\rangle$ is an n-mode tensor-product coherent state. The codebook is used only once to send a single message and is kept secret from Willie, though he knows how it is constructed.

Analysis (Willie): Since Willie does not have access to Alice's codebook, Willie has to discriminate between the following n-copy quantum states:

$$\begin{aligned}
\hat{\rho}_0^{\otimes n} &= \left(\sum_{i=0}^{\infty}\frac{(\eta_{\mathrm{b}}\bar{n}_{\mathrm{T}})^i}{(1+\eta_{\mathrm{b}}\bar{n}_{\mathrm{T}})^{1+i}}|i\rangle\langle i|\right)^{\otimes n}, \text{ and}\\
\hat{\rho}_1^{\otimes n} &= \left(\sum_{i=0}^{\infty}\frac{((1-\eta_{\mathrm{b}})\bar{n}+\eta_{\mathrm{b}}\bar{n}_{\mathrm{T}})^i}{(1+(1-\eta_{\mathrm{b}})\bar{n}+\eta_{\mathrm{b}}\bar{n}_{\mathrm{T}})^{1+i}}|i\rangle\langle i|\right)^{\otimes n}.
\end{aligned}$$

By Lemma 2, Willie's average probability of error in discriminating between $\hat{\rho}_0^{\otimes n}$ and $\hat{\rho}_1^{\otimes n}$ is:

$$\mathbb{P}_e^{(\mathrm{w})} \geq \frac{1}{2}\left[1-\frac{1}{2}\|\hat{\rho}_1^{\otimes n}-\hat{\rho}_0^{\otimes n}\|_1\right],$$

where the minimum in this case is attained by a PNR detection. The trace distance $\|\hat{\rho}_0 - \hat{\rho}_1\|_1$ between states $\hat{\rho}_1$ and $\hat{\rho}_1$ is upper-bounded the quantum relative entropy (QRE) using quantum Pinsker's Inequality [11, Theorem 11.9.5] as follows:

$$\|\hat{\rho}_0 - \hat{\rho}_1\|_1 \leq \sqrt{2D(\hat{\rho}_0\|\hat{\rho}_1)},$$

Thus,

$$\mathbb{P}_e^{(\mathrm{w})} \geq \frac{1}{2} - \sqrt{\frac{1}{8}D(\hat{\rho}_0^{\otimes n}\|\hat{\rho}_1^{\otimes n})} \tag{41}$$

and ensuring that

$$D(\hat{\rho}_0^{\otimes n}\|\hat{\rho}_1^{\otimes n}) \leq 8\epsilon^2 \tag{42}$$

ensures that $\mathbb{P}_e^{(\mathrm{w})} \geq \frac{1}{2} - \epsilon$ over n modes. QRE is additive for tensor product states:

$$D(\hat{\rho}_0^{\otimes n}\|\hat{\rho}_1^{\otimes n}) = nD(\hat{\rho}_0\|\hat{\rho}_1). \tag{43}$$

By Lemma 4,

$$D(\hat{\rho}_0\|\hat{\rho}_1) = \eta_\mathrm{b}\bar{n}_\mathrm{T} \ln \frac{(1+(1-\eta_\mathrm{b})\bar{n}+\eta_\mathrm{b}\bar{n}_\mathrm{T})\eta_\mathrm{b}\bar{n}_\mathrm{T}}{((1-\eta_\mathrm{b})\bar{n}+\eta_\mathrm{b}\bar{n}_\mathrm{T})(1+\eta_\mathrm{b}\bar{n}_\mathrm{T})} + \ln \frac{1+(1-\eta_\mathrm{b})\bar{n}+\eta_\mathrm{b}\bar{n}_\mathrm{T}}{1+\eta_\mathrm{b}\bar{n}_\mathrm{T}}. \tag{44}$$

The first two terms of the Taylor series expansion of the RHS of (44) with respect to $\bar{n}$ at $\bar{n} = 0$ are zero and the fourth term is negative. Thus, using Taylor's Theorem with the remainder, we can upper-bound equation (44) by the third term as follows:

$$D(\hat{\rho}_0\|\hat{\rho}_1) \leq \frac{(1-\eta_\mathrm{b})^2\bar{n}^2}{2\eta_\mathrm{b}\bar{n}_\mathrm{T}(1+\eta_\mathrm{b}\bar{n}_\mathrm{T})}. \tag{45}$$

Combining equations (41), (43), and (45) yields:

$$\mathbb{P}_\mathrm{e}^{(\mathrm{w})} \geq \frac{1}{2} - \frac{(1-\eta_\mathrm{b})\bar{n}\sqrt{n}}{4\sqrt{\eta_\mathrm{b}\bar{n}_\mathrm{T}(1+\eta_\mathrm{b}\bar{n}_\mathrm{T})}} \tag{46}$$

Therefore, setting

$$\bar{n} = \frac{4\epsilon\sqrt{\eta_\mathrm{b}\bar{n}_\mathrm{T}(1+\eta_\mathrm{b}\bar{n}_\mathrm{T})}}{\sqrt{n}(1-\eta_\mathrm{b})} \tag{47}$$

ensures that Willie's error probability is lower-bounded by $\mathbb{P}_e^{(\mathrm{w})} \geq \frac{1}{2} - \epsilon$ over n modes.

Analysis (Bob): Suppose Bob uses a coherent detection receiver. A homodyne receiver, which is more efficient than a heterodyne receiver in the low photon number regime [15], induces an AWGN channel with noise power

$$\sigma_b^2 = \frac{2(1-\eta_\mathrm{b})\bar{n}_\mathrm{T}+1}{4\eta_\mathrm{b}}. \tag{48}$$

Since Alice uses Gaussian modulation with symbol power $\bar{n}$ defined in equation (47), we can upper-bound $\mathbb{P}_\mathrm{e}^{(\mathrm{b})}$ by [16, Equation (9)]:

$$\mathbb{P}_e^{(\mathrm{b})} \leq 2^{M-\frac{n}{2}\log_2\left(1+\bar{n}/2\sigma_b^2\right)}. \tag{49}$$

Substitution of (47) and (48) into (49) shows that $\mathcal{O}(\sqrt{n})$ bits can be covertly transmitted from Alice to Bob with $\mathbb{P}_e^{(\mathrm{b})} < \delta$ for arbitrary $\delta > 0$ given large enough n. □

Some remarks are in order:

Unknown thermal noise—To achieve the square-root law above, Alice and Bob need at least a lower bound to the number of photons $\bar{n}_{\mathrm{T}}$ injected by the thermal environment. In practice, Planck's law yields such for the given transmitter center frequency and receiver temperature. However, even when the lower bound on $\bar{n}_{\mathrm{T}}$ is unavailable, Alice and Bob can still transmit $o(\sqrt{n})$ bits over n modes (where we denote by $f(n) = o(g(n))$ an upper bound on $f(n)$ that is not asymptotically tight [17]). To achieve this, we borrow the technique from [16], where, rather than using (47), Alice and Bob set the mean photon number per symbol $\bar{n} = \frac{4\epsilon f(n)\sqrt{\eta_{\mathrm{b}}\bar{n}_{\mathrm{T}}(1+\eta_{\mathrm{b}}\bar{n}_{\mathrm{T}})}}{\sqrt{n}(1-\eta_{\mathrm{b}})}$, with $f(n)$ selected such that $f(n) = o(1)$ and $f(n) = \omega(1/\sqrt{n})$. Then, $\lim_{n\to\infty} \mathbb{P}_{\mathrm{e}}^{(\mathrm{w})} = \frac{1}{2}$ as long as $\bar{n}_{\mathrm{T}} > 0$ and $\eta_{\mathrm{b}} > 0$.

Active adversary—Willie can actively inject additional light into the channel, increasing Bob's noise power σ_b^2 and, thus, decreasing his signal-to-noise ratio (SNR). However, the square-root law holds as long as $\sigma_b^2 < \infty$. Thus, while Willie can certainly reduce the number of transmitted covert bits by actively jamming the channel, he cannot eliminate covert communication unless he injects noise at infinite power all the time.

Performance analysis—We can obtain a lower bound on the maximum number of covert bits that Alice can reliably transmit to Bob by substituting (47) and (48) into (49), performing the Taylor series expansion of $\log(1+x)$ at $x = 0$, and solving for M:

$$M \geq \frac{\sqrt{n}\epsilon C_{\mathrm{T}}}{2\ln 2} + \log_2(\delta) - \frac{\epsilon^2 C_{\mathrm{T}}^2}{4\ln 2}, \tag{50}$$

where δ is the desired decoding error probability, and

$$C_{\mathrm{T}} = \frac{8\sqrt{\eta_{\mathrm{b}}\bar{n}_{\mathrm{T}}(1+\eta_{\mathrm{b}}\bar{n}_{\mathrm{T}})}}{1-\eta_{\mathrm{b}}} \times \frac{\eta_{\mathrm{b}}}{2(1-\eta_{\mathrm{b}})\bar{n}_{\mathrm{T}}+1}. \tag{51}$$

We note that (50) clearly demonstrates the square-root law governing the total number of covert bits M. The expression in (50) also confirms the linear dependence of M on the 'undetectability' parameter ϵ. Furthermore, the Taylor series expansion of the 'constant factor' C_{T} at $\bar{n}_{\mathrm{T}} = 0$ reveals that, when $\bar{n}_{\mathrm{T}} \ll 1$, the lower bound on M is approximately $\frac{\sqrt{n}\epsilon\eta_{\mathrm{b}}\sqrt{\eta_{\mathrm{b}}\bar{n}_{\mathrm{T}}}}{(1-\eta_{\mathrm{b}})\ln 2} - \log_2(\delta)$. Thus, covert communication benefits from increased noise power. Now, per discussion in the Supplementary Note 1, the total number of available spatio-temporal-polarization modes is $n = 2 \times n_{\mathrm{S}} \times n_{\mathrm{T}}$, where the number of spatial modes n_{S} decreases in the near-field regime as the center wavelength λ increases (since the Fresnel product $D_f \propto \frac{1}{\lambda^2}$). However, the noise power increases as the center wavelength increases. Figure 5 demonstrates that, at least up to 4 μm center wavelength, this increased noise power more than offsets the decrease in the number of available spatial modes. We also note that the channel transmissivity determines the covert communication volume in the highly noisy regime, since $\lim_{\bar{n}_{\mathrm{T}}\to\infty} C_{\mathrm{T}} = \frac{4\eta_{\mathrm{b}}^2}{(1-\eta_{\mathrm{b}})^2}$. Finally, while this analysis sheds light on the relationship between covert communication performance, system parameters ϵ and δ, and channel parameters η_{b} and $\bar{n}_{\mathrm{T}}$, the exact characterization of the maximum covert volume as a function of these parameters remains an open problem.

Before proving Theorems 3 and 4, we state a lemma that is used in their proofs.

Lemma 5 (Classical relative entropy bound on $\mathbb{P}_{\mathrm{e}}$ of binary hypothesis test)**.** *Denote by $\mathbb{P}_0$ and $\mathbb{P}_1$ the respective probability distributions of observations when H_0 and H_1 is true. Assuming equal prior probabilities for each hypothesis, the probability of discrimination error is $\mathbb{P}_{\mathrm{e}} \leq \frac{1}{2} - \sqrt{\frac{1}{8}D(\mathbb{P}_0\|\mathbb{P}_1)}$, where $D(\mathbb{P}_0\|\mathbb{P}_1) = -\sum_x p_0(x)\ln\frac{p_1(x)}{p_0(x)}$ is the classical relative entropy between $\mathbb{P}_0$ and $\mathbb{P}_1$ and $p_0(x)$ and $p_1(x)$ are the respective probability mass functions of $\mathbb{P}_0$ and $\mathbb{P}_1$.*

Proof. The minimum probability of discrimination error between H_0 and H_1 is characterized by [18, Theorem

13.1.1]:

$$\min \mathbb{P}_{\mathrm{e}} = \frac{1}{2} - \frac{1}{4}\|p_0(x) - p_1(x)\|_1,$$

where $\|a-b\|_1$ is the $\mathcal{L}_1$ norm. By classical Pinsker's inequality [19, Lemma 11.6.1],

$$\|p_0(x) - p_1(x)\|_1 \leq \sqrt{2D(\mathbb{P}_0\|\mathbb{P}_1)},$$

and the lemma follows. □

Theorem 3 (Dark counts yield square-root law)**.** *Suppose that Willie has a pure-loss channel from Alice, captures all photons transmitted by Alice that do not reach Bob, but is limited to a receiver with a non-zero dark current. Let Alice and Bob share a secret of sufficient length before communicating. Then Alice can lower-bound Willie's detection error probability* $\mathbb{P}_{\mathrm{e}}^{(\mathrm{w})} \geq \frac{1}{2} - \epsilon$ *for any* $\epsilon > 0$ *while reliably transmitting* $\mathcal{O}(\sqrt{n})$ *bits to Bob in* n *modes.*

Proof. Construction: Let Alice use a coherent state on-off keying (OOK) modulation $\{\pi_i, |\psi_i\rangle\langle\psi_i|\}$, $i = 1, 2$, where $\pi_1 = 1 - q$, $\pi_2 = q$, $|\psi_1\rangle = |0\rangle$, $|\psi_2\rangle = |\alpha\rangle$. Alice and Bob generate a random codebook with each codeword symbol chosen i.i.d. from the above binary OOK constellation.

Analysis (Willie): Willie records vector $\mathbf{y}_{\mathrm{w}} = [y_1, \ldots, y_n]$, where y_i is the number of photons observed in the ith mode. Denote by $\mathbb{P}_0$ the distribution of $\mathbf{y}_{\mathrm{w}}$ when Alice does not transmit and by $\mathbb{P}_1$ the distribution when she transmits. When Alice does not transmit, Willie's receiver observes a Poisson dark count process with rate λ_{w} photons per mode. Thus, $\{y_i\}$ is independent and identically distributed (i.i.d.) sequence of Poisson random variables with rate λ_{w}, and $\mathbb{P}_0 = \mathbb{P}_{\mathrm{w}}^n$ where $\mathbb{P}_{\mathrm{w}} = \text{Poisson}(\lambda_{\mathrm{w}})$. When Alice transmits, although Willie captures all of her transmitted energy that does not reach Bob, he does not have access to Alice's and Bob's codebook. Since the dark counts are independent of the transmitted pulses, each observation is a mixture of two independent Poisson random variables. Thus, each $y_i \sim \mathbb{P}_{\mathrm{s}}$ is i.i.d., with $\mathbb{P}_{\mathrm{s}} = (1-q)\text{Poisson}(\lambda_{\mathrm{w}}) + q\text{Poisson}(\lambda_{\mathrm{w}} + \eta_{\mathrm{w}}|\alpha|^2)$ and $\mathbb{P}_1 = \mathbb{P}_{\mathrm{s}}^n$. By Lemma 5, $\mathbb{P}_{\mathrm{e}}^{(\mathrm{w})} \geq \frac{1}{2} - \sqrt{\frac{1}{8}D(\mathbb{P}_0\|\mathbb{P}_1)}$. Since the classical relative entropy is additive for product distributions, $D(\mathbb{P}_0\|\mathbb{P}_1) = nD(\mathbb{P}_{\mathrm{w}}\|\mathbb{P}_{\mathrm{s}})$. Now,

$$\begin{aligned} D(\mathbb{P}_{\mathrm{w}}\|\mathbb{P}_{\mathrm{s}}) &= -\sum_{y=0}^{\infty} \frac{\lambda_{\mathrm{w}}^y e^{-\lambda_{\mathrm{w}}}}{y!} \log\left[1 - q + q\left(1 + \frac{\eta_{\mathrm{w}}|\alpha|^2}{\lambda_{\mathrm{w}}}\right)e^{-\eta_{\mathrm{w}}|\alpha|^2}\right] \\ &\leq \frac{q^2\left(e^{(\eta_{\mathrm{w}}|\alpha|^2)^2/\lambda_{\mathrm{w}}} - 1\right)}{2} \end{aligned} \tag{52}$$

where the inequality is from the application of the Taylor's Theorem with the remainder applied to the Taylor series expansion of equation (52) with respect to q at $q = 0$. Thus,

$$\mathbb{P}_{\mathrm{e}}^{(\mathrm{w})} \geq \frac{1}{2} - \frac{q}{4}\sqrt{n\left(e^{(\eta_{\mathrm{w}}|\alpha|^2)^2/\lambda_{\mathrm{w}}} - 1\right)}. \tag{53}$$

Therefore, to ensure that $\mathbb{P}_{\mathrm{e}}^{(\mathrm{w})} \geq \frac{1}{2} - \epsilon$, Alice sets

$$q = \frac{4\epsilon}{\sqrt{n\left(e^{(\eta_{\mathrm{w}}|\alpha|^2)^2/\lambda_{\mathrm{w}}} - 1\right)}}. \tag{54}$$

Analysis (Bob): Suppose Bob uses a practical single photon detector (SPD) receiver with probability of a dark click per mode $p_{\mathrm{D}}^{(\mathrm{b})}$. This induces a binary asymmetric channel between Alice and Bob, where the click probabilities, conditional on the input, are $\mathbb{P}(\text{click} \mid \text{input } |0\rangle) = p_{\mathrm{D}}^{(\mathrm{b})}$ and $\mathbb{P}(\text{click} \mid \text{input } |\alpha\rangle) = 1 - e^{-\eta_{\mathrm{b}}|\alpha|^2}(1 - p_{\mathrm{D}}^{(\mathrm{b})})$, with the corresponding no-click probabilities $\mathbb{P}(\text{no-click} \mid \text{input } |0\rangle) = 1 - p_{\mathrm{D}}^{(\mathrm{b})}$ and $\mathbb{P}(\text{no-click} \mid \text{input } |\alpha\rangle) = e^{-\eta_{\mathrm{b}}|\alpha|^2}(1 - p_{\mathrm{D}}^{(\mathrm{b})})$. At each mode, a click corresponds to "1" and no-click to "0".

Let Bob use a maximum likelihood decoder on this sequence. We use [20, Theorem 5.6.2] with parameter $\rho = 1$ to upper-bound Bob's average decoding error probability $\mathbb{P}_{\mathrm{e}}^{(\mathrm{b})} \leq e^{M - nE_0}$, where E_0 is:

$$E_0 = -\ln\left[\left(1 - p_{\mathrm{D}}^{(\mathrm{b})}\right)\left(1 - q\left(1 - e^{-\frac{\eta|\alpha|^2}{2}}\right)\right)^2 + \left((1-q)\sqrt{p_{\mathrm{D}}^{(\mathrm{b})}} + q\sqrt{1 - \left(1 - p_{\mathrm{D}}^{(\mathrm{b})}\right)e^{-\eta|\alpha|^2}}\right)^2\right]$$

The Taylor series expansion of E_0 with respect to q at $q = 0$ yields $E_0 = qC_{\mathrm{D}} + \mathcal{O}(q^2)$, where

$$C_{\mathrm{D}} = 2e^{-\eta|\alpha|^2/2}\left(e^{\eta|\alpha|^2/2} - 1 + p_{\mathrm{D}}^{(\mathrm{b})} - \sqrt{p_{\mathrm{D}}^{(\mathrm{b})}\left(e^{\eta|\alpha|^2/2} - 1 + p_{\mathrm{D}}^{(\mathrm{b})}\right)}\right)$$

is a positive constant. Since $q = \mathcal{O}(1/\sqrt{n})$, this demonstrates that $\mathcal{O}(\sqrt{n})$ bits can be covertly transmitted from Alice to Bob with $\mathbb{P}_{\mathrm{e}}^{(\mathrm{b})} < \delta$ for arbitrary $\delta > 0$ given large enough n. □

Theorem 4 (Dark counts yield square-root law under structured modulation)**.** *Suppose that Willie has a pure-loss channel from Alice, can capture all photons transmitted by Alice that do not reach Bob, but is limited to a PNR receiver with a non-zero dark current. Let Alice and Bob share a secret of sufficient length before communicating. Then Alice can lower-bound Willie's detection error probability* $\mathbb{P}_{\mathrm{e}}^{(\mathrm{w})} \geq \frac{1}{2} - \epsilon$ *for any* $\epsilon > 0$ *while reliably transmitting* $\mathcal{O}(\sqrt{\frac{n}{Q}}\log Q)$ *bits to Bob using* n *modes and a* Q*-ary PPM constellation.*

Proof. Construction: Prior to communication, Alice and Bob secretly choose a random subset $\mathcal{S}$ of PPM frames to use for transmission by selecting each of n/Q available PPM frames independently with probability ζ. Alice and Bob then secretly generate a vector $\mathbf{k}$ containing $|\mathcal{S}|$ numbers selected independently uniformly at random from $\{0, 1, \ldots, Q-1\}$, where $|\mathcal{S}|$ denotes the cardinality of $\mathcal{S}$. Alice encodes a message into a codeword of size $|\mathcal{S}|$ using an ECC that may be known to Willie. She adds $\mathbf{k}$ modulo Q to this message and transmits it on the PPM frames in $\mathcal{S}$.

Analysis (Willie): Willie detects each PPM frame received from Alice, recording the photon counts in $\mathbf{y}_{\mathrm{w}} = [\mathbf{y}_1^{(\mathrm{w})}, \ldots, \mathbf{y}_n^{(\mathrm{w})}]$ where $\mathbf{y}_i^{(\mathrm{w})} = [y_{i,1}^{(\mathrm{w})}, \ldots, y_{i,Q}^{(\mathrm{w})}]$ and $y_{i,j}^{(\mathrm{w})}$ it the number of photons observed in the jth mode of the ith PPM frame. Denote by $\mathbb{P}_0$ the distribution of $\mathbf{y}_{\mathrm{w}}$ when Alice does not transmit and by $\mathbb{P}_1$ the distribution when she transmits. When Alice does not transmit, Willie's receiver observes a Poisson dark count process with rate λ_{w} photons per mode, implying that $\mathbf{y}_{\mathrm{w}}$ is a vector of nQ i.i.d. Poisson(λ_{w}) random variables. Therefore, $\{\mathbf{y}_i^{(\mathrm{w})}\}$ is i.i.d. with $\mathbf{y}_i^{(\mathrm{w})} \sim \mathbb{P}_{\mathrm{w}}$ and $\mathbb{P}_0 = \mathbb{P}_{\mathrm{w}}^n$, where $\mathbb{P}_{\mathrm{w}}$ is the distribution of Q i.i.d. Poisson(λ_{w}) random variables with p.m.f.:

$$p_0(\mathbf{y}_i^{(\mathrm{w})}) = \prod_{j=1}^{Q} \frac{\lambda_{\mathrm{w}}^{y_{i,j}^{(\mathrm{w})}} e^{-\lambda_{\mathrm{w}}}}{y_{i,j}^{(\mathrm{w})}!}. \tag{55}$$

When Alice transmits, by construction, each PPM frame is randomly selected for transmission with probability ζ. In each selected PPM frame, a pulse is transmitted using one of Q modes chosen equiprobably. Therefore, in this case $\{\mathbf{y}_i^{(\mathrm{w})}\}$ is also i.i.d. with $\mathbf{y}_i^{(\mathrm{w})} \sim \mathbb{P}_{\mathrm{s}}$ and $\mathbb{P}_1 = \mathbb{P}_{\mathrm{s}}^n$, where the p.m.f. of $\mathbb{P}_{\mathrm{s}}$ is:

$$p_1(\mathbf{y}_i^{(\mathrm{w})}) = (1-\zeta)\prod_{j=1}^{Q} \frac{\lambda_{\mathrm{w}}^{y_{i,j}^{(\mathrm{w})}} e^{-\lambda_{\mathrm{w}}}}{y_{i,j}^{(\mathrm{w})}!} + \frac{\zeta}{Q}\sum_{m=1}^{Q} \frac{(\eta_{\mathrm{w}}|\alpha|^2 + \lambda_{\mathrm{w}})^{y_{i,m}^{(\mathrm{w})}} e^{-\eta_{\mathrm{w}}|\alpha|^2 - \lambda_{\mathrm{w}}}}{y_{i,m}^{(\mathrm{w})}!} \prod_{\substack{j=1 \\ j \neq m}}^{Q} \frac{\lambda_{\mathrm{w}}^{y_{i,j}^{(\mathrm{w})}} e^{-\lambda_{\mathrm{w}}}}{y_{i,j}^{(\mathrm{w})}!}. \tag{56}$$

Since the classical relative entropy is additive for product distributions, $D(\mathbb{P}_0\|\mathbb{P}_1) = \frac{n}{Q}D(\mathbb{P}_{\mathrm{w}}\|\mathbb{P}_{\mathrm{s}})$. Now,

denoting by $\mathbf{x} = [x_1, \cdots, x_Q]$ where $x_j \in \mathbb{N}_0$, we have:

$$D(\mathbb{P}_\mathrm{w}\|\mathbb{P}_\mathrm{s}) = -\sum_{\mathbf{x}\in\mathbb{N}_0^Q}\prod_{j=1}^{Q}\frac{\lambda_\mathrm{w}^{x_j}e^{-\lambda_\mathrm{w}}}{x_j!}\log\left[1-\zeta+\frac{\zeta}{Q}\sum_{m=1}^{Q}\left(1+\frac{\eta_\mathrm{w}|\alpha|^2}{\lambda_\mathrm{w}}\right)^{x_m}e^{-\eta_\mathrm{w}|\alpha|^2}\right] \\ \leq \frac{\zeta^2\left(e^{(\eta_\mathrm{w}|\alpha|^2)^2/\lambda_\mathrm{w}}-1\right)}{2Q} \tag{57}$$

where the inequality is from the application of the Taylor's Theorem with the remainder applied to the Taylor series expansion of equation (57) with respect to ζ at $\zeta = 0$. By Lemma 5, $\zeta = \frac{4\epsilon Q}{\sqrt{n\left(e^{(\eta_\mathrm{w}|\alpha|^2)^2/\lambda_\mathrm{w}}-1\right)}}$ ensures that Willie's error probability is lower-bounded by $\mathbb{P}_\mathrm{e}^{(\mathrm{w})} \geq \frac{1}{2} - \epsilon$.

Analysis (Bob): As in the proof of Theorem 3, Bob uses a practical SPD receiver with probability of a dark click $p_\mathrm{D}^{(\mathrm{b})}$. Bob examines only the PPM frames in $\mathcal{S}$. If two or more clicks are detected in a PPM frame, a PPM symbol is assigned by selecting one of the clicks uniformly at random. If no clicks are detected, the PPM frame is labeled as an erasure. After subtracting $\mathbf{k}$ modulo Q from this vector of PPM symbols (subtraction is not performed on erasures), the resultant vector is passed to the decoder. A random coding argument [20, Theorem 5.6.2] yields reliable transmission of $\mathcal{O}\left(\sqrt{\frac{n}{Q}}\log Q\right)$ covert bits. □

Theorem 5 (Converse of the square-root law)**.** *Suppose Alice only uses n-mode codewords with total photon number variance $\sigma_x^2 = \mathcal{O}(n)$. Then, if she attempts to transmit $\omega(\sqrt{n})$ bits in n modes, as $n \to \infty$, she is either detected by Willie with arbitrarily low detection error probability, or Bob cannot decode with arbitrarily low decoding error probability.*

Proof. As in the proof of Theorem 1, Alice sends one of 2^M (equally likely) M-bit messages by choosing an element from an arbitrary codebook $\{\hat{\rho}_x^{A^n}, x = 1, \ldots, 2^M\}$, where a state $\hat{\rho}_x^{A^n} = |\psi_x\rangle^{A^n A^n}\langle\psi_x|$ encodes an M-bit message W_x. $|\psi_x\rangle^{A^n} = \sum_{\mathbf{k}\in\mathbb{N}_0^n} a_\mathbf{k}(x)\,|\mathbf{k}\rangle$ is a general n-mode pure state, where $|\mathbf{k}\rangle \equiv |k_1\rangle \otimes |k_2\rangle \otimes \cdots \otimes |k_n\rangle$ is a tensor product of n Fock states. The mean photon number of a codeword $\hat{\rho}_x^{A^n}$ is $\bar{n}_x = \sum_{\mathbf{k}\in\mathbb{N}_0^n}(\sum_{i=1}^n k_i)|a_\mathbf{k}(x)|^2$, and the photon number variance is $\sigma_x^2 = \sum_{\mathbf{k}\in\mathbb{N}_0^n}(\sum_{i=1}^n k_i)^2|a_\mathbf{k}(x)|^2 - \bar{n}_x^2 = \mathcal{O}(n)$. We limit our analysis to pure input states since, by convexity, using mixed states as inputs can only deteriorate the performance (since that is equivalent to transmitting a randomly chosen pure state from an ensemble and discarding the knowledge of that choice).

Willie uses a noisy PNR receiver to observe his channel from Alice, and records the total photon count X_tot over n modes. For some threshold S that we discuss later, Willie declares that Alice transmitted when $X_\mathrm{tot} \geq S$, and did not transmit when $X_\mathrm{tot} < S$. When Alice does not transmit, Willie observes noise: $X_\mathrm{tot}^{(0)} = X_\mathrm{D} + X_\mathrm{T}$, where X_D is the number of dark counts from the spontaneous emission process at the detector, and X_T is the number of photons observed from the thermal background. Since the dark counts are modeled by a Poisson process with rate λ_w photons per mode, both the mean and variance of the observed dark counts per mode is λ_w. The mean of the number of photons observed per mode from the thermal background with mean photon number per mode $\bar{n}_\mathrm{T}$ is $(1-\eta_\mathrm{w})\bar{n}_\mathrm{T}$ and the variance is $(1-\eta_\mathrm{w})^2(\bar{n}_\mathrm{T}+\bar{n}_\mathrm{T}^2)$. Thus, the mean of the total number of noise photons observed per mode is $\mu_\mathrm{N} = \lambda_\mathrm{w} + (1-\eta_\mathrm{w})\bar{n}_\mathrm{T}$, and, because of the statistical independence of the noise processes, the variance is $\sigma_\mathrm{N}^2 = \lambda_\mathrm{w} + (1-\eta_\mathrm{w})^2(\bar{n}_\mathrm{T}+\bar{n}_\mathrm{T}^2)$. We upper-bound the false alarm probability using Chebyshev's inequality:

$$\mathbb{P}_\mathrm{FA} = \mathbb{P}(X_\mathrm{tot}^{(0)} \geq S) \\ \leq \frac{n\sigma_\mathrm{N}^2}{(S-n\mu_\mathrm{N})^2}, \tag{58}$$

where equation (58) is because of the memorylessness of the noise processes. Thus, to obtain the desired $\mathbb{P}_\mathrm{FA}^*$, Willie sets threshold $S = n\mu_\mathrm{N} + \sqrt{n\sigma_\mathrm{N}^2/\mathbb{P}_\mathrm{FA}^*}$.

When Alice transmits codeword $\hat{\rho}_u^{A^n}$ corresponding to message W_u, Willie observes $X_{\text{tot}}^{(1)} = X_u + X_{\text{D}} + X_{\text{T}}$, where X_u is the count from Alice's transmission. We upper-bound the missed detection probability using Chebyshev's inequality:

$$\begin{aligned}\mathbb{P}_{\text{MD}} &= \mathbb{P}(X_{\text{tot}}^{(1)} < S) \\ &\leq \mathbb{P}\left(|X_{\text{tot}}^{(1)} - \eta_{\text{w}}\bar{n}_u - n\mu_{\text{N}}| \geq \eta_{\text{w}}\bar{n}_u - \sqrt{\frac{n\sigma_{\text{N}}^2}{\mathbb{P}_{\text{FA}}^*}}\right) \\ &\leq \frac{n\sigma_{\text{N}}^2 + \eta_{\text{w}}^2\sigma_u^2}{(\eta_{\text{w}}\bar{n}_u - \sqrt{n\sigma_{\text{N}}^2/\mathbb{P}_{\text{FA}}^*})^2}, \end{aligned} \tag{59}$$

where equation (59) is because the noise and Alice's codeword are independent. Since $\sigma_u^2 = \mathcal{O}(n)$, if $\bar{n}_u = \omega(\sqrt{n})$, then $\lim_{n\to\infty}\mathbb{P}_{\text{MD}} = 0$. Thus, given large enough n, Willie can detect Alice's codewords that have mean photon number $\bar{n}_u = \omega(\sqrt{n})$ with probability of error $\mathbb{P}_{\text{e}}^{(\text{w})} \leq \epsilon$ for any $\epsilon > 0$.

Now, if Alice wants to lower-bound $\mathbb{P}_{\text{e}}^{(\text{w})}$, her codebook must contain a positive fraction of codewords with mean photon number upper-bounded by $\bar{n}_{\mathcal{U}} = \mathcal{O}(\sqrt{n})$. Formally, there must exist a subset of the codebook $\left\{\hat{\rho}_u^{A^n}, u \in \mathcal{U}\right\}$, where $\mathcal{U} = \{u : \bar{n}_u \leq \bar{n}_{\mathcal{U}}\}$, with $\frac{|\mathcal{U}|}{2^M} \geq \kappa$ and $\kappa > 0$. Suppose Bob has an unattenuated pure-loss channel from Alice ($\eta_{\text{b}} = 0$ and $\bar{n}_{\text{T}} = 0$) and access to any receiver allowed by quantum mechanics. The decoding error probability $\mathbb{P}_{\text{e}}^{(\text{b})}$ in such scenario clearly lower-bounds the decoding error probability in a practical scenario where the channel from Alice is lossy and either the channel or the receiver are noisy. Denote by $E_{a\to k}$ the event that a transmitted message W_a is decoded as $W_k \neq W_a$. Since the messages are equiprobable, the average probability of error for the codebook containing only the codewords in $\mathcal{U}$ is:

$$\mathbb{P}_{\text{e}}^{(\text{b})}(\mathcal{U}) = \frac{1}{|\mathcal{U}|}\sum_{a\in\mathcal{U}}\mathbb{P}\left(\cup_{k\in\mathcal{U}\setminus\{a\}}E_{a\to k}\right). \tag{60}$$

Since the probability that a message is sent from $\mathcal{U}$ is κ,

$$\mathbb{P}_{\text{e}}^{(\text{b})} \geq \kappa\mathbb{P}_{\text{e}}^{(\text{b})}(\mathcal{U}). \tag{61}$$

Equality holds only when Bob receives messages that are not in $\mathcal{U}$ error-free and knows when the messages from $\mathcal{U}$ are sent (in other words, equality holds when the set of messages on which decoder can err is reduced to $\mathcal{U}$). Denote by W_a, $a \in \mathcal{U}$, the message transmitted by Alice, and by $\hat{W}_a$ Bob's decoding of W_a. Then, since each message is equiprobable and $|\mathcal{U}| = \kappa 2^M$,

$$\begin{aligned}\log_2\kappa + M &= H(W_a) &(62)\\ &= I(W_a;\hat{W}_a) + H(W_a|\hat{W}_a) &(63)\\ &\leq I(W_a;\hat{W}_a) + 1 + (\log_2\kappa + M)\mathbb{P}_{\text{e}}^{(\text{b})}(\mathcal{U}) &(64)\\ &\leq \chi\left(\left\{\frac{1}{|\mathcal{U}|}, \hat{\rho}_u^{A^n}\right\}\right) + 1 + (\log_2\kappa + M)\mathbb{P}_{\text{e}}^{(\text{b})}(\mathcal{U}) &(65)\end{aligned}$$

where (63) is from the definition of mutual information, (64) is because of classical Fano's inequality [19, Equation (9.37)], and (65) is the Holevo bound $I(X;Y) \leq \chi(\{p_X(x), \hat{\rho}_x\})$ [21]. The mutual information $I(X;Y)$ is between a classical input X and a classical output Y, which is a function of the prior probability distribution $p_X(x)$, and the conditional probability distribution $p_{Y|X}(y|x)$, with $x \in \mathcal{X}$ and $y \in \mathcal{Y}$. The classical input x maps to a quantum state $\hat{\rho}_x$. A specific choice of a quantum measurement, described by POVM elements $\{\Pi_y, y \in \mathcal{Y}\}$, induces the conditional probability distribution $p_{Y|X}(y|x) = \text{Tr}\left[\Pi_y\hat{\rho}_x\right]$. The Holevo information, $\chi(\{p_x, \hat{\rho}_x\}) = S\left(\sum_{x\in\mathcal{X}}p_x\hat{\rho}_x\right) - \sum_{x\in\mathcal{X}}p_x S(\hat{\rho}_x)$, where $S(\hat{\rho}) \equiv -\text{Tr}[\hat{\rho}\ln\hat{\rho}]$ is the von Neumann entropy of the state $\hat{\rho}$, is not a function of the quantum measurement. Since $\hat{\rho}_u^{A^n} = |\psi_u\rangle^{A^n A^n}\langle\psi_u|$ is a pure state, $\chi\left(\{\frac{1}{|\mathcal{U}|}, \hat{\rho}_u^{A^n}\}\right) = S\left(\frac{1}{|\mathcal{U}|}\sum_{u\in\mathcal{U}}|\psi_u\rangle^{A^n A^n}\langle\psi_u|\right)$. Denote the "average codeword" in $\mathcal{U}$ by

$\bar{\rho}^{A^n}=\frac{1}{|\mathcal{U}|}\sum_{u\in\mathcal{U}}|\psi_u\rangle^{A^nA^n}\langle\psi_u|$, and the state of the jth mode of $\bar{\rho}^{A^n}$ by $\bar{\rho}_j^{A^n}$. We obtain $\bar{\rho}_j^{A^n}$ by tracing out all the other modes in $\bar{\rho}^{A^n}$ and denote its mean photon number by $\bar{n}_j$ (that is, $\bar{n}_j$ is the mean photon number of the jth mode of $\bar{\rho}^{A^n}$). Finally, denote a coherent state ensemble with a zero-mean circularly-symmetric Gaussian distribution by $\hat{\rho}_{\bar{n}}^T=\frac{1}{\pi\bar{n}}\int e^{-|\alpha|^2/\bar{n}}|\alpha\rangle\langle\alpha|\mathrm{d}^2\alpha$. The von Neumann entropy of $\hat{\rho}_{\bar{n}}^T$, $S\left(\hat{\rho}_{\bar{n}}^T\right)=\log_2(1+\bar{n})+\bar{n}\log_2\left(1+\frac{1}{\bar{n}}\right)$. Now,

$$S\left(\bar{\rho}^{A^n}\right)\leq\sum_{j=1}^{n}S\left(\bar{\rho}_j^{A^n}\right) \tag{66}$$

$$\leq\sum_{i=1}^{n}\log_2(1+\bar{n}_j)+\bar{n}_j\log_2\left(1+\frac{1}{\bar{n}_j}\right) \tag{67}$$

$$\leq n\left(\log_2\left(1+\frac{\bar{n}_{\mathcal{U}}}{n}\right)+\frac{\bar{n}_{\mathcal{U}}}{n}\log_2\left(1+\frac{n}{\bar{n}_{\mathcal{U}}}\right)\right), \tag{68}$$

where (66) follows from the sub-additivity of the von Neumann entropy and (67) is because $\hat{\rho}_{\bar{n}}^T$ maximizes the von Neumann entropy of a single-mode state with mean photon number constraint $\bar{n}$ [15]. Now, $S\left(\hat{\rho}_{\bar{n}}^T\right)$ is concave and increasing for $\bar{n}>0$, and, since $\sum_{j=1}^{n}\bar{n}_j\leq\bar{n}_{\mathcal{U}}$ by construction of $\mathcal{U}$, the application of Jensen's inequality yields (68). Combining (65) and (68) and solving for $\mathbb{P}_{\mathrm{e}}^{(\mathrm{b})}(\mathcal{U})$ yields:

$$\mathbb{P}_{\mathrm{e}}^{(\mathrm{b})}(\mathcal{U})\geq 1-\frac{\log_2\left(1+\frac{\bar{n}_{\mathcal{U}}}{n}\right)+\frac{\bar{n}_{\mathcal{U}}}{n}\log_2\left(1+\frac{n}{\bar{n}_{\mathcal{U}}}\right)+\frac{1}{n}}{\frac{\log_2\kappa}{n}+\frac{M}{n}}. \tag{69}$$

Substituting (69) into (61) yields the following lower bound on Bob's decoding error probability:

$$\mathbb{P}_{\mathrm{e}}^{(\mathrm{b})}\geq\kappa\left[1-\frac{\log_2\left(1+\frac{\bar{n}_{\mathcal{U}}}{n}\right)+\frac{\bar{n}_{\mathcal{U}}}{n}\log_2\left(1+\frac{n}{\bar{n}_{\mathcal{U}}}\right)+\frac{1}{n}}{\frac{\log_2\kappa}{n}+\frac{M}{n}}\right] \tag{70}$$

Since Alice transmits $\omega(\sqrt{n})$ bits in n modes, $M/n=\omega(1/\sqrt{n})$ bits/symbol. However, since $\bar{n}_{\mathcal{U}}=\mathcal{O}(\sqrt{n})$, as $n\to\infty$, $\mathbb{P}_{\mathrm{e}}^{(\mathrm{b})}$ is bounded away from zero for any $\kappa>0$. Thus, Alice cannot transmit $\omega(\sqrt{n})$ bits in n modes both covertly and reliably. □

Supplementary Note 4. Calculation of the Maximum Number of Covert Bits Received by Bob

The Q-ary PPM signaling combined with Bob's device for assigning symbols to received PPM frames induces a discrete memoryless channel described by a conditional distribution $\mathbb{P}(Y|X)$, where $X\in\{1,\ldots,Q\}$ is Alice's input symbol and $Y\in\{1,\ldots,Q,\mathcal{E}\}$ is Bob's output symbol with $\mathcal{E}$ indicating an erasure. Since Bob observes Alice's pulse with probability $1-e^{-\bar{n}_{\mathrm{det}}^{(\mathrm{b})}}$, $\mathbb{P}(Y|X)$ is characterized as follows:

$$\mathbb{P}(Y=x|X=x)=\left(1-e^{-\bar{n}_{\mathrm{det}}^{(\mathrm{b})}}\right)\sum_{i=0}^{Q-1}\frac{1}{i+1}\left(p_{\mathrm{D}}^{(\mathrm{b})}\right)^i\left(1-p_{\mathrm{D}}^{(\mathrm{b})}\right)^{Q-1-i}+e^{-\bar{n}_{\mathrm{det}}^{(\mathrm{b})}}\sum_{i=1}^{Q}\frac{1}{i}\left(p_{\mathrm{D}}^{(\mathrm{b})}\right)^i\left(1-p_{\mathrm{D}}^{(\mathrm{b})}\right)^{Q-i}$$

$$\mathbb{P}(Y=\mathcal{E}|X=x)=e^{-\bar{n}_{\mathrm{det}}^{(\mathrm{b})}}\left(1-p_{\mathrm{D}}^{(\mathrm{b})}\right)^Q$$

$$\mathbb{P}(Y=y,y\notin\{x,\mathcal{E}\}|X=x)=\frac{1-\mathbb{P}(Y=x|x)-\mathbb{P}(Y=\mathcal{E}|x)}{Q-1}$$

The symmetry of this channel allows straightforward computation of its Shannon capacity [22] $C_{\mathrm{s}}=I(X;Y)$, where $\mathbb{P}(X=x)=\frac{1}{Q}$ for $x=1,\ldots,Q$ and $I(X;Y)$ is the mutual information between X and Y. We use the estimates from Table I to compute C_{s} for each regime, and plot $\frac{C_{\mathrm{s}}\zeta n}{Q}$ in Figure 3 since $\frac{\zeta n}{Q}$ is the expected number of PPM frames selected for transmission.

Supplementary Note 5. Mathematical Details of Willie's Hypothesis Test

Willie collects the click record: a binary sequence $\mathbf{x}_\mathrm{w} = \left\{x_j^{(\mathrm{w})}\right\}_{j=1}^n$, $x_j^{(\mathrm{w})} \in \{0,1\}$ of his single photon detector's (SPD's) observations of the channel from Alice, where "0" and "1" indicate the absence and the presence of a click, respectively. The hypothesis test on Alice's transmission state is between two point hypotheses, as Alice is either not transmitting (H_0) or transmitting (H_1). Thus, the log-likelihood ratio test minimizes Willie's detection error probability $\mathbb{P}_\mathrm{e}^{(\mathrm{w})}$ [18, Theorem 13.1.1]. Here we derive Willie's test statistic.

The log-likelihood ratio is $L = \ln \frac{f_1(\mathbf{x}_\mathrm{w})}{f_0(\mathbf{x}_\mathrm{w})}$, where $f_0(\mathbf{x}_\mathrm{w})$ and $f_1(\mathbf{x}_\mathrm{w})$ are the likelihood functions of the click record $\mathbf{x}_\mathrm{w}$ corresponding respectively to Alice being quiet and transmitting. When Alice does not transmit, Willie only observes dark clicks. Suppose that the dead time of Willie's SPD $t_\mathrm{d} = 0$. Then Willie's detector readings are independent, and $\mathbf{x}_\mathrm{w}$ is a vector of i.i.d. Bernoulli $\left(p_\mathrm{D}^{(\mathrm{w})}\right)$ random variables. The likelihood function of $\mathbf{x}_\mathrm{w}$ under H_0 is then:

$$f_0(\mathbf{x}_\mathrm{w}) = \left(p_\mathrm{D}^{(\mathrm{w})}\right)^{\sum_{j=1}^n x_j^{(\mathrm{w})}} \left(1 - p_\mathrm{D}^{(\mathrm{w})}\right)^{\sum_{j=1}^n 1 - x_j^{(\mathrm{w})}} \tag{71}$$

$$= \prod_{i=1}^{n/Q} \left(p_\mathrm{D}^{(\mathrm{w})}\right)^{\sum_{j=1}^Q x_{l_{ij}}^{(\mathrm{w})}} \left(1 - p_\mathrm{D}^{(\mathrm{w})}\right)^{\sum_{j=1}^Q 1 - x_{l_{ij}}^{(\mathrm{w})}}, \tag{72}$$

where $l_{ij} = (i-1)Q + j$ and in (72) we evaluate each PPM symbol separately, as that would be convenient later.

When the dead time of the detectors $t_\mathrm{d} > 0$, as is the case for our SPDs, the observations are not completely independent. Since a click is always followed by t_d observations without any clicks, each occurrence of "1" in $\mathbf{x}_\mathrm{w}$ is followed by t_d occurrences of "0" with probability one. Thus, the likelihood function given in (72) has to be adjusted as follows:

$$f_0(\mathbf{x}_\mathrm{w}) = \prod_{i=1}^{n/Q} \left(p_\mathrm{D}^{(\mathrm{w})}\right)^{\sum_{j=1}^Q x_{l_{ij}}^{(\mathrm{w})}} \left(1 - p_\mathrm{D}^{(\mathrm{w})}\right)^{\sum_{j=1}^Q \bar{\bar{x}}_{l_{ij}}^{(\mathrm{w})}}, \tag{73}$$

where $\bar{\bar{x}}_{l_{ij}}^{(\mathrm{w})} = (1 - x_{l_{ij}}^{(\mathrm{w})})(1 - \sum_{k=1}^{t_\mathrm{d}} x_{l_{ij}-k})$, and the sum $\sum_{k=1}^{t_\mathrm{d}} x_{l_{ij}-k} \in \{0,1\}$ for $i = 1, \ldots, n/Q$ and $j = 1, \ldots, Q$, since the clicks are at least t_d observations apart. We note that, adopting the convention $\sum_{i=1}^0 f(i) = 0$, equations (72) and (73) are identical when $t_\mathrm{d} = 0$.

Now consider the scenario when Alice transmits. Again, we first derive the likelihood function assuming the dead time $t_\mathrm{d} = 0$, and then adjust for $t_\mathrm{d} > 0$. The secret shared between Alice and Bob identifies the random subset $\mathcal{S}$ of the PPM frames used for transmission, and a random vector $\mathbf{k}$ which is modulo-added to the codeword. Modulo addition of $\mathbf{k}$ effectively selects a random pulse location within each PPM frame. Note that, while both the construction in the proof of Theorem 4 and Alice's encoder described in the Methods generate $\mathcal{S}$ first and then $\mathbf{k}$, the order of these operations can be reversed: we can first fix a random location of a pulse in each of n/Q PPM frames, and then select a random subset of these frames. Consider Willie's observation of the ith PPM frame, and assume that the mth mode is used if the frame is selected for transmission. Denote the probability of Willie's detector observing Alice's pulse by $p_\mathrm{r}^{(\mathrm{w})} = 1 - e^{-\bar{n}_\mathrm{det}^{(\mathrm{w})}}$. By construction, frames are selected for transmission independent of each other with probability ζ. Willie's detector registers a click on the mth mode of the ith PPM frame when one of the following disjoint events occurs:

- The ith PPM frame is selected and pulse is detected by Willie in the mth mode of this frame (probability $\zeta p_\mathrm{r}^{(\mathrm{w})}$);
- The ith PPM frame is selected, but Willie, instead of detecting the pulse, records a dark click in the mth mode of this frame (probability $\zeta \left(1 - p_\mathrm{r}^{(\mathrm{w})}\right) p_\mathrm{D}^{(\mathrm{w})}$); and,

- Even though the ith PPM frame is not selected, Willie records a dark click in the mth mode of this frame (probability $(1-\zeta)p_{\text{D}}^{(\text{w})}$).

The probability of the union of these events is

$$p_{\text{s}}^{(\text{w})} = \zeta p_{\text{r}}^{(\text{w})}\left(1-p_{\text{D}}^{(\text{w})}\right) + p_{\text{D}}^{(\text{w})}. \tag{74}$$

Therefore, assuming detector dead time $t_{\text{d}} = 0$, Willie observes an independent Bernoulli $\left(p_{\text{s}}^{(\text{w})}\right)$ random variable in the mth mode of the ith PPM frame. Since Alice only uses the mth mode for transmission, in modes other than the mth, Willie observes a set of $Q-1$ i.i.d. Bernoulli $\left(p_{\text{D}}^{(\text{w})}\right)$ random variables corresponding to dark clicks, again, assuming $t_{\text{d}} = 0$. Thus, the likelihood function of $\mathbf{x}_{\text{w}}$ under H_1 when $t_{\text{d}} = 0$ is

$$f_1(\mathbf{x}_{\text{w}}) = \prod_{i=1}^{n/Q}\frac{1}{Q}\sum_{m=1}^{Q}\left(p_{\text{s}}^{(\text{w})}\right)^{x_{l_{im}}^{(\text{w})}}\left(1-p_{\text{s}}^{(\text{w})}\right)^{1-x_{l_{im}}^{(\text{w})}}\left(p_{\text{D}}^{(\text{w})}\right)^{\sum_{\substack{j=1\\ j\neq l}}^{Q} x_{l_{ij}}^{(\text{w})}}\left(1-p_{\text{D}}^{(\text{w})}\right)^{\sum_{\substack{j=1\\ j\neq l}}^{Q} 1-x_{l_{ij}}^{(\text{w})}},$$

where, as before, $l_{ij} = (i-1)Q + j$. Adjustment for $t_{\text{d}} > 0$ yields

$$f_1(\mathbf{x}_{\text{w}}) = \prod_{i=1}^{n/Q}\frac{1}{Q}\sum_{m=1}^{Q}\left(p_{\text{s}}^{(\text{w})}\right)^{x_{l_{im}}^{(\text{w})}}\left(1-p_{\text{s}}^{(\text{w})}\right)^{\overline{\overline{x}}_{l_{im}}^{(\text{w})}}\left(p_{\text{D}}^{(\text{w})}\right)^{\sum_{\substack{j=1\\ j\neq l}}^{Q} x_{l_{ij}}^{(\text{w})}}\left(1-p_{\text{D}}^{(\text{w})}\right)^{\sum_{\substack{j=1\\ j\neq l}}^{Q} \overline{\overline{x}}_{l_{ij}}^{(\text{w})}},$$

where, as before, $\overline{\overline{x}}_{l_{ij}}^{(\text{w})} = (1-x_{l_{ij}}^{(\text{w})})(1-\sum_{k=1}^{t_{\text{d}}} x_{l_{ij}-k})$.

The likelihood ratio is

$$\frac{f_1(\mathbf{x}_{\text{w}})}{f_0(\mathbf{x}_{\text{w}})} = \prod_{i=1}^{n/Q}\frac{1}{Q}\sum_{m=1}^{Q}\left(\frac{p_{\text{s}}^{(\text{w})}}{p_{\text{D}}^{(\text{w})}}\right)^{x_{l_{im}}^{(\text{w})}}\left(\frac{1-p_{\text{s}}^{(\text{w})}}{1-p_{\text{D}}^{(\text{w})}}\right)^{(1-x_{l_{im}}^{(\text{w})})(1-\sum_{k=1}^{t_{\text{d}}} x_{l_{im}-k})}. \tag{75}$$

Now, when $x_{l_{im}}^{(\text{w})} = 1$, the corresponding summation term in (75) is $\frac{p_{\text{s}}^{(\text{w})}}{p_{\text{D}}^{(\text{w})}}$. When $x_{l_{im}}^{(\text{w})} = 0$ and the no-click event is within the detector dead time (that is, $1-\sum_{k=1}^{t_{\text{d}}} x_{l_{im}-k} = 0$), then the corresponding summation term is one; otherwise the corresponding summation term is $\frac{1-p_{\text{s}}^{(\text{w})}}{1-p_{\text{D}}^{(\text{w})}}$. Denote by $y_i^{(\text{w})} = \sum_{m=1}^{Q} x_{l_{im}}^{(\text{w})}$ the number of clicks observed in the ith PPM frame, and by $\overline{\overline{y}}_i^{(\text{w})}$ the number of no-click events in the ith PPM frame within the detector dead time (where the click that triggered the detector reset may not necessarily be in the same frame). Equation (75) can thus be simplified as follows:

$$\begin{aligned}\frac{f_1(\mathbf{x}_{\text{w}})}{f_0(\mathbf{x}_{\text{w}})} &= \prod_{i=1}^{n/Q}\frac{1}{Q}\left[\frac{p_{\text{s}}^{(\text{w})}y_i^{(\text{w})}}{p_{\text{D}}^{(\text{w})}} + \overline{\overline{y}}_i^{(\text{w})} + \frac{\left(1-p_{\text{s}}^{(\text{w})}\right)\left(Q-y_i^{(\text{w})}-\overline{\overline{y}}_i^{(\text{w})}\right)}{1-p_{\text{D}}^{(\text{w})}}\right]\\ &= \prod_{i=1}^{n/Q}\left[1+\zeta p_{\text{r}}^{(\text{w})}\left(\frac{y_i^{(\text{w})}}{Qp_{\text{D}}^{(\text{w})}} + \frac{\overline{\overline{y}}_i^{(\text{w})}}{Q} - 1\right)\right].\end{aligned} \tag{76}$$

where equation (76) is obtained by noting that $\frac{p_{\text{s}}^{(\text{w})}}{p_{\text{D}}^{(\text{w})}} = \frac{\zeta p_{\text{r}}^{(\text{w})}(1-p_{\text{D}}^{(\text{w})})}{p_{\text{D}}^{(\text{w})}} + 1$ and $\frac{1-p_{\text{s}}^{(\text{w})}}{1-p_{\text{D}}^{(\text{w})}} = 1-\zeta p_{\text{r}}^{(\text{w})}$. Taking the logarithm of (76) yields the log-likelihood ratio

$$\ln\frac{f_1(\mathbf{x}_{\text{w}})}{f_0(\mathbf{x}_{\text{w}})} = \sum_{i=1}^{n/Q}\ln\left[1+\zeta p_{\text{r}}^{(\text{w})}\left(\frac{y_i^{(\text{w})}}{Qp_{\text{D}}^{(\text{w})}} + \frac{\overline{\overline{y}}_i^{(\text{w})}}{Q} - 1\right)\right]. \tag{77}$$

For small ζ, the Taylor series expansion of the summand in (77) at $\zeta = 0$ yields

$$\ln\left[1+\zeta p_{\mathrm{r}}^{(\mathrm{w})}\left(\frac{y_i^{(\mathrm{w})}}{Qp_{\mathrm{D}}^{(\mathrm{w})}}+\frac{\overline{\overline{y}}_i^{(\mathrm{w})}}{Q}-1\right)\right]\approx\zeta p_{\mathrm{r}}^{(\mathrm{w})}\left(\frac{y_i^{(\mathrm{w})}}{Qp_{\mathrm{D}}^{(\mathrm{w})}}+\frac{\overline{\overline{y}}_i^{(\mathrm{w})}}{Q}-1\right). \tag{78}$$

Since $\sum_{i=1}^{n/Q}\overline{\overline{y}}_i^{(\mathrm{w})} = Yt_{\mathrm{d}}$, where $Y = \sum_{i=1}^{n/Q} y_i^{(\mathrm{w})}$ is the total click count, the log-likelihood ratio can be approximated as follows:

$$\ln\frac{f_1(\mathbf{x}_{\mathrm{w}})}{f_0(\mathbf{x}_{\mathrm{w}})}\approx\frac{\zeta p_{\mathrm{r}}^{(\mathrm{w})}}{Qp_{\mathrm{D}}^{(\mathrm{w})}}(Y(1+p_{\mathrm{D}}^{(\mathrm{w})}t_{\mathrm{d}})-np_{\mathrm{D}}^{(\mathrm{w})}). \tag{79}$$

Since the approximation in (79) is an invertible function of the total click count Y, we use it as the test statistic for Willie. We also note that, because $p_{\mathrm{D}}^{(\mathrm{w})}t_{\mathrm{d}} \ll 1$ in our experiments, the dead time causes only a minimal performance degradation for Willie's detector.

Supplementary Note 6. Gaussian Approximation of $\mathbb{P}_{\mathrm{e}}^{(\mathrm{w})}$

At the end of the Supplementary Note 5 we argued that the detector dead time does not substantially impact Willie's performance, as the dead time is short relative to the average time between clicks. Here we show how setting $t_{\mathrm{d}} = 0$ yields a useful analytical approximation of $\mathbb{P}_{\mathrm{e}}^{(\mathrm{w})}$.

First consider the case when Alice is not transmitting. Then the total click count is a binomial random variable $Y \sim \mathcal{B}\left(y; p_{\mathrm{D}}^{(\mathrm{w})}, n\right)$ whose distribution, for large n, can be approximated using the central limit theorem by a Gaussian distribution $\Phi\left(y;\mu_0,\sigma_0^2\right)$ with $\mu_0 = np_{\mathrm{D}}^{(\mathrm{w})}$ and $\sigma_0^2 = np_{\mathrm{D}}^{(\mathrm{w})}\left(1-p_{\mathrm{D}}^{(\mathrm{w})}\right)$, where $\Phi\left(x;\mu,\sigma^2\right) = \frac{1}{\sqrt{2\pi}\sigma}\int_{-\infty}^{x} e^{-\frac{|t-\mu|^2}{2\sigma^2}}dt$ is the distribution function of a Gaussian random variable $\mathcal{N}(x;\mu,\sigma^2)$. Now consider the case when Alice is transmitting. Since $\mathcal{S}$ and $\mathbf{k}$ are unknown to Willie, the total click count is the sum of two independent but not identical binomial random variables $Y = X + Z$, where $X \sim \mathcal{B}\left(x; p_{\mathrm{D}}^{(\mathrm{w})}, n-\frac{n}{Q}\right)$ is the number of dark clicks in the $n-\frac{n}{Q}$ modes that Alice never uses in a PPM scheme and $Z \sim \mathcal{B}\left(z; p_{\mathrm{s}}^{(\mathrm{w})}, \frac{n}{Q}\right)$ is the contribution from the $\frac{n}{Q}$ modes that Alice can use to transmit, with $p_{\mathrm{s}}^{(\mathrm{w})}$ defined in (74). By the central limit theorem, for large n, the distribution of X can be approximated using a Gaussian distribution $\Phi(x;\mu_X,\sigma_X^2)$ where $\mu_X = \left(n-\frac{n}{Q}\right)p_{\mathrm{D}}^{(\mathrm{w})}$ and $\sigma_X^2 = \left(n-\frac{n}{Q}\right)p_{\mathrm{D}}^{(\mathrm{w})}\left(1-p_{\mathrm{D}}^{(\mathrm{w})}\right)$. Similarly, the distribution of Z can be approximated by a Gaussian distribution $\Phi\left(z;\mu_Z,\sigma_Z^2\right)$ where $\mu_Z = \frac{n}{Q}\left(\zeta p_{\mathrm{r}}^{(\mathrm{w})}+\left(1-\zeta p_{\mathrm{r}}^{(\mathrm{w})}\right)p_{\mathrm{D}}^{(\mathrm{w})}\right)$ and $\sigma_Z^2 = \frac{n}{Q}\left(\zeta p_{\mathrm{r}}^{(\mathrm{w})}+\left(1-\zeta p_{\mathrm{r}}^{(\mathrm{w})}\right)p_{\mathrm{D}}^{(\mathrm{w})}\right)\left(1-\zeta p_{\mathrm{r}}^{(\mathrm{w})}\right)\left(1-p_{\mathrm{D}}^{(\mathrm{w})}\right)$. Thus, the distribution of Y can be approximated by a Gaussian distribution $\Phi\left(y;\mu_1,\sigma_1^2\right)$ with $\mu_1 = \mu_X + \mu_Z$ and $\sigma_1^2 = \sigma_X^2 + \sigma_Z^2$ via the additivity property of independent Gaussian random variables. Willie's probability of error is thus approximated by:

$$\tilde{\mathbb{P}}_{\mathrm{e}}^{(\mathrm{w})} = \frac{1}{2}\min_{S}(1-\Phi(S;\mu_0,\sigma_0^2)+\Phi(S;\mu_1,\sigma_1^2)). \tag{80}$$

The value of the threshold S^* that minimizes the RHS of (80) satisfies $\frac{|S^*-\mu_0|^2}{\sigma_0^2} - \log(\sigma_1^2/\sigma_0^2) = \frac{|S^*-\mu_1|^2}{\sigma_1^2}$.

Supplementary Note 7. Analysis of the Detector Dark Clicks

Here we provide the detailed analysis of detector dark clicks, focusing on how their temporal variation affected our experiments. While we took great care in maintaining uniform conditions throughout our

experiments, controlling every aspect of our environment was beyond our capabilities. However, we argue that the temporal variation in the dark click probability that we experienced had no significant impact on our results.

We maximize the logarithm of the likelihood function in equation (71) and obtain the following maximum likelihood estimator of the dark click probability:

$$\hat{p}_{\mathrm{D}} = \frac{\sum_{i=1}^{n_{\mathrm{D}}} x_i}{n_{\mathrm{D}} - t_{\mathrm{d}} \sum_{i=1}^{n_{\mathrm{D}}} x_i}, \tag{81}$$

where $x_1, \ldots, x_{n_{\mathrm{D}}}$ is the sequence of n_{D} observations where only the dark clicks can be observed, i.e, it is the experimental click record that excludes the observations of Alice's transmissions as well as the dead time following the detected transmissions. The entire click record contains 100 experiments at each value of n for both Alice using and not using the channel, totaling 2.72×10^{10} observations when $\zeta = 0.25/\sqrt{n}$ and 3.0784×10^{10} observations when $\zeta \in \{0.03/\sqrt[4]{n}, 0.003, 0.008\}$. For each of the four communication regimes, we divide the click record (sorted by time) into segments of $n_{\mathrm{D,s}} = 3.2 \times 10^7$ consecutive observations. The estimates of Willie's and Bob's dark click probabilities for these segments are denoted by $\hat{p}^{(\mathrm{w})}_{\mathrm{D,s}}(j)$ and $\hat{p}^{(\mathrm{b})}_{\mathrm{D,s}}(j)$, $j = 1, \ldots, n_{\mathrm{S,s}}(\zeta)$, where $n_{\mathrm{S,s}}(0.25/\sqrt{n}) = 850$ and $n_{\mathrm{S,s}}(0.03/\sqrt[4]{n}) = n_{\mathrm{S,s}}(0.003) = n_{\mathrm{S,s}}(0.008) = 962$. The plots of $\hat{p}^{(\mathrm{w})}_{\mathrm{D,s}}(j)$ and $\hat{p}^{(\mathrm{b})}_{\mathrm{D,s}}(j)$ in Supplementary Figure 1 illustrate the temporal variations in dark click probability. However, they also show homogeneity over relatively long periods of time. We thus estimate the dark click probability for blocks of 37 consecutive segments using $n_{\mathrm{D,b}} = 1.184 \times 10^9$ observations (except for the last, 23rd, block of the click record for $\zeta = 0.25/\sqrt{n}$ containing 36 ($n_{\mathrm{D,b}} = 1.152 \times 10^9$ observations) segments instead of 37). The estimates of Willie's and Bob's dark click probabilities for these blocks are denoted by $\hat{p}^{(\mathrm{w})}_{\mathrm{D,b}}(k)$ and $\hat{p}^{(\mathrm{b})}_{\mathrm{D,b}}(k)$, $k = 1, \ldots, n_{\mathrm{S,b}}(\zeta)$, where $n_{\mathrm{S,b}}(0.25/\sqrt{n}) = 23$ and $n_{\mathrm{S,b}}(0.03/\sqrt[4]{n}) = n_{\mathrm{S,b}}(0.003) = n_{\mathrm{S,b}}(0.008) = 26$. The block dark click probability estimate is close to the average of the estimates for its component segments, and over most of the 101 blocks, the segment estimates of the dark click probability are homogeneous (assuming that the dark click probability stays constant over the period of time corresponding to a segment, the number of observed dark clicks would be binomially-distributed if our detectors had zero dead time. However, dead time has a minimal impact on our experiment, making the binomial distribution a good approximation for the distribution of the number of observed dark clicks. Thus, for each of blocks, we performed the Pearson chi-squared test for homogeneity [23] in the estimated dark click probability of its component segments. We found that the test rejects the null hypothesis (that the estimates are homogeneous) in only 29 out of 202 blocks, consistent with the visual inspection of Supplementary Figure 1.)

We also plot $\hat{p}^{(\mathrm{w})}_{\mathrm{D,b}}(j)$ versus $\hat{p}^{(\mathrm{b})}_{\mathrm{D,b}}(j)$ in Supplementary Figure 2, revealing strong correlation between the dark click probabilities of Bob's and Willie's detectors. Thus the temporal variations in the dark click probabilities likely stem from the external environmental factors (such as laboratory temperature changes) rather than the detectors themselves.

Intuitively, clicks observed under less noisy channel conditions carry more evidence for the hypothesis that Alice is transmitting than clicks observed when the channel is noisier. Indeed, in the derivation of the total click count Y as Willie's test statistic, the contribution to Y from the ith channel observation $x_i^{(\mathrm{w})} \in \{0, 1\}$ is effectively weighted by $1/p_{\mathrm{D}}^{(\mathrm{w})}$ (we ignore the term corresponding to the detector dead time for simplicity of exposition and since it has no tangible impact on our experimental results). In the analysis of our experiment we used the average dark click probability, however, if the dark click probability $p_{\mathrm{D}}^{(\mathrm{w})}(i)$ is available for $i = 1, \ldots, n$, $Y_{\text{weighted}} = \sum_{i=1}^{n} \frac{x_i^{(\mathrm{w})}}{p_{\mathrm{D}}^{(\mathrm{w})}(i)}$ is a better test statistic. Since the exact $p_{\mathrm{D}}^{(\mathrm{w})}(i)$ is unavailable, and since the estimates are (mostly) homogeneous over the duration of the blocks, we study the impact of temporal variation of dark clicks on Willie's detection error by weighting the observations by the block estimates $\hat{p}^{(\mathrm{w})}_{\mathrm{D,b}}(j)$. Denoting the set of observations in the jth block by $\mathcal{W}_j$, we block-weight Willie's test statistic as follows:

$$Y_{\text{block-weighted}} = \sum_{j=1}^{n_{\mathrm{S,b}}(\zeta)} \frac{1}{\hat{p}^{(\mathrm{w})}_{\mathrm{D,b}}(j)} \sum_{x_i^{(\mathrm{w})} \in \mathcal{W}_j} x_i^{(\mathrm{w})}. \tag{82}$$

We plot the estimates of detection error probability that are calculated using the block-weighted test statistic given by (82) in Supplementary Figure 3 alongside the estimates from Figure 4 that are calculated using the (unweighted) total click count. While the estimated probability of detection error decreases in some cases (and increases in others), the overall impact is small. The square-root scaling law is unaffected since Alice and Bob can design their covert communication using a lower bound on $p_{\mathrm{D}}^{(\mathrm{w})}$ (for example, the dark click probability for the best available photon detector operating in near-ideal conditions). However, since the random fluctuations in noise power have been shown to yield positive-rate covert communication in AWGN channel setting [24, 25], Alice and Bob could potentially exploit the random process governing $p_{\mathrm{D}}^{(\mathrm{w})}$ to transmit covert information at a positive rate.

Supplementary Note 8. Calculation of the Number of Quantum-Secure Covert Bits in Figure 5

The objective function (4) in the Methods is the total number of reliably transmitted covert bits. We obtain it by equating the RHS of the generalized version of (49) to the desired decoding error probability δ and solving for M, yielding:

$$M = \sum_{i=1}^{n} \frac{1}{2} \log_2 \left(1 + \frac{\bar{n}_i}{2\sigma_i^2}\right) + \log_2 \delta. \tag{83}$$

Thus, (49) generalizes to non-identical channels by summing the contributions from each spatio-temporal-polarization mode (indexed by i) to the total number of transmitted covert bits. Since $\log_2 \delta$ is a constant, we discard it from optimization. The term inside the sum in (83) is the amount of information transmitted per mode, which we measure in nats for convenience:

$$R(\bar{n}_q, \eta_q, \bar{n}_{\mathrm{T}}) = \frac{1}{2} \ln \left(1 + \frac{\bar{n}_q}{s_q}\right), \tag{84}$$

where

$$s_q = \frac{2(1-\gamma_q)\bar{n}_{\mathrm{T}} + 1}{2\gamma_q}, \tag{85}$$

which is $2\sigma_b^2$, with σ_b^2 defined in (46). The per-mode contribution to the total quantum relative entropy (QRE) is upper-bounded by (45) and is thus given by:

$$D(\bar{n}_q, \gamma_q, \bar{n}_{\mathrm{T}}) = d_q \bar{n}_q^2, \tag{86}$$

where

$$d_q = \frac{(1-\gamma_q)^2}{2\gamma_q \bar{n}_{\mathrm{T}}(1+\gamma_q \bar{n}_{\mathrm{T}})}. \tag{87}$$

Substituting (84) and (86) into (4) and (5) in the Methods, respectively, yields the following form of the optimization problem:

$$\underset{(\bar{n}_q)_{q\in\mathbb{Z}^+}}{\text{maximize}}\ TW \sum_{q=1}^{\infty} q \ln \left(1 + \frac{\bar{n}_q}{s_q}\right) \tag{88}$$

$$\text{subject to } 2TW \sum_{q=1}^{\infty} q d_q \bar{n}_q^2 \leq C \tag{89}$$

$$\bar{n}_q \geq 0, \tag{90}$$

where $C = 8\epsilon^2$. This is a variant of the standard problem of optimal power allocation on parallel AWGN channels [19, Section 9.4], with the differences being the sum of squares of power instead of the total power in the constraint and the infinite number of available channels. Recalling that, for each $q = 1, 2, \ldots$, there are q parallel channels available, the sequence describing the total power allocation is $(q\bar{n}_q)_{q\in\mathbb{Z}^+}$. While it is infinite, $(q\bar{n}_q)_{q\in\mathbb{Z}^+} \in \ell^1$ since practical systems are peak power constrained. It is straightforward to show that the objective function and the constraints are Fréchet-differentiable (for example, one can employ the continuity of the respective Gâteaux derivatives). The optimal power allocation $(\bar{n}_q^*)_{q\in\mathbb{Z}^+}$ is thus a stationary point of a Lagrange functional, which we write as follows:

$$\Lambda = TW \sum_{q=1}^{\infty} q \ln\left(1 + \frac{\bar{n}_q}{s_q}\right) - \lambda\left(2TW \sum_{q=1}^{\infty} q d_q \bar{n}_q^2 - 8\epsilon^2\right). \tag{91}$$

The stationary point is unique since the objective function is strictly concave and the constraints are strictly convex. It is obtained by differentiating the Lagrangian functional with respect to $\bar{n}_q$, and setting the result to zero:

$$\frac{\partial \Lambda}{\partial \bar{n}_q} = \frac{TWq}{s_q + \bar{n}_q} + 4\lambda TW q d_q \bar{n}_q = 0. \tag{92}$$

There are two possible solutions of (92) for $\bar{n}_q$: $\frac{1}{2}\left(-s_q \pm \frac{\sqrt{\lambda^2 d_q^2 s_q^2 + \lambda d_q}}{\lambda d_q}\right)$, however, we discard one of them since $\frac{1}{2}\left(-s_q - \frac{\sqrt{\lambda^2 d_q^2 s_q^2 + \lambda d_q}}{\lambda d_q}\right) \leq 0$ or complex. We also note that the other solution is positive for $\lambda > 0$. We use the Karush–Kuhn–Tucker (KKT) conditions to verify that the assignment

$$\bar{n}_q^* = \frac{1}{2}\left(\sqrt{s_q^2 + \frac{1}{\lambda d_q}} - s_q\right), \tag{93}$$

maximizes (88). Since an interpretation of the Lagrange multiplier λ is the sensitivity of the solution to the constraint C, $\lambda > 0$ matches our intuition, as higher tolerance to detection allows greater number of transmitted bits. Let us also verify that $(q\bar{n}_q^*)_{q\in\mathbb{Z}^+} \in \ell^1$:

$$\sum_{q=1}^{\infty} |q\bar{n}_q^*| = \frac{1}{2}\sum_{q=1}^{\infty} q\sqrt{s_q^2 + \frac{1}{\lambda d_q}} - s_q \tag{94}$$

$$\leq \frac{1}{4\lambda}\sum_{q=1}^{\infty} \frac{q}{d_q s_q} \tag{95}$$

$$= \frac{1}{4\lambda}\sum_{q=1}^{\infty} \frac{q\gamma_q^2 \bar{n}_{\mathrm{T}}(1 + \gamma_q \bar{n}_{\mathrm{T}})}{(2(1-\gamma_q)\bar{n}_{\mathrm{T}} + 1)(1-\gamma_q)^2} \tag{96}$$

$$\leq \frac{\bar{n}_{\mathrm{T}}(1 + \bar{n}_{\mathrm{T}})}{4\lambda}\sum_{q=1}^{\infty} \frac{q\gamma_q^2}{(1-\gamma_q)^2} < \infty, \tag{97}$$

where (95) is because $\sqrt{1+x} - 1 \leq \frac{x}{2}$ for $x \geq -1$ by Bernoulli's inequality, (96) is from substituting (85) and (87) into (95), (97) is because $\gamma_q \leq 1$ and $2(1-\gamma_q)\bar{n}_{\mathrm{T}} + 1 \geq 1$. Finally, to show that the sum in (97) converges, we employ the limit comparison test: $\lim_{q\to\infty} \frac{1}{(1-\gamma_q)^2} = 1$ and $\sum_{q=1}^{\infty} q\gamma_q^2 < \infty$.

Now we substitute (93) into the objective function (88) and verify that the objective function converges:

$$TW \sum_{q=1}^{\infty} q \ln\left(1 + \frac{\bar{n}_q}{s_q}\right) = TW \sum_{q=1}^{\infty} q \ln\left(1 + \frac{1}{2}\left(\sqrt{1 + \frac{1}{\lambda d_q s_q^2}} - 1\right)\right) \tag{98}$$

$$\leq TW \sum_{q=1}^{\infty} q \ln\left(1 + \frac{1}{4\lambda d_q s_q^2}\right) \tag{99}$$

$$\leq \frac{TW}{4\lambda} \sum_{q=1}^{\infty} \frac{q}{d_q s_q^2} < \infty, \tag{100}$$

where (99) is because of Bernoulli's inequality, (100) is because $\ln(1+x) \leq x$ for $x \geq -1$, and the convergence of the sum follows from $s_q \geq 1$ and arguments for (96) and (97).

We thus need to find λ that satisfies:

$$2TW \sum_{q=1}^{\infty} \frac{q d_q}{4} \left(\sqrt{s_q^2 + \frac{1}{\lambda d_q}} - s_q\right)^2 = C. \tag{101}$$

While we can verify the existence of λ by demonstrating that the left-hand side (LHS) of (101) converges (using arguments similar to those showing that (94) and (98) converge), the summation in (101) does not have a known closed-form solution. We thus solve it for λ numerically using `fzero` function in MATLAB Optimization Toolbox. Since the transmissivity γ_q decreases exponentially with q because of the increase in diffraction-limited loss, there exists $q_{\max}$ such that the additional benefit to the covert volume from using spatial modes with index $q > q_{\max}$ is negligible. This fact, coupled with the sensitivity of `fzero` to the starting point, motivated us to employ the following iterative procedure for calculating the covert volume reported in Figure 5:

$\lambda_0 \leftarrow \frac{s_1\sqrt{\frac{d_1 C}{2TW}} - \frac{C}{2TW}}{\frac{d_1 s_1^2 C}{TW} - \frac{C}{2T^2W^2}}$ ▷ Inverse of (101) with only a single term ($q = 1$) in summation
$\bar{n}_1 = \sqrt{\frac{C}{d_1 TW}}$ ▷ Mean photon number when using only a single (Gaussian) spatial mode ($q = 1$)
$\tau_{\text{prev}} \leftarrow TW \log_2\left(1 + \frac{\bar{n}_1}{s_1}\right)$ ▷ Initialize sum covert volume using a single (Gaussian) spatial mode
$q_{\max} = 1$
loop
  $q_{\max} = q_{\max} + 1$
  $\lambda \leftarrow$ `fzero` $\left(2TW \sum_{q=1}^{q_{\max}} \frac{q d_q}{4}\left(\sqrt{s_q^2 + \frac{1}{\lambda d_q}} - s_q\right)^2 - C, \lambda, \lambda_0\right)$ ▷ Solve for λ using initial value λ_0
  $\lambda_0 \leftarrow \lambda$ ▷ Next call to `fzero` (for $q_{\max} + 1$) will be initialized with previous solution
  for $q \leftarrow 1$ **to** $q_{\max}$ **do**
    $\bar{n}_q \leftarrow \frac{1}{2}\left(\sqrt{s_q^2 + \frac{1}{\lambda d_q}} - s_q\right)$ ▷ Calculate mean photon number using (93)
  end for
  $\tau \leftarrow TW \sum_{q=1}^{q_{\max}} q \ln\left(1 + \frac{\bar{n}_q}{s_q}\right)$
  if $\tau \approx \tau_{\text{prev}}$ **then** ▷ We used fuzzy comparison $|\tau - \tau_{\text{prev}}| \leq 10^{-6}$
    break
  end if
  $\tau_{\text{prev}} \leftarrow \tau$
end loop
return τ_{prev}

Supplementary References

[1] D. Slepian, "Prolate spheroidal wave functions, fourier analysis and uncertainty–iv: Extensions to many dimensions; generalized prolate spheroidal functions," Bell Syst. Tech. J. **43**, 3009–3057 (1964).

[2] D. Slepian, "Analytical solution to two apodization problems," J. Opt Soc. Am. **55**, 1110–1114 (1965).

[3] J. H. Shapiro, S. Guha, and B. I. Erkmen, "Ultimate channel capacity of free-space optical communications," Journal of Optical Networking **4**, 501–516 (2005).

[4] Gregorius C. G. Berkhout, Martin P. J. Lavery, Johannes Courtial, Marco W. Beijersbergen, and Miles J. Padgett, "Efficient sorting of orbital angular momentum states of light," Phys. Rev. Lett. **105**, 153601 (2010).

[5] N. Chandrasekaran and J.H. Shapiro, "Photon information efficient communication through atmospheric turbulence–part i: Channel model and propagation statistics," J. Lightw. Technol. **32**, 1075–1087 (2014).

[6] N. Chandrasekaran, J.H. Shapiro, and Ligong Wang, "Photon information efficient communication through atmospheric turbulence–part ii: Bounds on ergodic classical and private capacities," J. Lightw. Technol. **32**, 1088–1097 (2014).

[7] Horace P. Yuen and J.H. Shapiro, "Optical communication with two-photon coherent states–part i: Quantum-state propagation and quantum-noise," IEEE Transactions on Information Theory **24**, 657–668 (1978).

[8] J.H. Shapiro, Horace P. Yuen, and A. Mata, "Optical communication with two-photon coherent states–part ii: Photoemissive detection and structured receiver performance," IEEE Transactions on Information Theory **25**, 179–192 (1979).

[9] Horace P. Yuen and J.H. Shapiro, "Optical communication with two-photon coherent states–part iii: Quantum measurements realizable with photoemissive detectors," IEEE Transactions on Information Theory **26**, 78–92 (1980).

[10] Harry L. Van Trees, *Detection, Estimation, and Modulation Theory, Part I: Detection, Estimation, and Linear Modulation Theory* (John Wiley & Sons, Inc., New York, 2001).

[11] M.M. Wilde, *Quantum Information Theory* (Cambridge University Press, 2013).

[12] Richard A. Campos, Bahaa E. A. Saleh, and Malvin C. Teich, "Quantum-mechanical lossless beam splitter: SU(2) symmetry and photon statistics," Phys. Rev. A **40**, 1371–1384 (1989).

[13] Carl W. Helstrom, *Quantum Detection and Estimation Theory* (Academic Press, Inc., New York, NY, USA, 1976).

[14] Stefano Pirandola and Seth Lloyd, "Computable bounds for the discrimination of gaussian states," Phys. Rev. A **78**, 012331 (2008).

[15] V. Giovannetti, S. Guha, S. Lloyd, L. Maccone, J. H. Shapiro, and H. P. Yuen, "Classical capacity of the lossy bosonic channel: The exact solution," Phys. Rev. Lett. **92**, 027902 (2004).

[16] Boulat A. Bash, Dennis Goeckel, and Don Towsley, "Limits of reliable communication with low probability of detection on AWGN channels," IEEE Journal on Selected Areas in Communications **31**, 1921–1930 (2013).

[17] Thomas H. Cormen, Charles E. Leiserson, Ronald L. Rivest, and Clifford Stein, *Introduction to Algorithms*, 2nd ed. (MIT Press, Cambridge, Massachusetts, 2001).

[18] Erich Lehmann and Joseph Romano, *Testing Statistical Hypotheses*, 3rd ed. (Springer, New York, NY, USA, 2005).

[19] Thomas M. Cover and Joy A. Thomas, *Elements of Information Theory*, 2nd ed. (John Wiley & Sons, Hoboken, NJ, USA, 2002).

[20] Robert G. Gallager, *Information Theory and Reliable Communication* (John Wiley and Sons, Inc., New York, 1968).

[21] A. S. Holevo, "The capacity of the quantum channel with general signal states," IEEE Trans. Inf. Theory **44**, 269–273 (1998).

[22] Claude E. Shannon, "A mathematical theory of communication," Bell Syst. Tech. J. **27** (1948).

[23] P.E. Greenwood and M.S. Nikulin, *A guide to chi-squared testing* (Wiley, New York, NY, 1996).

[24] Seonwoo Lee and R.J. Baxley, "Achieving positive rate with undetectable communication over AWGN and Rayleigh channels," in *Proc. IEEE Int. Conf. Commun. (ICC)* (2014) pp. 780–785.

[25] Seonwoo Lee, R.J. Baxley, J.B. McMahon, and R.S. Frazier, "Achieving positive rate with undetectable communication over mimo rayleigh channels," in *IEEE Sensor Array and Multichannel Signal Processing Workshop (SAM)* (2014) pp. 257–260.
